# Molecular Systems Architecture of Fibrotic Lung Microenvironment in Idiopathic Pulmonary Fibrosis

**DOI:** 10.3390/cells15151364

**Published:** 2026-07-29

**Authors:** V. A. Shiva Ayyadurai, Yamuna Manoharan, Prabhakar Deonikar

**Affiliations:** 1 Systems Biology Group, CytoSolve Research Division, CytoSolve, Inc., Cambridge, MA 02138, USA; yamuna@vashiva.com; 2 The Open Science Institute, International Center for Integrative Systems, Cambridge, MA 02138, USA

**Keywords:** IPF, CytoSolve^®^, systems biology, fibroblast, AECII, myofibroblast, TGF-β, extracellular matrix, ECM, macrophage, epithelial–mesenchymal transition, fibroblast-myofibroblast transition, and tissue remodeling

## Abstract

**Background:** Idiopathic pulmonary fibrosis (IPF) is a progressive and irreversible fibrosing interstitial lung disease characterized by excessive extracellular matrix (ECM) accumulation, disruption of lung architecture, and progressive loss of pulmonary function. IPF is frequently accompanied by comorbid conditions that exacerbate disease progression and negatively impact prognosis. To address the biological complexity of IPF, this study presents a comprehensive molecular systems architecture that enables a system-level understanding of biomolecular interactions within the fibrotic lung microenvironment in response to external and physiological triggers. **Methods:** A literature search is conducted using the Medical Subject Headings (MeSH) keywords in PubMed and MEDLINE to identify relevant peer-reviewed articles published from April 2008 to June 2025, with Google Scholar used solely to retrieve full-text versions of articles identified through this search. The systems biology tool CytoSolve^®^ was used to perform the systematic review and to support the curation and development of the molecular systems architecture of IPF pathogenesis. Full-length articles that contained Medical Subject Headings keywords relevant to IPF pathogenesis were selected for a comprehensive review. A total of 150 studies published between April 2008 and June 2025 met the inclusion criteria and were included in the systematic analysis. This systematic review was not registered. **Results:** Findings were synthesized qualitatively into a multilayered molecular interactome rather than through statistical meta-analysis. The architecture integrates interactions across sixteen lung-associated cell types, including epithelial, endothelial, mesenchymal, immune, and stromal populations. Key external triggers—such as bleomycin (BLM), asbestos, silica, radiation, cigarette smoke, Herpes virus, and genetic mutations (SFTPC I73T), along with hypoxia associated with comorbidities—initiate coordinated cellular responses that converge on three fundamental pathological processes: inflammation, myofibroblast differentiation, and tissue remodeling. These interconnected processes collectively drive the initiation and progression of IPF. **Conclusions:** This molecular systems architecture unifies triggers, cellular components, molecular pathways, and biological processes into a multilayered framework for identifying therapeutic targets, biomarkers, and rational single- and combination-treatment strategies in IPF.

## 1. Introduction

Idiopathic pulmonary fibrosis (IPF) is one of the most progressive interstitial lung diseases, and current therapeutics are known to slow disease progression, but not cure the disease [1]. Even though extensive research has been conducted for the last 50 years, including nearly 62,023 studies, there has been no significant progress in therapeutic intervention for IPF [2]. This study aims to inspire the field to embrace systems biology approaches to advance IPF research. Specifically, this study hypothesizes that the development of a molecular systems architecture of the fibrotic lung microenvironment is necessary for such advancement.

**Figure 1:**
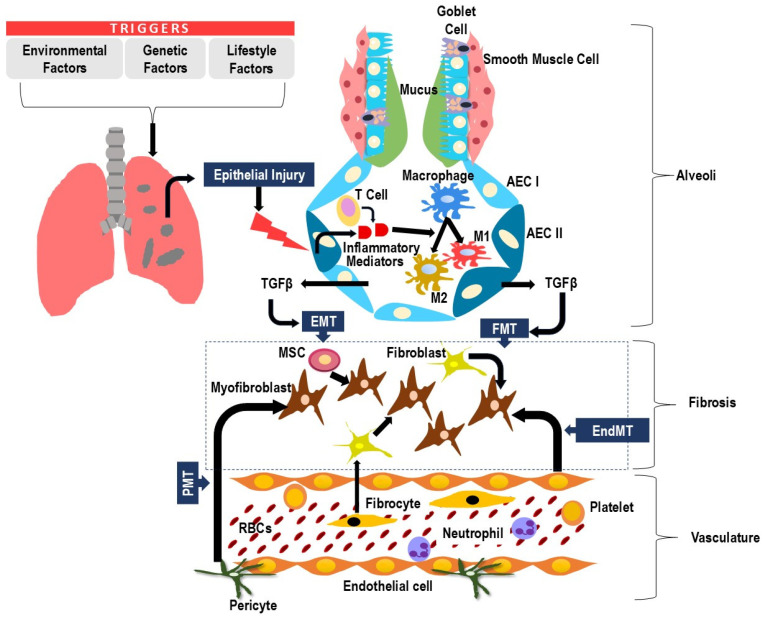
The fibrotic lung microenvironment in IPF. Environmental and genetic factors damage the distal airway epithelium, releasing proinflammatory mediators that recruit immune cells such as alveolar macrophages and CD4^+^ T cells. TGF-β released by injured AECII and polarized M2 macrophages drives the differentiation of epithelial, pericyte, fibroblast, and endothelial cells into myofibroblasts via EMT, PMT, FMT, and EndMT, respectively. Myofibroblast-derived ECM proteins, including collagen and α-SMA, accumulate in the alveolar region, disrupting alveolar architecture and impairing mucociliary clearance and epithelial barrier integrity. Abbreviations are defined in Supplementary Material S5 Glossary.

The fibrotic lung microenvironment in IPF is illustrated in Figure 1. This microenvironment is the locus wherein fibrosis of the lung manifests, and comprises proximal airways including ciliated, secretory, and mucus-producing goblet epithelial cells, and distal airways including alveoli. Although Figure 1 presents the fibrotic lung microenvironment as a schematic systems-level framework, it does not quantitatively resolve the spatial organization of cells within lung tissue. Structural cell types in the fibrotic lung microenvironment include Type I alveolar epithelial cells (AECI), Type II alveolar epithelial cells (AECII), pulmonary smooth muscle cells, mesenchymal stem cells, fibroblasts, pericytes, and endothelial cells. The distal airways in the IPF lung are shown schematically. A molecular systems architecture of this microenvironment may: (1) enable the visualization of complex bimolecular systems interactions across the fibrotic lung microenvironment involving sixteen cell types; (2) reveal the biological processes and the underlying molecular pathways leading to disease pathogenesis; (3) identify potential targets for treatment; and (4) provide a framework to develop predictive and quantitative models for drug development.

Systems biology explores the interactions in living systems at multiple levels, including molecular, cellular, tissue, organ, and environmental levels, to elucidate physiological processes [3]. A systems biology approach aids analyses of disease conditions by quantitatively examining disrupted cellular signaling cascades that cause gain or loss of function, thereby developing targeted therapies [4]. Reductionist strategies still dominate current drug development, but this paradigm carries notable limitations, including a limited capacity to design curative combination therapies, an inability to fully capture the complexity of molecular interactions, and a shortage of computational tools capable of predicting systems-level adverse effects [5,6]. Systems biology deciphers complex biochemical networks across cellular systems, driving disease pathways and clinical phenotypes, enabling the discovery of targeted therapies. It offers a new drug development paradigm, enhancing efficacy via combinations with approved drugs [7,8].

Molecular pathways in complex diseases rarely operate in isolation; instead, they form networked systems that extend across multiple organs, cell types, organelles, and subcellular compartments. To capture this complexity, the authors have previously established a systems biology methodology for identifying, aggregating, and computationally integrating cell-level signaling cascades into unified molecular systems architectures. This approach has previously yielded systems-level insight into neurovascular disease [9], acute myeloid leukemia (AML) [8], periodontal disease [6], C1 metabolism in plants [10], low-grade chronic inflammation [11], liver detoxification [12], amyotrophic lateral sclerosis (ALS) [13], mesenchymal stromal cells [14], and asthma [15].

IPF is a chronic, progressive fibrosing interstitial pneumonia of unknown etiology primarily affecting the lung interstitium [16]. Such chronic fibrosis is characterized by the replacement of healthy lung tissue by excess ECM, leading to disturbed gas exchange, respiratory failure, and consequent mortality [17]. IPF arises via a “two-hit” mechanism combining genetic predisposition and environmental factors, triggering epithelial injury [16]. Studies across 12 countries (January 2009–April 2020) report global IPF incidence of 0.09–1.30 cases per 10,000 people and prevalence of 0.33–4.51 per 10,000 [18]. IPF predominates in men, with a median diagnosis at age 65 and post-diagnosis survival of 2–5 years [19].

Though IPF’s exact cause remains unknown, multiple risk factors predispose to recurrent alveolar epithelial injury, initiating disease development [16]. These include genetic alterations, chronic viral infections, environmental pollutants, animal antigens, cigarette smoking, psychological factors, and comorbidities [20,21,22]. A large cohort study of 9000 IPF patients links the disease to comorbidities, including pulmonary hypertension, emphysema, lung cancer, gastroesophageal reflux, diabetes mellitus, venous thromboembolism, coronary artery disease, congestive heart failure, sleep-disordered breathing, anxiety, and depression [23,24]. Occupational exposures (agricultural chemicals, wood/silica/metal dust) and viral infections (EBV, CMV, HHV-7, HHV-8) increase IPF risk. IPF patients exhibit elevated EBV/CMV loads and EBV antigens in alveolar epithelial cells [17,21].

AECII, which comprise ~60% of alveolar cells, produce surfactant to prevent alveolar collapse during breathing and serve as progenitors that regenerate damaged epithelium by differentiating into AECI [16]. Lung epithelial cell-derived surfactants, comprising proteins (SPC, SPB, SPA, SPD) and phospholipids, reduce alveolar surface tension, inflammation, and facilitate pathogen clearance [25]. Mutations in surfactant protein genes cause dysfunction, leading to respiratory distress and initiation of IPF [24]. Mitochondrial fusion proteins Mfn1 and Mfn2 regulate surfactant production in AECII cells by modulating the lipid metabolism involved in cholesterol and phospholipid synthesis. Their loss impairs epithelial barrier integrity and promotes fibrosis [26]. Recent evidence suggests that inflammation can be a secondary event that occurs simultaneously, following the onset of the primary fibrotic remodeling process, mediated by interactive signaling among dysfunctional epithelial cells, activated fibroblasts, and endothelial cells [27,28]. Studies show that injured AECII in IPF exhibit aging hallmarks such as telomere attrition, genomic instability, senescence, and mitochondrial dysfunction, which accelerate lung aging and fibrosis [20,29].

Research studies indicate that genetic predisposition combined with environmental triggers increases the risk of IPF, leading to sporadic or familial IPF [21,30]. Mutations in epithelial genes involved in telomere maintenance and surfactant and mucin production critically impair alveolar epithelium, contributing to both sporadic and familial IPF [20,31]. Histopathological features of IPF are characterized by ECM accumulation around activated fibroblasts and myofibroblasts (fibroblast foci), adjacent to hyperplastic and apoptotic AECII, as illustrated in Figure 1 [32]. Myofibroblasts are key effector cells driving IPF. They localize to active fibrotic sites, producing ECM and destroying alveolar structure [33]. Precursor cells for myofibroblasts include AECII, resident fibroblasts, endothelial cells, pericytes, and fibrocytes [34]. Recent research reports that 16% of effector myofibroblasts derive from endothelial cells via endothelial-to-mesenchymal transition (EndMT) in IPF [22]. Among IPF animal models induced by triggers like BLM, silica, and radiation, the BLM mouse model is most widely studied for elucidating IPF pathogenesis [19]. The BLM model recapitulates pathological features observed in IPF patients and aids in the identification of molecular mechanisms for which therapeutic strategies are developed [19,35].

Two antifibrotic drugs, nintedanib and pirfenidone, are currently used in IPF therapeutic management [36]. These antifibrotics slow IPF progression and improve lung function to some extent, but cannot reverse the structural damage of the IPF lung [34,36]. Lung transplantation, another established option, has been reported to be associated with elevated mortality risks [30]. Furthermore, anti-depression interventions such as cognitive behavioral therapy (CBT), music therapy, and meditation merely delay IPF lung deterioration [37].

A systems biology methodology was developed in this study to perform a systematic literature review of the current understanding of the fibrotic lung microenvironment in IPF, including associated comorbidities. The aim was to construct a molecular systems architecture—a visual, multi-layered interactome depicting molecular interactions between lung structural cells and immune cells. In IPF, the anatomical components of the fibrotic lung microenvironment, as depicted in Figure 1, are essential for building this architecture. This study offers the IPF research community an integrative molecular systems view of complex biomolecular interactions, triggered by environmental exposures, drugs, genetics, and comorbidities in pathogenesis of lung fibrosis. The resulting architecture reveals complex interactions among dysregulated lung cells, linking them to the remodeling of the lung tissue and functional decline. Fibrotic lungs cause increased stiffness and pulmonary hypertension—the key IPF manifestations underlying respiratory and cardiac dysfunction, respectively.

Rather than treating IPF as a set of isolated linear pathways, the architecture developed here represents the disease as a dynamic, multi-scale network of direct cell–cell crosstalk and emergent interactions. Framed this way, the architecture is positioned to accelerate therapeutic development by supporting computational modeling that tailors combination-drug strategies to individual disease severity. This study hypothesized that the development of a molecular systems architecture of the fibrotic lung microenvironment in IPF will provide the following outcomes:

1. Multi-layered visual map of biomolecular interactions in the fibrotic lung microenvironment;
2. Understanding of the complex crosstalk in the fibrotic lung microenvironment;
3. Potential targets for drug development;
4. Framework to develop computational models providing quantitative predictions.

### Significance Statement

Given the intricate, multi-cell-type interactions that drive IPF pathogenesis, a systems biology approach offers a powerful means of building a systems-level understanding of the disease. A molecular systems architecture is developed to generate a multi-layered visual map illustrating interactions between external and internal physiological triggers and biomolecular networks in the fibrotic lung microenvironment that drive IPF pathogenesis. This architecture also reveals complex cellular crosstalk between the lung and central nervous system (CNS) microenvironments, driven by pulmonary hypoxia from obstructive sleep apnea (OSA) comorbidity, which amplifies both lung fibrosis and neuroinflammation, contributing to psychological comorbidities. In the future, this architecture will enable predictive, quantitative computational models to identify combination therapies, clinical solutions, and strategies to minimize adverse effects.

## 2. Methodology

The scientific literature was systematically searched to identify relevant journal articles reporting on (1) the pathophysiology of IPF; (2) molecular interactions of external and internal triggers with lung structural cells; (3) molecular signaling pathways mediating cellular crosstalk between lung structural cells and resident immune cells; and (4) pathobiological pathways linked to psychological comorbidities in IPF. CytoSolve^®^ (Cambridge, MA, USA), a systems biology tool used in this study, supports the curation, organization, and collaborative review of scientific articles to enable systematic bioinformatics literature reviews. The CytoSolve methodology herein has been successfully applied in previous studies spanning diverse areas in systems biology, including osteoarthritis [38], neurovascular diseases [9], acute myeloid leukemia [8], periodontitis [6], amyotrophic lateral sclerosis (ALS) [13], mesenchymal stromal cells [14], and asthma [16]. This methodology is documented in detail in the previously published *CytoSolve Operating Guide* [6,8,11].

CytoSolve employs a distributed approach in which a large-scale biological system is decomposed into an ensemble of smaller molecular-pathway models that are computationally coupled, rather than modeled monolithically. The complete six-step CytoSolve^®^ System Protocol is described in Supplementary Material S4. The present study applies the first two steps of this protocol: systematic curation of the scientific literature and extraction of the molecular pathways it reports. The extracted pathways were then assembled into a single multi-cellular interactome by connecting shared molecular species across cell-type boundaries, such that a species produced in one cell serves as input to the connected pathway in a neighboring cell type. This assembly yields the molecular systems architecture presented as an interactome, described further in the Section 3. The remaining steps of the protocol constitute the quantitative modeling phase: the construction of individual mathematical models, their computational integration through the CytoSolve engine, and simulation of the integrated model. These steps are the focus of future work and are not undertaken in the present study.

### 2.1. Literature Search and Selection

**Figure 2:**
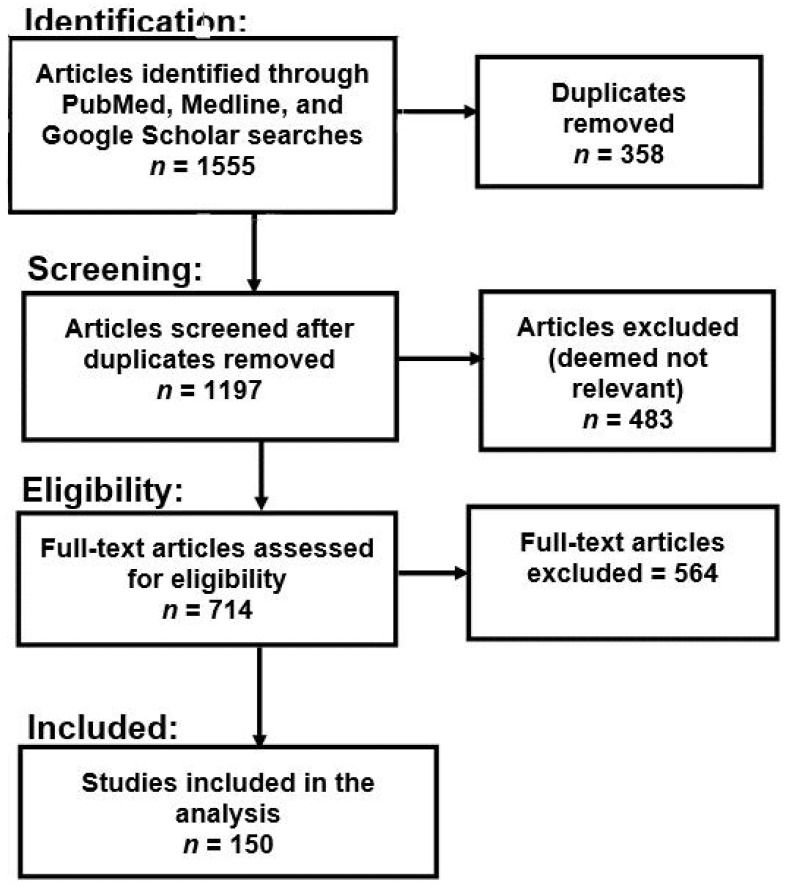
PRISMA flow diagram. In the screening process above, 1555 studies were identified, from which 358 duplicates were removed. Of the remaining 1197 studies screened by abstract, 483 were excluded as irrelevant, leaving 714 studies eligible for full-text review. Of these studies, 564 articles were excluded based on the specified exclusion criteria, and 150 were included in the final analysis.

This systematic literature review was conducted and is reported in accordance with the Preferred Reporting Items for Systematic Reviews and Meta-Analyses (PRISMA 2020) statement [39]; the completed PRISMA 2020 checklist is provided as Supplementary Material S3—Table S5, and the study identification, screening, eligibility, and inclusion process are summarized in the PRISMA flow diagram (Figure 2). This review was not registered. The process for developing the molecular systems architecture includes searching and reviewing the public scientific literature repository. CytoSolve [40] is employed to perform a systematic bioinformatics literature review, enabling screening, archiving, a distributed collaborative review, and the curation of relevant publications. The literature search and study selection process involved the following steps:

1. A list of keywords was generated (Table S1), consisting of MeSH terms such as “idiopathic pulmonary fibrosis,” “Inflammation,” “Transforming Growth Factor beta 1,” “Cellular Senescence,” “Oxidative Stress,” “Endothelial-mesenchymal transition,” “Alveolar Macrophages,” “Pericytes,” “Epithelial–mesenchymal transition, Fibroblasts,” “Autophagy,” “Apoptosis,” “Psychological stress,” “Depression,” and “Pathogenesis,” combined with the Boolean operator “AND” to improve the retrieval of relevant articles.
2. Peer-reviewed articles relevant to the topic, published from April 2008 to June 2025, were searched and retrieved from PubMed (encompassing MEDLINE) using identical MeSH keywords to form the Initial Set. Google Scholar was used only to obtain full-text versions of articles initially identified in PubMed.
3. Relevant articles were shortlisted from the Initial Set based on title and abstract screening to create the “Final Set”.
4. Full-text review was performed on the peer-reviewed articles included in the Final Set.

### 2.2. Literature Inclusion and Exclusion Criteria

The full text of the articles, not only the abstracts, was reviewed completely by the authors. Articles were deemed relevant only if the body of the text contained one or more of the keywords from Table S1 in the Supplementary Material and was directly related to the pathobiological process of IPF. The following exclusion criteria were applied during screening: the unpublished literature and abstracts (due to insufficient peer review); studies on other pulmonary diseases unrelated to IPF pathogenesis; IPF studies lacking molecular pathway details; and review articles on IPF pathogenesis published before 2015, which were excluded to avoid citing earlier reviews and to minimize redundancy with well-established pathological pathways. This date restriction applied only to review articles; primary research articles were eligible for inclusion across the full 2008–2025 search window regardless of publication date. Studies unavailable in full text or published in non-English languages were also excluded. As the present work is a mechanistic, pathway-curation systematic review rather than a quantitative outcomes meta-analysis, a formal numerical risk-of-bias scoring instrument was not applicable. Instead, two reviewers (YM and PD) independently appraised each candidate study against four pre-specified criteria: (1) a clearly stated hypothesis and objectives, (2) an appropriate experimental design, (3) transparent methods, and (4) adequate sample size. Only studies meeting these criteria were shortlisted for further review.

### 2.3. Literature Review and Data Extraction

Duplicates were removed, and two reviewers (YM and PD) independently screened the titles and abstracts of the retrieved studies against the predefined inclusion and exclusion criteria. Studies judged as potentially relevant by either reviewer were advanced to full-text review. Any disagreements regarding study eligibility or quality appraisal were resolved through discussion until consensus was reached, or by consultation with a third author (VAS) when necessary. Based on the PRISMA 2020 guidelines [40] the identification, screening, eligibility assessment, and final inclusion of studies in this systematic review are illustrated in Figure 2. During data extraction, where source studies reported conflicting mechanistic claims for the same molecular pathway—for example, differing conclusions regarding a pathway’s direction of effect or its role across model systems—the two reviewers (YM and PD) evaluated the relative weight of the evidence, considering sample size, model system (in vitro, in vivo, or human), and replication across independent studies, to determine which finding was represented in the architecture. Disagreements arising from this evidence-weighting process were resolved by consultation with a third author (VAS).

## 3. Results

The CytoSolve systematic bioinformatic analysis resulted in the molecular systems architecture of IPF pathogenesis represented by: (1) a literature review, (2) a multi-layered systems architecture schematic, (3) a systems architecture interactome of the molecular pathways, and (4) subsystems of the interactome.

### 3.1. Literature Review

The search identified 1555 records. After the removal of 358 duplicates, 1197 records were screened by title and abstract, of which 483 were excluded as irrelevant. The remaining 714 articles underwent full-text assessment; 564 were excluded per the predefined criteria, yielding 150 studies published between April 2008 and June 2025 that met the inclusion criteria and were included in the systematic analysis (Figure 2).

### 3.2. Multi-Layered Schematic of the Molecular Systems Architecture

**Figure 3:**
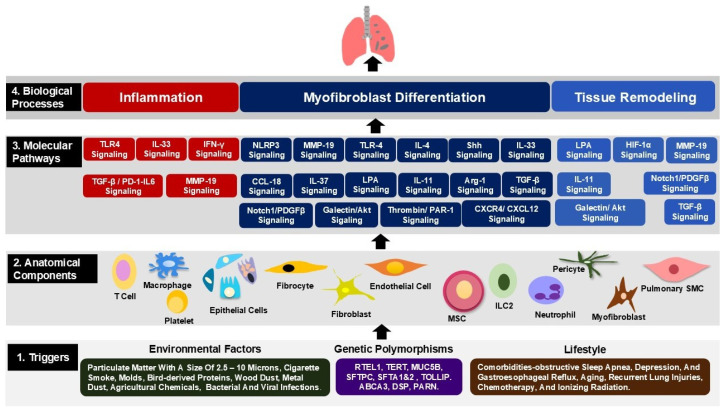
Molecular systems architecture of lung microenvironment signaling in pulmonary fibrosis, organized into four layers: Triggers, Anatomical Components, Molecular Pathways, and Biological Processes. This four-layer architecture links environmental, genetic, and lifestyle triggers to crosstalk among lung structural and immune cells, ultimately converging on inflammation, myofibroblast differentiation, and tissue remodeling. Abbreviations are defined in Supplementary Material S5 Glossary.

There are four layers to the molecular systems architecture schematic of IPF pathogenesis, as shown in Figure 3, which are described in detail below.

#### 3.2.1. The Triggers Layer

The Trigger layer consists of genetic, environmental, and lifestyle factors. The most commonly observed gene polymorphisms that contribute to IPF pathogenesis are summarized in Supplementary Material S3—Table S3. The genetic variant in the MUC5B promoter, rs35705950, has been identified as the most significant risk factor and is responsible for 30–35% of the risk of developing IPF [41]. Although short telomeres are observed in 25% of sporadic IPF patients, studies indicate that only 10% of sporadic IPF cases are attributable to pathogenic mutations in telomere maintenance pathway genes [42]. Furthermore, antigens from herpesviruses (EBV, CMV, KSHV) were detected in two of three L188Q SFTPC IPF cases, suggesting a strong link between viral infection and ER stress induced by SFTPC BRICHOS domain mutations [20,43]. Moreover, among environmental exposures, cigarette smoking is the primary risk factor for disease susceptibility in both sporadic and familial IPF [20,41].

Research studies show that IPF patients commonly have multiple comorbidities, which adversely affect quality of life and treatment response [24]. Among several comorbid conditions associated with IPF, OSA is reported as a major comorbidity, with a higher prevalence of OSA observed in 59–90% of IPF patients [44]. It has been reported that OSA causes intermittent hypoxia triggered by altered oxygen levels in the lung, which increases the risk of developing psychological disorders like depression and anxiety that exacerbate IPF [45]. This pulmonary hypoxia has been implicated in increasing pulmonary inflammation and the suppression of antifibrotic factors, contributing to the exacerbation of IPF [37]. Moreover, a cohort study including patients with IPF as the primary diagnosis has reported the presence of mood disorders like depression and anxiety in a significant proportion of IPF patients [46].

#### 3.2.2. The Anatomical Components Layer

The second layer from the bottom, as shown in Figure 3 represents cellular components of the fibrotic lung microenvironment: epithelial cells, endothelial cells, fibroblast, myofibroblast, mesenchymal stem cells, macrophages, innate lymphoid cell type 2 (ILC2), neutrophil, pulmonary smooth muscle cell, fibrocyte, pericyte, platelet, CD4^+^ T cell, CD4+ T helper (Th) cells including Th1 cell, Th2 cell, Th17 cell and T regulatory (Treg) cell. Interactions among these sixteen cell types occur across twenty-eight molecular pathways that are implicated in the lung dysfunction of biological processes leading to IPF.

#### 3.2.3. The Molecular Pathways Layer

The third layer from the bottom, as shown in Figure 3 reveals the twenty-seven molecular pathways that represent crosstalk across the fibrotic lung microenvironment. Dysfunction in these molecular pathways leads to biological processes driving IPF pathogenesis. Among these, TGF-β serves as the central profibrotic hub and modulates multiple downstream cascades that collectively regulate inflammation, myofibroblast differentiation, and tissue remodeling. These transforming growth factor beta (TGF-β)-modulated cascades include Smad signaling, programmed cell death protein 1 (PD-1)/interleukin-6 (IL-6) signaling, programmed death-ligand 1 (PD-L1) signaling, NADPH oxidase 4 (NOX4) signaling, protein kinase B (Akt) signaling, connective tissue growth factor (CTGF) signaling, sphingosine-1-phosphate (S1P) signaling, Yes-associated protein/transcriptional coactivator with PDZ-binding motif (YAP-TAZ) signaling, mechanistic target of rapamycin complex 1/activating transcription factor 4 (mTORC1-ATF4) signaling, and Smad3-p38 mitogen-activated protein kinase (Smad3-p38 MAPK) signaling. Together, these pathways integrate epithelial injury responses, fibroblast activation, extracellular matrix deposition, and progressive fibrotic remodeling within the lung microenvironment.

The remaining seventeen molecular pathways include: Toll-like receptor 4 (TLR4) signaling, interferon-γ (IFN-γ) signaling, interleukin-4 (IL-4) signaling, matrix metalloproteinase-19 (MMP-19) signaling, thrombin/protease-activated receptor-1 (thrombin/PAR-1) signaling, Notch1/platelet-derived growth factor β (Notch1/PDGFβ) signaling, C-X-C chemokine receptor type 4/C-X-C motif chemokine ligand 12 (CXCR4/CXCL12) signaling, interleukin-37 (IL-37) signaling, NLR family pyrin domain containing 3 (NLRP3) signaling, C-C motif chemokine ligand 18 (CCL-18) signaling, lysophosphatidic acid (LPA) signaling, sonic hedgehog (Shh) signaling, arginase-1 (Arg-1) signaling, interleukin-33 (IL-33) signaling, galectin/protein kinase B (galectin/Akt) signaling, hypoxia-inducible factor 1-alpha (HIF-1α) signaling, and interleukin-11 (IL-11) signaling.

#### 3.2.4. The Biological Processes Layer

The top layer, as shown in Figure 3, identifies the three biological processes of IPF pathogenesis: inflammation, myofibroblast differentiation, and tissue remodeling. Lung injury leads to inflammation, which is affected by TLR4 signaling, IL-33 signaling, IFN-γ signaling, TGF-β/PD-1-IL-6 signaling, and MMP19 signaling. The biological process of lung inflammation promotes the process of myofibroblast differentiation, that is affected by TLR4 signaling, TGF-β/Smad signaling, TGF-β/PD-1-IL-6 signaling, TGF-β/PD-L1 signaling, TGF-β/NOX4 signaling, TGF-β/Akt signaling, TGF-β/CTGF signaling, TGF-β/S1P signaling, TGF-β/YAP-TAZ signaling, and TGF-β/Smad3-p38 signaling, thrombin/PAR-1 signaling, notch signaling, CXCR4/CXCL12 signaling, IL-37 signaling, NLRP3 signaling, CCL-18 signaling, LPA signaling, Shh signaling, Arg-1 signaling, IL-33 signaling, IL-4 signaling, IL-11 signaling, galectin/Akt and MMP-19 signaling. Tissue remodeling processes, such as matrix remodeling, vascular remodeling, epithelial apoptosis, and loss of epithelial regeneration, are affected by galectin/Akt signaling, HIF-1α signaling, TGF-β/YAP-TAZ signaling, TGF-β/Smad3-p38 signaling, TGF-β/NOX4 signaling, TGF-β/mTORC1-ATF4 signaling, notch1/PDGFβ signaling, LPA signaling, MMP-19 signaling, and IL-11 signaling. The biological processes of inflammation, myofibroblast differentiation, and tissue remodeling, individually and in combination, driven by the molecular subsystems identified in the molecular pathways layer, give rise to IPF.

### 3.3. Interactome of the Molecular Systems Architecture

**Figure 4:**
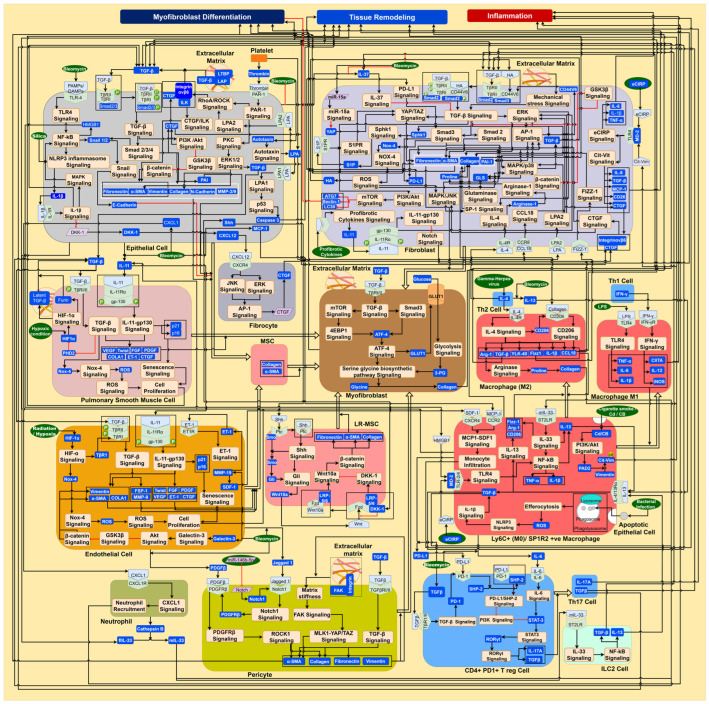
Interactome of the molecular systems architecture of the lung microenvironment in pulmonary fibrosis pathogenesis, integrating signaling across the sixteen structural and immune cell types described in the Section 3. The diagram shows how intercellular signaling networks converge on inflammation, myofibroblast differentiation, and tissue remodeling. An interactive, web-based version of this architecture, enabling cell-by-cell and pathway-by-pathway navigation, is available at https://integrativesystems.org/open-science-institute/Pulmonary-Fibrosis/web-enabled-systems-architecture (accessed on 10 July 2026). Symbol key: see Table S2, Supplementary Material S3. Abbreviations defined in Supplementary Material S5 Glossary.

Cells in the fibrotic lung microenvironment interact with each other via multiple molecular pathways. These molecular interactions—the interactome, as shown in Figure 4—occur across the epithelial cells, endothelial cells, fibroblasts, myofibroblasts, mesenchymal stem cells, macrophages, ILC2, neutrophil, pulmonary smooth muscle cells, fibrocyte, pericyte, platelet, CD4^+^ T cell, Th1 cell, Th2 cell, Th17 cell, and Treg cell. Figure 4 provides details of some of the key complex molecular interactions underlying the molecular pathways layer in Figure 3 which affect the biological processes of inflammation, myofibroblast differentiation, and tissue remodeling identified in the biological processes layer shown in Figure 3. Table S2 in the Supplementary Material S3 provides the legend for the symbols used in all interactome figures.

### 3.4. Interactome Subsystems of Molecular Systems Architecture

The details of the interactome are provided in the Supplementary Material S2, which contains the fourteen (14) subsystems that include: IL-33-signaling (Figure S1); TGF-β/PD-L1 signaling (Figure S2); TGF-β1/NOX4 signaling (Figure S3); TGF-β/CTGF signaling (Figure S4); TGF-β/S1P signaling (Figure S5); Thrombin/PAR-1 signaling (Figure S6); CXCL12/CXCR4 signaling (Figure S7); Sonic Hedgehog signaling (Figure S8); TGF-β/YAP-TAZ pathway (Figure S9); TGF-β/mTORC1/ATF4 signaling (Figure S10); TGF-β/Akt signaling (Figure S11), IL-37 signaling (Figure S12); ROS/IL-1β signaling (Figure S13); and, MMP-19 signaling (Figure S14).

### 3.5. Crosstalk Signaling Across Cellular Components of the Fibrotic Lung Microenvironment

This section is organized as follows: (1) interactive crosstalk signaling among AECII, macrophage, and fibroblast, ILC2 and immune CD4^+^ T cell; (2) interactive crosstalk signaling among macrophage, AECII, fibrocyte, fibroblast, Th17 cell, endothelial cell, and myofibroblast; (3) interactive crosstalk signaling among ECM, pulmonary smooth muscle cell, fibroblast, pericyte and myofibroblast; (4) important findings in the recent literature.

#### 3.5.1. Interactive Crosstalk Signaling Among AECII, Macrophages, Fibroblasts, ILC2, and Immune Cells

Recurrent exposure to external triggers such as cigarette smoke, microbial infection, drugs, and pollutants (silica, asbestos) triggers repeated acute inflammation, exacerbating lung epithelial damage [47]. Chronic epithelial injury caused by pathogen-associated molecular patterns (e.g., LPS) induces AECII to release damage-associated molecular patterns (DAMPs) like HMGB1, activating pattern recognition receptors (PRRs) and driving inflammation [1,48,49]. The molecular systems architecture integrates key recent findings that reveal crosstalk among multiple cell types in the fibrotic lung microenvironment. The following are the crosstalk signaling pathways across AECII, macrophages, fibroblasts, ILC2s, and immune cells.

**Figure 5:**
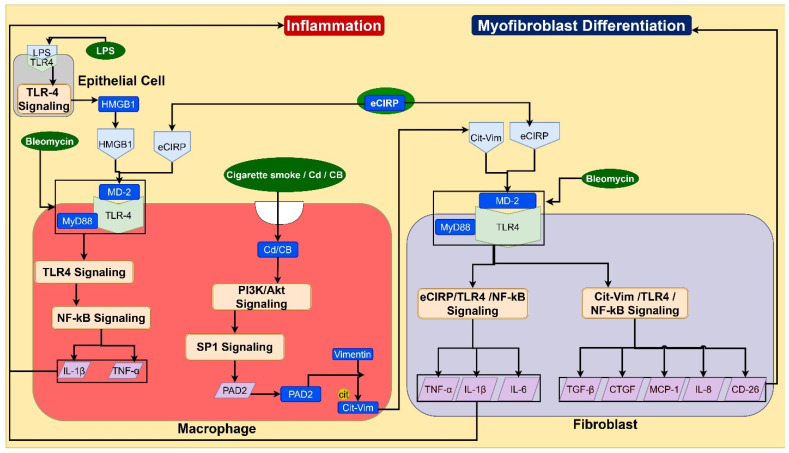
TLR4 signaling: TLR4 signaling in alveolar epithelial cells triggered by PAMPS leads to HMGB1 secretion. DAMPs like HMGB1 and eCIRP activate TLR4 signaling in macrophages and fibroblasts, contributing to lung inflammation and myofibroblast differentiation. Particulate matter (CD, CB) triggers Cit-Vim secretion by macrophages, which promotes TLR4 signaling in fibroblasts, contributing to myofibroblast differentiation. HMGB1—high-mobility group box 1; symbol key: see Table S2, Supplementary Material S3. TLR—toll-like receptor; eCIRP—extracellular cold-inducible RNA-binding protein; LPS—lipopolysaccharides; TNFα—tumor necrosis factor alpha; Cd—cadmium; CB—carbon black; SP1—specificity proteins; PAD2—peptidyl arginine deiminase 2; Cit-Vim—citrullinated vimentin; MCP-1—monocyte chemoattractant protein-1; connective tissue growth factor (CTGF); PI3K—phosphoinositide 3-kinase; IL—interleukin; CD26—cluster of differentiation 26. Symbol key: see Table S2, Supplementary Material S3. Abbreviations defined in Supplementary Material S5 Glossary.

Studies demonstrate that TLR4 signaling induces lung inflammation, promoting the development of IPF [1,50]. Clinically, a high level of TLR4 expression was observed in lung biopsies and serum samples from IPF patients [51]. DAMPs, including heat shock protein alpha B-crystallin (HSPB5), S100A4, fibronectin, and tenascin, act as potent TLR4 agonists linked to fibrosis [1]. Reports indicate TOLLIP polymorphisms (TLR4 adaptor protein) strongly associate with IPF risk [51]. A high level of HMGB1 expression in alveolar macrophages and AECII activates TLR4-MyD88/NF-κB, driving IL-1β/TNF-α production and fibrosis progression, as shown in Figure 5 [52,53].

Recent evidence shows DAMPs, extracellular cold-inducible RNA-binding protein (eCIRP), and citrullinated vimentin (Cit-Vim) induce profibrotic and inflammatory fibroblast phenotypes, respectively [54,55]. Cigarette smoke-derived cadmium (Cd), carbon black (CB), and particulate matter (<2.5 μm) are phagocytosed by alveolar macrophages, upregulating peptidyl arginine deiminase 2 (PAD2) via PI3K/Akt-1/SP-1 signaling to produce Cit-Vim, as illustrated in Figure 5 [54,56]. Cit-Vim acts as a DAMP, activating TLR-4-myeloid differentiation factor 2 (MD-2) receptor complex on fibroblasts, driving NF-κB-mediated upregulation of CD26 and profibrotic cytokines such as TGF-β1, CTGF, monocyte chemoattractant protein-1 (MCP-1), and IL-8 [54]. CD26+ fibroblasts exhibit high proliferation and invasiveness, differentiating into myofibroblasts that secrete elevated collagen and alpha-smooth muscle actin (α-SMA) levels, implicating Cit-Vim in IPF pathogenesis [54]. PAD2 inhibition offers therapeutic potential against Cd/CB- or smoke-induced fibrosis [54].

Bolourani et al. (2021) identified eCIRP as a novel DAMP promoting BLM-induced IPF via TLR4-dependent activation of macrophages and fibroblasts [55]. eCIRP induces inflammatory phenotypes in alveolar macrophages and lung fibroblasts, upregulating proinflammatory cytokines (TNF-α, IL-1β, IL-6) as illustrated in Figure 5. These cytokines persistently activate fibroblasts through paracrine/autocrine signaling, accelerating fibrosis progression [56].

**Figure 6:**
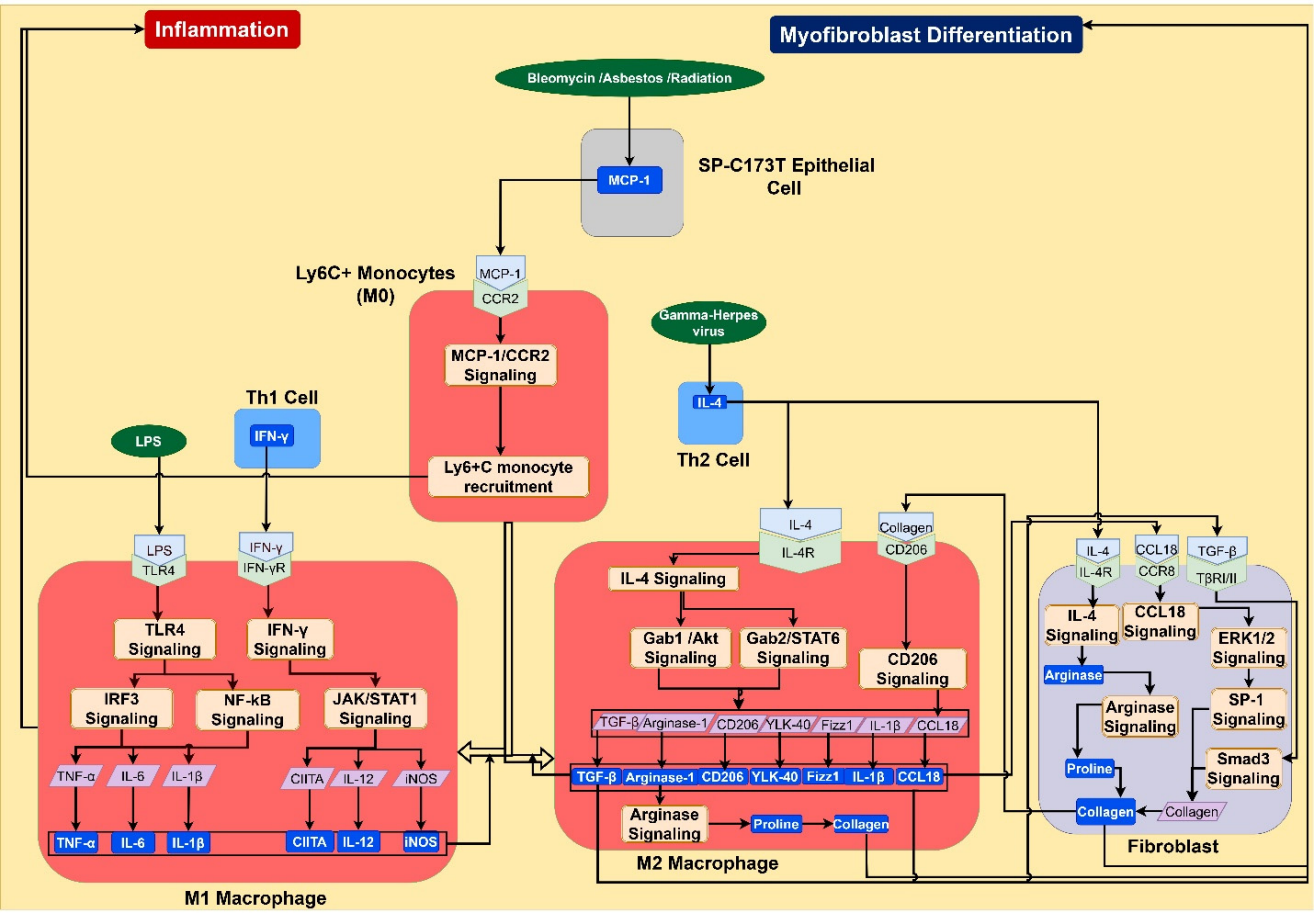
IFN-γ and IL-4 signaling in monocytes. IFN-γ and IL-4 signaling in LY6+ monocytes in Th1 and Th2 microenvironments stimulate the polarization of monocytes into M1 and M2 macrophages, contributing to inflammation and myofibroblast differentiation, respectively. MCP-1 signaling promotes the recruitment of Ly6+ monocytes into the lung microenvironment. The Arg-1 signaling in M2 macrophages promotes increased collagen production. IL-4, CCL18, and TGF-β signaling in fibroblasts augments collagen production, which subsequently activates CD206 signaling in M2 macrophages, increasing CCL18 production in a feedback loop manner. This contributes to persistent M2 macrophage activation and amplification of myofibroblast differentiation. Symbol key: see Table S2, Supplementary Material S3. Abbreviations defined in Supplementary Material S5 Glossary.

Alveolar macrophages (AMs) are the primary resident innate immune cells in lung alveoli [57] and drive IPF by promoting fibroproliferation and collagen synthesis after tissue damage [58]. Their polarization into pro-inflammatory M1 or profibrotic M2 phenotypes is central to fibrosis progression [57]. Genetic mutations, such as in surfactant protein C (SP-C I73T), trigger acute inflammatory exacerbations (AIE) by increasing lymphocyte antigen 6 (Ly6C^+^) monocyte recruitment via the CCL2/CCR2 axis following epithelial injury from BLM, radiation, or asbestos [59,60]. Damaged epithelial cells harboring the mutant surfactant protein (SP-C I73T) secrete CCL2, recruiting CCR2^+^ Ly6C+ monocytes to sites of lung injury [60]. These monocytes promote M1 polarization through IFN-γ/JAK/STAT1 and LPS/TLR4/NF-κB/IRF3 pathways, inducing the secretion of inflammatory mediators such as CIITA, iNOS, IL-12, TNF-α, and IL-6, driving AIE, as illustrated in Figure 6 [57,60,61,62].

Zhang et al. (2018) demonstrated that Th2 cytokine IL-4 primarily induces M2 macrophage differentiation via JAK1/STAT6 and PI3K/Akt signaling in a BLM-induced lung fibrosis model [62]. Gab1 and Gab2 mediate IL-4 signaling by activating Akt and STAT6, respectively, upregulating M2 markers including TGF-β, Arg-1, Fizz1/RELM-α, YKL-40, CCL18, IL-1β, and macrophage mannose receptor (CD206), which promote fibroblast activation and ECM deposition [57,62,63,64]. Additionally, Arg-1 signaling and CCL-18 signaling, as seen in Figure 6 and the IL-33-IL-13 signaling axis and as illustrated in Supplementary Material S2—Figure S1, further promote macrophage M2 polarization in fibrotic lung, exacerbating fibrosis. Mechanistic details of these signaling pathways are discussed in detail in Supplementary Material S1.

**Figure 7:**
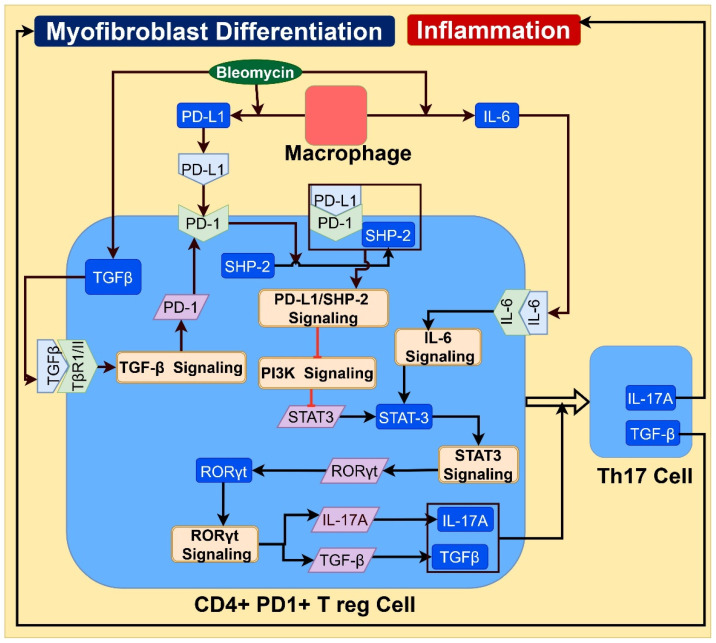
TGF-β/PD-1-IL-6 signaling. TGF-β/PD-1-IL-6 signaling in CD4^+^ PD-1^+^ Treg cells promotes the differentiation of Treg cells to Th17 cells, contributing to inflammation and myofibroblast differentiation. Symbol key: see Table S2, Supplementary Material S3. Abbreviations defined in Supplementary Material S5 Glossary.

Evidence shows an association between PD-1 receptor overexpression on BLM-stimulated CD4^+^ T cells and IPF [65,66]. TGF-β1 upregulates PD-1 via Smad3 activation [65,67]. Findings suggest that such PD-1^+^ CD4^+^ T cells derive from Tregs and, when activated by IL-6, differentiate into Th17 cells via IL-6/STAT3 signaling, producing TGF-β and IL-17A [65,66]. PD-1/programmed death ligand 1 (PD-L1) binding recruits SHP-2, which dephosphorylates PI3K, thereby activating STAT3, promoting RORγt expression to drive Th17 differentiation, as shown in Figure 7 [65]. Gancao Ganjiang decoction (GGD) targets the PD-1/STAT3/TGF-β/IL-17A axis, reducing PD-1 and PD-1^+^ CD4^+^ T cells in BLM-induced fibrotic lung tissue [66].

IL-37, an anti-inflammatory cytokine expressed by alveolar macrophages and AECs, suppresses immuno-inflammatory responses. Immunohistochemistry studies revealed decreased IL-37 expression in lung tissue from IPF patients and BLM-treated animals [68]. Kim et al. [68] demonstrated that IL-37 inhibits oxidative stress-induced AEC death and suppresses TGF-β signaling in IPF fibroblasts by downregulating TβRII phosphorylation, reducing Smad2/3, ERK, p38MAPK, and PI3K-Akt activation, and decreasing fibronectin, α-SMA, and collagen I expression. IL-37 also inhibits mTOR, enhancing autophagy markers (LC3II, beclin-1, ATG7) and autophagic flux in IPF fibroblasts. Thus, IL-37 therapy inhibits IPF fibroblast proliferation and differentiation, positioning it as a promising therapeutic target for IPF [29,68]. Comprehensive details of IL-37 signaling are discussed in Supplementary Material S1 and schematically represented in Supplementary Material S2—Figure S12.

#### 3.5.2. Interactive Crosstalk Signaling Among Macrophage, AECII, Fibrocyte, Fibroblast, Th17 Cell, Endothelial Cell, and Myofibroblast

Persistent lung–environmental crosstalk causes alveolar epithelial microinjuries, driving phenotypical changes in lung structural cells that trigger profibrotic cascades, including epithelial (AECII)–mesenchymal transition (EMT), endothelial–mesenchymal transition (EndMT), and fibroblast–myofibroblast transition (FMT) [21,48]. Both EMT and EndMT drive myofibroblast differentiation by converting epithelial or endothelial cells into a mesenchymal phenotype, where these transitioned cells express myofibroblast markers (e.g., α-SMA) [23,32]. Major signaling pathways that mediate myofibroblast differentiation, including TGF-β signaling pathways, CXCL12 signaling, and NLRP3 signaling, are discussed in detail below.

**Figure 8:**
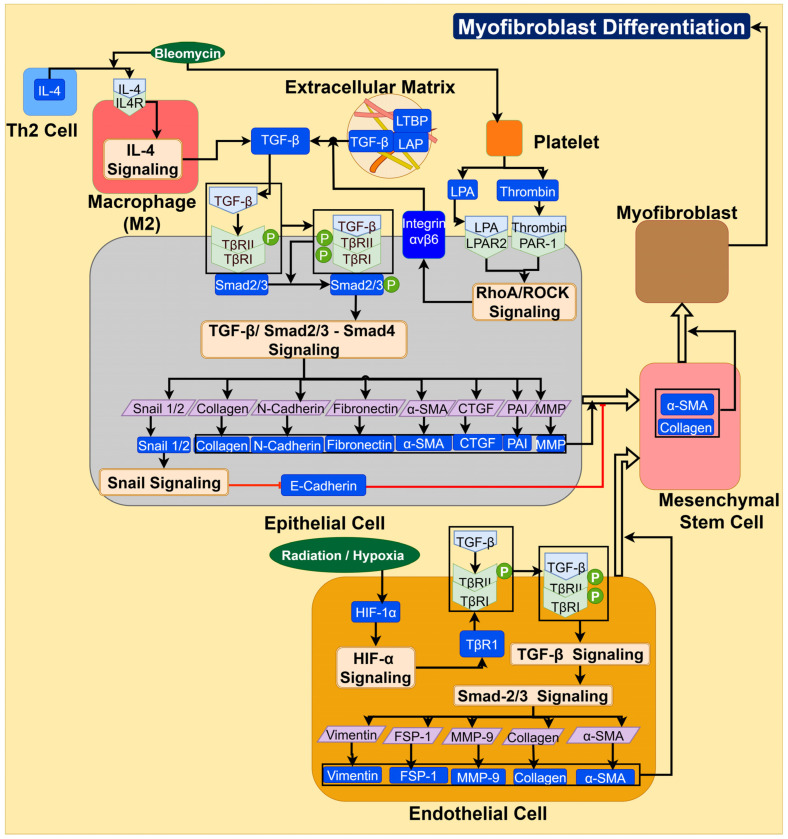
TGF-β1/Smad signaling: TGF-β1/Smad signaling promotes epithelial and endothelial cell differentiation into mesenchymal cells via EMT and EndMT, contributing to myofibroblast differentiation. Symbol key: see Table S2, Supplementary Material S3. Abbreviations defined in Supplementary Material S5 Glossary.

TGF-β, a major profibrotic cytokine in IPF, is secreted by alveolar macrophages, activated AECII, endothelial cells, resident fibroblasts, and myofibroblasts [20,69]. TGF-β1 signaling drives lung fibroblast proliferation and differentiation into myofibroblasts by promoting precursor cell transdifferentiation via EMT, FMT, and EndMT in a proinflammatory microenvironment enriched with polarized macrophages, thereby establishing a profibrotic milieu in IPF [22,69]. Thrombin and LPA, released from platelets after injury, activate epithelial PAR1 and LPA2, respectively. This triggers Gαq–RhoA–Rho kinase signaling, inducing cytoskeletal contraction and the αvβ6 integrin-mediated release of active TGF-β from an ECM-bound latency-associated peptide (LAP)-latent TGF-β-binding protein (LTBP) complexes, as seen in Figure 8 [70]. Moreover, as shown in the Supplementary Material S2—Figure S6, thrombin also promotes EMT through PAR-1/PKC/ERK1/2 signaling in AECII, upregulating the expression of collagen I, TGF-β1, and α-SMA to drive myofibroblast differentiation [71]. Details of the signaling mechanism are provided in Supplementary Material S1.

Active TGF-β binds TβRII, recruiting and phosphorylating TβRI to activate canonical Smad-dependent and noncanonical pathways [72]. TβRI phosphorylates Smad2/3, which complexes with Smad4 and translocates to the nucleus, inducing SNAIL1/2 repressors that downregulate E-cadherin while upregulating mesenchymal proteins such as N-cadherin, fibronectin, α-SMA, collagen, CTGF, plasminogen activator inhibitor (PAI), and MMPs. This drives EMT and myofibroblast differentiation, as schematically represented in Figure 8 [31,69,73,74]. In radiation-induced IPF models, hypoxic damage to vascular endothelial cells promotes EndMT [75]. Hypoxia in irradiated human pulmonary artery endothelial cells (HPAEC) upregulates HIF-1α, enhancing TβRI expression and TGF-β/Smad2/3 signaling. This induces fibroblast markers (vimentin, FSP1, α-SMA, MMP-9, collagen), driving mesenchymal transition and myofibroblast differentiation, as illustrated in Figure 8 [74].

The remaining TGF-β-modulated cascades driving EMT and myofibroblast differentiation include TGF-β/PD-L1, TGF-β/NOX4, TGF-β/Akt, TGF-β/CTGF, and TGF-β/S1P signaling, and are schematically represented in Supplementary Material S2 (Figures S2–S5 and S11), with full mechanistic detail provided in Supplementary Material S1.

The CXCL12/CXCR4 signaling axis has been reported as a key mechanism for recruiting fibrocytes in BLM-induced IPF pathogenesis. CXCL12 recruits CXCR4+ bone marrow-derived fibrocytes to the lungs, where they differentiate into collagen-producing fibroblasts [76,77,78]. Fibrocytes comprise ~20% of IPF myofibroblasts, with elevated levels in blood and BALF signaling disease exacerbation [79]. Comprehensive details of the CXCL12/CXCR4 signaling pathway are discussed in Supplementary Material S1 and schematically represented in Supplementary Material S2—Figure S7.

**Figure 9:**
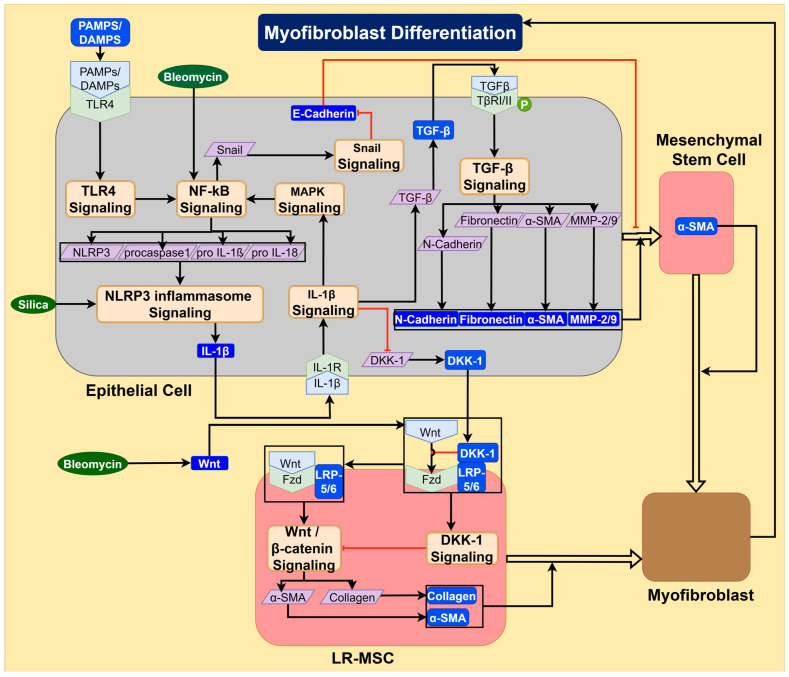
NLRP3 signaling: NLRP3 inflammasome signaling in AECII promotes the EMT process leading to myofibroblast differentiation. Inflammasome-derived IL-1β downregulates the expression of the WNT antagonist DKK-1 in epithelial cells and promotes WNT signaling in lung-resident LR-MSCs, facilitating their differentiation into myofibroblasts. Symbol key: see Table S2, Supplementary Material S3. Abbreviations defined in Supplementary Material S5 Glossary.

NLRP3 inflammasome signaling is well-studied for its inflammatory role in IPF pathogenesis, including silicosis [28]. The NLRP3 inflammasome multiprotein complex comprises the sensor protein NLRP3, an adaptor protein apoptosis-associated speck-like protein containing a CARD (ASC), and effector pro-caspase 1 [80]. Studies indicate elevated NLRP3 inflammasome activation in lung tissue from both IPF patients and IPF mice [81]. The NLRP3 signaling mechanism is elaborately discussed in Supplementary Material S1 and illustrated in Figure 9. NLRP3 inflammasome activation occurs in IPF models induced by asbestos, silica, and BLM [80,82,83]. In BLM models, NLRP3 regulates epithelial–mesenchymal transition (EMT) by promoting NF-κB-mediated expression of EMT markers (fibronectin, α-SMA, vimentin, N-cadherin, MMP-2/9) [81,84]. Tian et al. (2017) reported that NLRP3-driven IL-1β expression and signaling in AECII enhance TGF-β1 production, thereby suppressing E-cadherin to drive EMT [83]. While IL-1β/NF-κB feedback sustains NLRP3 overexpression and inflammation, as illustrated in Figure 9 [80].

Jäger et al. (2021) found that bronchoalveolar lavage (BAL) cells from IPF patients with acute exacerbation (AE) show hyper-inducible NLRP3 inflammasome activity, releasing more IL-1β than healthy controls [84]. This NLRP3 hyperactivation is likely driven by reactive oxygen species (ROS) generated during macrophage efferocytosis of apoptotic epithelial cells, which enhances NLRP3 expression and caspase-1-dependent IL-1β production, as illustrated in Supplementary Material S2—Figure S13 [84]. The resulting IL-1β further amplifies lung fibrosis by stimulating TGF-β-mediated fibroblast activation [84].

Comprehensive details of the silica-induced EMT process mediated by NLRP3/IL-1β signaling, which suppresses dickkopf-1 (DKK1) secretion by alveolar epithelial type II cells (AECII) to promote Wnt signaling in lung-resident mesenchymal stem cells (LR-MSCs) and drive their differentiation into myofibroblasts, are presented in Supplementary Material S1 and schematically illustrated in Figure 9. Additionally, the mechanism by which Shh/Wnt10 signaling in LR-MSCs promotes their differentiation into myofibroblasts is depicted in Supplementary Material S2—Figure S8 and described in detail in Supplementary Material S1.

#### 3.5.3. Interactive Crosstalk Signaling Among Epithelial Cells, Endothelial Cells, the ECM, Pulmonary Smooth Muscle Cells, Fibroblasts, Pericytes, and Myofibroblasts

Myofibroblasts from EMT, FMT, and EndMT promote ECM accumulation in IPF. This drives alveolar destruction, tissue remodeling, and increased lung stiffness [85]. EndMT specifically fuels pulmonary vascular remodeling by impairing endothelial integrity and elevating vascular permeability [86,87]. Moreover, myofibroblasts undergo metabolic reprogramming, with elevated glycolysis and glutaminolysis fueling the biosynthesis of ECM proteins like collagen [88]. Alterations in glycolysis, glutamine, and lipid metabolism were reported in human lungs with severe IPF [89]. Key signaling pathways implicated in lung tissue remodeling in pulmonary fibrosis, including LPA signaling, galectin/Akt signaling, HIF-1α/NOX4 signaling, Notch1/PDGFβ signaling, and TGF-β/Smad3-p38MAPK signaling, are discussed below.

**Figure 10:**
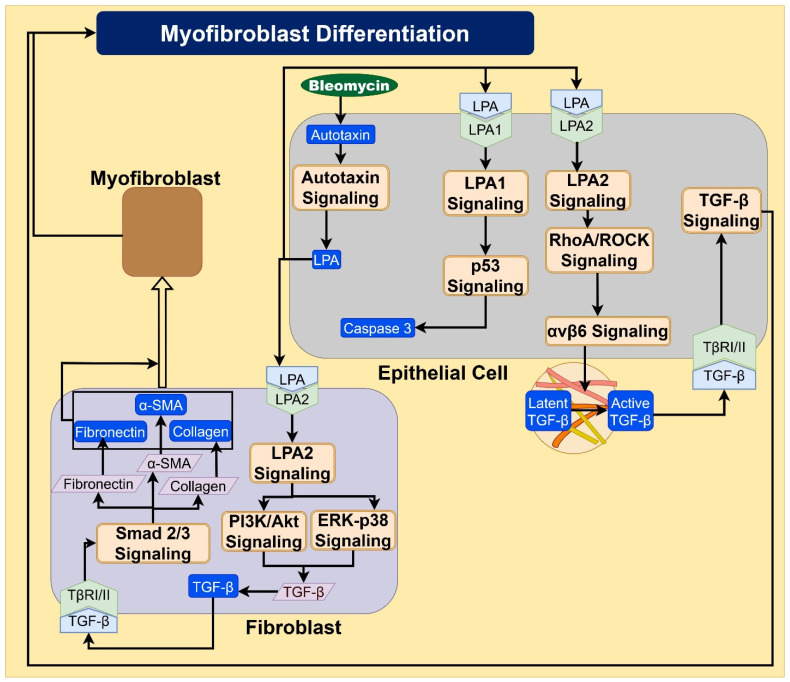
LPA signaling: LPA/LPA1 signaling in epithelial cells contributes to apoptosis, contributing to tissue remodeling, and LPA/LPA2 signaling in both epithelial cells and fibroblasts promotes EMT and FMT, respectively, contributing to myofibroblast differentiation. Symbol key: see Table S2, Supplementary Material S3. Abbreviations defined in Supplementary Material S5 Glossary.

The lysophosphatidic acid (LPA) axis has emerged as a key contributor to IPF pathogenesis and a promising therapeutic target. Autotaxin (ATX), a lysophospholipase D, generates LPA from lysophosphatidylcholine (LPC), which is produced from membrane phosphatidylcholine (PC) by phospholipase A1/2 [90,91]. Studies report ATX overexpression in alveolar macrophages, fibroblastic foci, and alveolar epithelium of lung tissue from IPF patients, as well as in bronchoalveolar lavage fluid (BALF) from BLM-induced IPF models [90]. Elevated LPA enhances fibroblast chemotaxis, with LPA1 and LPA2 receptors predominantly expressed in IPF lung tissue [90,92,93]. BLM-induced activation of LPA–LPA1 signaling in alveolar epithelial cells upregulates p53, p21, and caspase-3, driving AECII apoptosis and lung tissue remodeling, as illustrated in Figure 10 [92]. Additional details of LPA-LPA2 signaling in AECII and fibroblast, which induces myofibroblast differentiation, are provided in Supplementary Material S1 and schematically represented in Figure 10.

**Figure 11:**
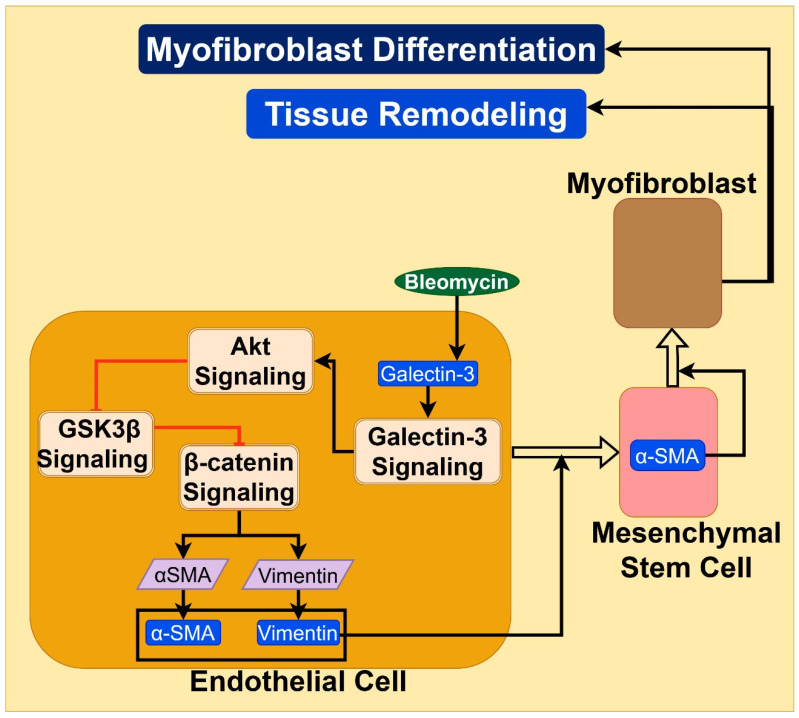
Galectin/Akt signaling: Galectin/Akt signaling in endothelial cells promotes the differentiation of endothelial cells to mesenchymal stem cells through the process of EndMT, leading to myofibroblast differentiation, and contributing to vascular remodeling. Symbol key: see Table S2, Supplementary Material S3. Abbreviations defined in Supplementary Material S5 Glossary.

Single-cell RNA sequencing of Tie+ endothelial cells from BLM-induced fibrotic lungs identified progressive galectin-3 upregulation during endothelial-to-fibroblast transition, with galectin-3 activating the Akt/GSK-3β/β–catenin pathway in human pulmonary microvascular endothelial cells (HPMEC) [94]. As depicted in Figure 11, this galectin/Akt signaling drives α-SMA expression, myofibroblast proliferation, and vascular remodeling [94].

**Figure 12:**
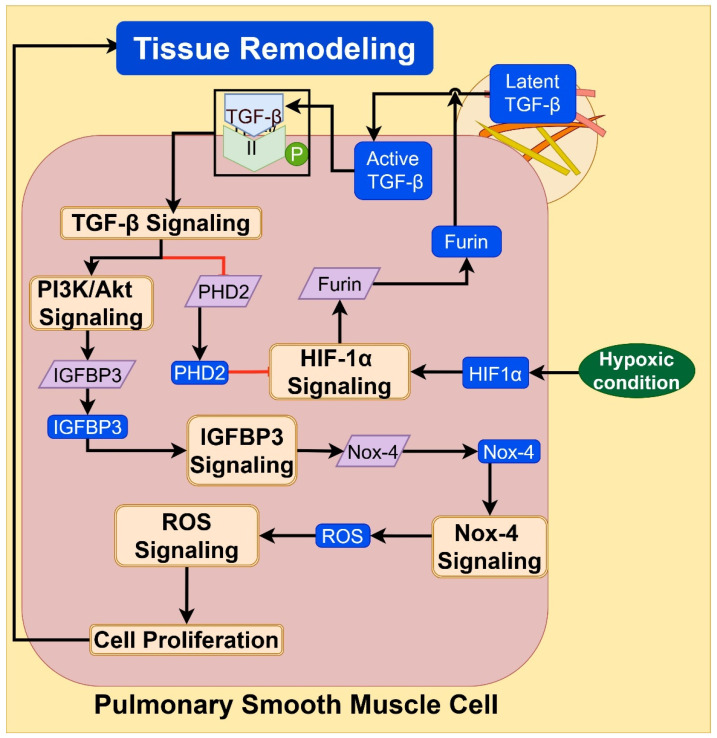
HIF-1α/NOX4 signaling: HIF-1α/NOX4 signaling in pulmonary artery smooth muscle cells promotes cell proliferation, contributing to vascular tissue remodeling. Symbol key: see Table S2, Supplementary Material S3. Abbreviations defined in Supplementary Material S5 Glossary.

Under hypoxic conditions, TGF-β1/NOX4 signaling exacerbates IPF pathogenesis by driving human pulmonary arterial smooth muscle cell (HPASMC) proliferation, vascular remodeling, and secondary pulmonary hypertension (PH) [95,96]. Hypoxia-inducible factor-1 (HIF-1α) mediates furin expression, enabling proteolytic activation of TGF-β1 from pro-TGF-β1 [96]. TGF-β1 signaling downregulates prolyl hydroxylase domain 2 (PHD2), stabilizing HIF-1α and amplifying activated TGF-β1 production [95,96]. Autocrine TGF-β1 further activates PI3K/Akt signaling to upregulate insulin-like growth factor binding protein 3 (IGFBP-3), which transactivates NOX4 expression, generating ROS that promote HPASMC proliferation and vascular tissue remodeling, as depicted in Figure 12 [95,96].

**Figure 13:**
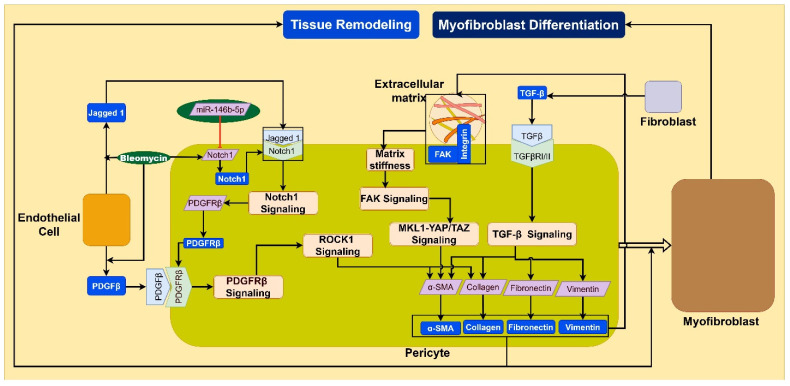
Notch1/PDGFβ—TGF-β/YAP-TAZ-MKL-1 signaling axis: Notch1/PDGFβ signaling in pericytes promotes myofibroblast differentiation, contributing to matrix tissue remodeling. In pericytes, the TGF-β signaling promotes myofibroblast differentiation, leading to matrix tissue remodeling, which further activates YAP-TAZ-MKL-1 signaling, contributing to increased myofibroblast production. Symbol key: see Table S2, Supplementary Material S3. Abbreviations defined in Supplementary Material S5 Glossary.

Experimental studies demonstrate that pericyte-myofibroblast transition (PMT) drives myofibroblast expansion and disease severity [97]. Pathologically, PMT involves pericyte detachment from endothelial walls, migration into the interstitial space, and differentiation into myofibroblasts [97]. In human fibrotic lungs and BLM animal models, Jagged1/Notch1 signaling is upregulated, promoting PMT via increased expression of Notch1, platelet-derived growth factor receptor β (PDGFRβ), and Rho-associated protein kinase 1 (ROCK1) [98,99]. Endothelial-derived Jagged1 activates Notch signaling in pericytes, upregulating PDGFRβ expression [97]. Vascular endothelial cells release platelet-derived growth factor β (PDGFβ), recruiting pericytes via PDGFRβ and activating the PDGFβ/PDGFRβ/ROCK1 signaling cascade, as illustrated in Figure 13. This promotes pericyte proliferation and myofibroblast differentiation marked by reduced desmin and NG2, while elevating α-SMA and collagen I, to drive ECM remodeling [97,99,100]. Moreover, studies report diminished miR-146b-5p expression in IPF lung fibroblasts, positioning it as a promising therapeutic target. It directly targets Notch1, suppressing the Notch1/PDGFRβ/ROCK1 pathway in lung pericytes and mitigating fibrosis, as schematically represented in Figure 13 [98].

Additionally, resident fibroblast-derived TGF-β1 promotes pericyte-to-myofibroblast differentiation, inducing collagen, fibronectin, α-SMA, and vimentin expression that drives ECM accumulation and tissue stiffening [85]. Matrix stiffening then activates mechanotransduction pathways, promoting megakaryoblastic leukemia 1 (MKL-1) nuclear translocation to enhance profibrotic gene expression and α-SMA+ myofibroblast accumulation [85]. The details of the TGF-β/YAP-TAZ-MKL-1 signaling mechanism are provided in Supplementary Material S1 and illustrated in Figure 13.

**Figure 14:**
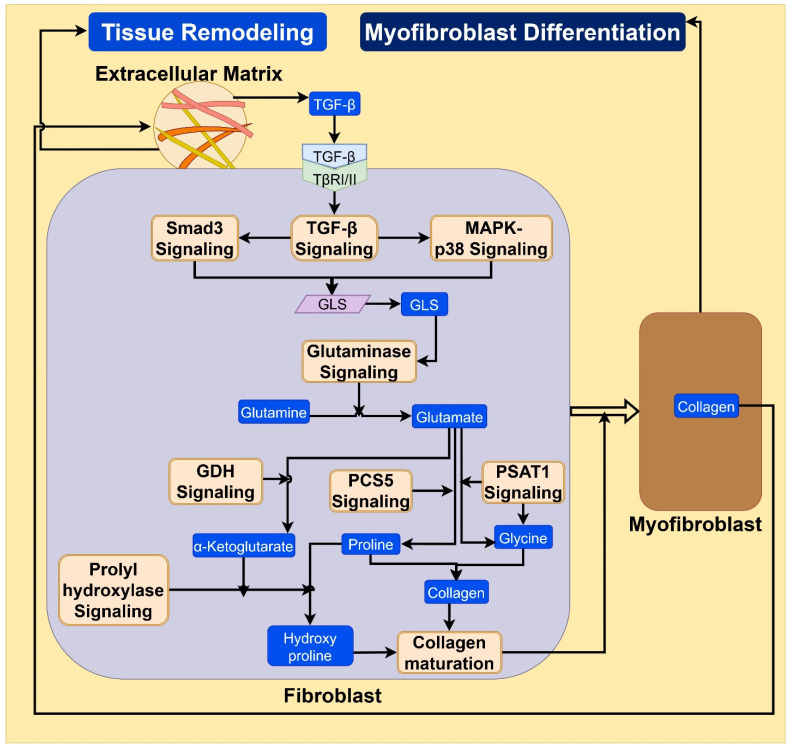
TGF-β/Smad3-p38MAPK signaling axis: TGF-β/Smad3-p38MAPK signaling axis in fibroblasts promotes glutaminolysis, leading to the increased production of ECM protein collagen, contributing to matrix tissue remodeling. Symbol key: see Table S2, Supplementary Material S3. Abbreviations defined in Supplementary Material S5 Glossary.

In myofibroblasts, glutamine undergoes glutaminolysis to produce glutamate, a key precursor for proline and collagen synthesis. It has been demonstrated that TGF-β1 signaling via Smad3 and p38-MAPK pathways upregulates glutaminase expression, thereby enhancing collagen production [85,101,102]. Glutaminase (GLS) mediates the conversion of glutamine to glutamate, which is further metabolized by glutamate dehydrogenase (GDH) to α-ketoglutarate (α-KG) [102,103]. As shown in Figure 14, α-ketoglutarate, as a substrate for proline hydroxylases, facilitates the hydroxylation of proline residues of collagen, which promotes collagen maturation and matrix production in IPF [104]. Moreover, glutamate is also metabolized by enzymes pyrroline-5-carboxylate synthase (P5CS) and phosphoserine aminotransferase (PSAT1) to produce proline and glycine, respectively, in differentiated myofibroblasts, as seen in Figure 14. This augments collagen accumulation and the progression of IPF [104].

Additional TGF-β-modulated cascades contributing to matrix remodeling, TGF-β/YAP-TAZ and TGF-β/mTORC1-ATF4 signaling, are schematically represented in Supplementary Material S2 (Figures S9 and S10), with full mechanistic detail in Supplementary Material S1.

#### 3.5.4. Important Findings in the Recent Literature

**Figure 15:**
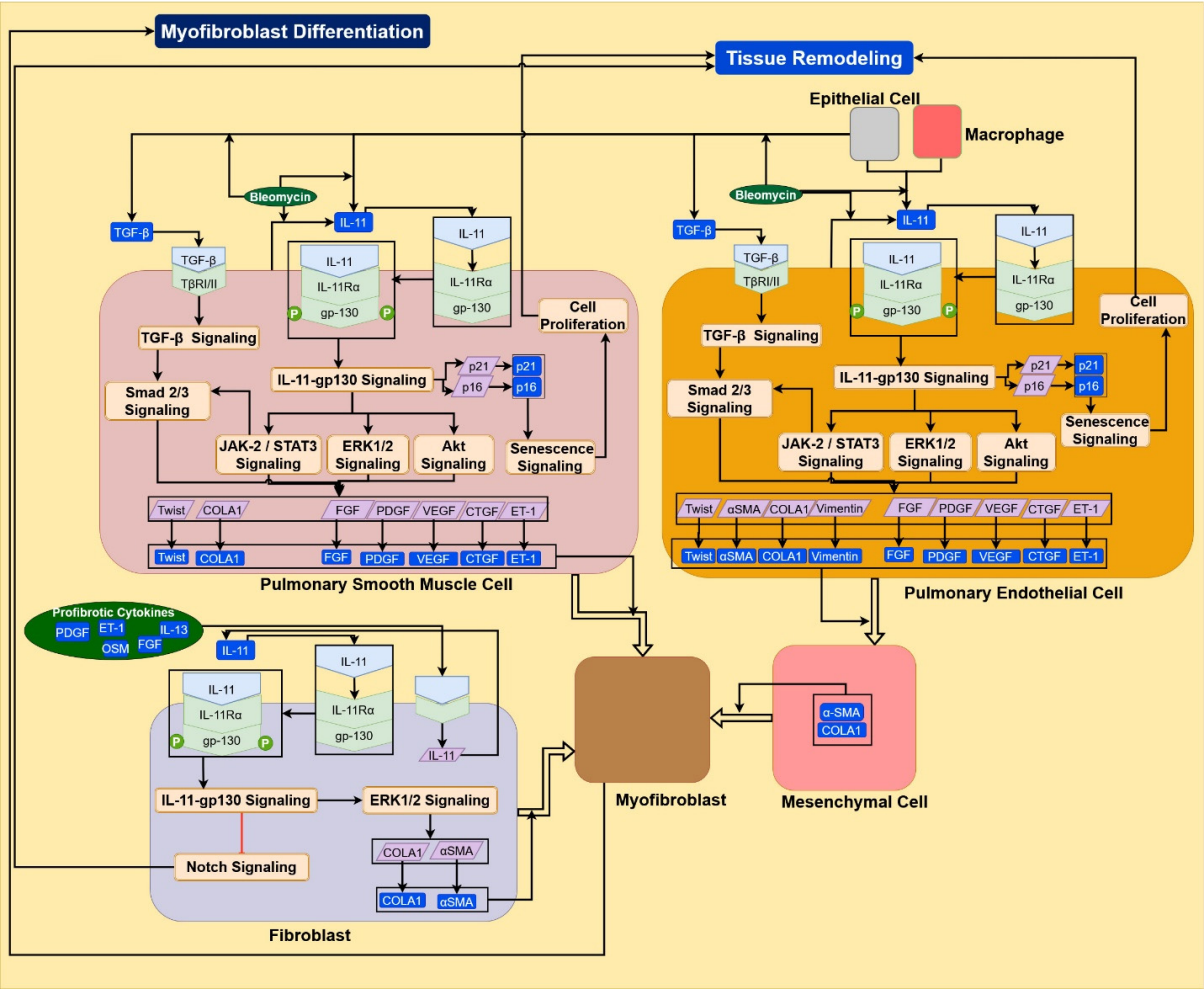
IL-11 signaling: IL-11 signaling in pulmonary smooth muscle cells promotes myofibroblast differentiation and cell proliferation, leading to vascular tissue remodeling. IL-11 signaling in endothelial cells promotes the EndMT process and activates fibroblast transition to myofibroblasts, contributing to myofibroblast differentiation. In fibroblasts, the IL-11 signaling inhibits notch crosstalk signaling with epithelial progenitor cells, contributing to the impairment of epithelial regeneration. Symbol key: see Table S2, Supplementary Material S3. Abbreviations defined in Supplementary Material S5 Glossary.

The Role of IL-11 Crosstalk Signaling Among Endothelial Cells, Pulmonary Smooth Muscle Cells, Mesenchymal Cells, Fibroblasts, and Myofibroblasts: A significant increase in the expression of IL-11 and IL-11Rα in pulmonary fibroblasts, as well as pulmonary artery tissue, which comprises endothelial and pulmonary smooth muscle cells, is found among patients with IPF and IPF-associated pulmonary hypertension (IPF-PH) [105,106,107]. Several profibrotic cytokines, such as TGF-β1, PDGF, FGF2, IL-13, Oncostatin M (OSM), and endothelin 1 (ET-1), induce the expression of IL-11 in lung fibroblasts, which in turn activates the ERK pathway to enhance collagen and α-SMA production, as shown in Figure 15 [106].

IL-11 mediates pulmonary artery remodeling and hypertension by promoting the transition of human pulmonary artery endothelial cells (HPAEC) and pulmonary smooth muscle cells (HPASMC) into the myofibroblast phenotype [105]. The EndMT process has been shown to increase the levels of contractile and ECM proteins (Twist, collagen type 1, α-SMA, vimentin), growth factors (FGF, PDGF, CTGF, VEGF), and the vasoconstrictor protein ET-1, while causing a significant reduction in endothelial markers such as CD31, VE-cadherin, and FVIII in mesenchymal cells [105]. Furthermore, IL-11 also facilitated the transition of HPASMC into myofibroblasts by elevating the expression of TWIST-1, collagen type I, FGF, PDGF, CTGF, VEGF, and ET-1, while downregulating the contractile protein α-SMA, as seen in Figure 15 [105].

Milara et al. (2022) also showed higher expression of senescent markers, including p21 and p16, that significantly correlated with IL-11 overexpression in the IPF-pulmonary arteries [105]. Augmentation of senescent endothelial and pulmonary smooth muscle cells in the IPF pulmonary arteries is accompanied by SASP mediators, which promote the hyperplasia and migration of non-senescent pulmonary smooth muscle cells, contributing to vascular remodeling and consequent pulmonary hypertension [105].

Furthermore, Kortekaas et al. (2023) identified IL-11 as a key factor that impairs alveolar epithelial regeneration in IPF by inhibiting notch signaling in lung fibroblasts [107]. Notch signaling is increased in normal lung fibroblasts, which regulate progenitor cell activation to promote cell differentiation [107]. This IL-11-mediated suppression of notch signaling disrupts the crosstalk between fibroblast and epithelial progenitor cells, and prevents type 1 alveolar epithelial cell regeneration [107]. The details of the IL-11/IL-11Rα signal transduction pathway are discussed in Supplementary Material S1 and schematically represented in Figure 15.

Role of MMP-19 Crosstalk Signaling Between Endothelial Cells, Macrophages, Mesenchymal Cells, and Myofibroblasts: Elevated MMP-19 expression has been observed in the endothelial cells of both IPF patients and BLM-induced fibrotic mouse lungs. Zhao et al. [87] reported that MMP-19 promotes the EndMT in BLM-exposed murine lungs and human HPMEC via an endothelin-1 (ET-1)-dependent pathway. This EndMT is marked by increased vimentin and α-SMA and decreased CD31 and VE-cadherin, with ET-1 as a key marker of endothelial dysfunction, as illustrated in Supplementary Material S2—Figure S14 [87]. MMP-19 upregulates endothelial ET-1, which, through its receptor, drives EndMT, enhances vascular permeability, and increases collagen secretion by endothelial-derived fibroblasts, contributing to vascular tissue remodeling [87]. Additionally, MMP-19 induces endothelial stromal cell-derived factor 1 (SDF1) expression, which interacts with CXCR4 on monocytes, promoting their adhesion and infiltration into lung tissue, further supporting a fibrotic microenvironment, as depicted in Supplementary Material S2—Figure S14 [87].

Comorbidities Exacerbate IPF: Reports indicate that patients with IPF are also affected by OSA, which is characterized by poor sleep quality and sleep-disordered breathing [45]. This OSA causes intermittent hypoxia, due to repetitive cycles of decreased blood oxygen levels (hypoxia) followed by reoxygenation during the period of breathing, which promotes psychological disorders like depression and anxiety in animal models of BLM-induced IPF [45].

Xiong et al. [45] demonstrated that in BLM-induced mice, elevated pulmonary inflammatory cytokines, including TNFα and IL-6, correlate with hippocampal microglial activation, leading to neuroinflammation. Exposure to intermittent hypoxia exacerbated the inflammatory response in BLM-exposed mice, increasing TNF-α and IL-6 cytokine secretion and upregulating the microglial activation marker ionized calcium-binding adaptor molecule 1 (Iba1) [45]. Intermittent hypoxia augments collagen deposition in BLM-triggered lung fibrosis. This hypoxia-induced lung inflammation activates hippocampal microglia, promoting neuroinflammation, which may potentially contribute to depressive- and anxiety-like behaviors in IPF [45].

Sun et al. (2025) demonstrate that exercise training alleviates the key pathological changes in BLM-induced lung fibrosis with depression like behavior in an animal model [108]. Pathological changes in this IPF animal model are characterized by collagen deposition and increased pulmonary inflammation, accompanied by reduced expression of brain-derived neurotrophic factor (BDNF) and c-Fos in the hippocampus region of the brain, which is associated with anxiety- and depression-like behaviors. BDNF promotes neuronal regeneration in the hippocampus and prefrontal cortex, and reduced BDNF levels in these regions contribute to atrophy and depression. c-Fos serves as a marker of neuronal activation and is linked to synaptic plasticity mechanisms underlying depression [108]. Bioinformatics analysis identified depression-associated genes S100A12 and UCHL1, both implicated in IPF progression. UCHL1 stimulates TGF-β1-induced COL1A1 overexpression in lung epithelial cells, driving collagen accumulation. S100A12, highly expressed in macrophages, promotes fibrosis via proinflammatory IL-17 and NLRP3 signaling pathways [108].

## 4. Discussion

The molecular systems architecture developed here maps the complex, multi-cellular interactions that occur within the fibrotic lung microenvironment, offering an organizing structure for interpreting IPF pathogenesis. This study of the molecular architecture of lung fibrosis provides a roadmap, showing the strong association between exposure to multiple triggering factors like environmental pollutants, genetic polymorphisms, and psychological comorbidities and the pathological mechanisms involved in IPF manifestations, contributing to systems-level understanding of IPF pathogenesis. Hence, this study can provide a multi-layered visual map of biomolecular interactions in the fibrotic lung microenvironment, an understanding of the complex crosstalk in the profibrotic fibrotic lung microenvironment, potential targets for drug development, and a framework to develop computational models providing quantitative predictions.

The results from this system architecture study provide a multi-layered visual map of biomolecular interactions in the lung microenvironment, elucidating crosstalk between profibrotic lung and CNS microenvironments, mapping targets for drug development, and establishing a framework for computational models with quantitative predictions. This multi-layered molecular systems architecture aids in visualizing molecular interactions within and across lung microenvironmental cells. These molecular interactions—as shown in Figure 4 —occur across the epithelial cells, endothelial cells, fibroblast, myofibroblast, mesenchymal stem cells, macrophages, innate lymphoid cell (ILC2), neutrophil, pulmonary smooth muscle cell, fibrocyte, pericyte, platelet and CD4^+^ T cells such as Th1 cells, Th2 cells, Th17 cells, and T regulatory cells. Using this visual map, an aberration at the level of a single molecular species (protein, receptor, promoter, gene, or miRNA)—whether a gain, loss, or overexpression of signaling—can be traced to its downstream consequences in a neighboring microenvironmental cell. Depending on the pathway affected, these cross-cellular effects can suppress or drive inflammation, myofibroblast differentiation, or tissue remodeling (encompassing matrix remodeling, vascular remodeling, and epithelial cell loss), collectively advancing IPF pathogenesis.

Although this architecture is diagrammatically static, the three biological processes it captures—namely, inflammation, myofibroblast differentiation, and tissue remodeling—broadly correspond to the major pathobiological components of IPF, including early injury-associated inflammatory signaling, fibroblast activation during disease progression, and late fibrotic tissue remodeling. Within this framework, inflammatory pathways such as TLR4, IL-33, and IFN-γ predominate during early induction, whereas TGF-β-centered pathways drive active fibrotic progression and the formation of fibroblastic foci. Remodeling-associated pathways, including LPA, galectin/Akt, HIF-1α, Notch1/PDGFβ, and MMP-19, become more prominent in late-stage disease and are accompanied by ECM accumulation and structural distortion of lung tissue.

Since IPF is often diagnosed at an advanced stage, the cellular interactions driving matrix and vascular remodeling, which contribute to clinical manifestations such as lung stiffness and pulmonary hypertension, identified in this molecular architecture study, may help in the discovery of biomarkers for earlier diagnosis. A web-based platform is under development to broaden access to this molecular systems architecture for the wider scientific community, allowing users to interactively navigate its multiple layers; this resource will be hosted at https://integrativesystems.org/open-science-institute/Pulmonary-Fibrosis/web-enabled-systems-architecture (accessed on 10 July 2026). Once available, the platform will also serve as a channel for community feedback, supporting iterative updates to the architecture as new IPF research emerges.

This molecular architecture of IPF has identified interactive crosstalk signaling across the sixteen different cell types, which were derived from IPF animal model studies that utilized various triggers such as BLM, asbestos, silica, radiation, cigarette smoke, Herpes virus, and genetic mutations. These triggers stimulate crosstalk across AECII, macrophage, fibroblast, ILC2 and immune CD4^+^ T cells, where the architecture has identified the following eight crosstalk signaling pathways: TLR4 signaling, IFN-γ signaling, TGF-β/PD-1-IL-6 signaling, IL-33 signaling, IL-4 signaling, CCL-18 signaling, Arginase signaling, and IL-37 signaling. In an interactive crosstalk signaling among macrophage, alveolar epithelial cells (AECII), fibrocytes, fibroblasts, Th17 cells, endothelial cells, and myofibroblasts, the architecture identified the following ten crosstalk signaling pathways: CXCR4/CXCL12 signaling, NLRP3 signaling, Shh signaling, thrombin/PAR-1 signaling, and six TGF-β-mediated signaling pathways such as TGF-β1/Smad signaling, TGF-β/PD-L1- β-catenin signaling, TGF-β/NOX4-4 signaling, TGF-β/Akt signaling, TGF-β1/CTGF signaling, and TGF-β/Sphingosine-1-phosphate (S1P) signaling.

Among the crosstalk between epithelial cells, endothelial cells, ECM, pulmonary smooth muscle cells, fibroblasts, pericytes, and myofibroblasts, the architecture identified the following seven crosstalk signaling pathways: notch signaling, HIF-1α signaling, LPA signaling, galectin signaling, and three TGF-β-mediated signaling pathways such as TGF-β/YAP-TAZ signaling, TGF-β/mTORC1/ATF4 signaling, and TGF-β/Smad3–p38MAPK signaling. Across endothelial cells, macrophages, pulmonary smooth muscle cells, mesenchymal cells, fibroblasts, and myofibroblasts, the architecture identified the following two crosstalk signaling pathways: IL-11 signaling and MMP-19 signaling. The molecular architecture has also revealed the role of pulmonary hypoxia induced by OSA comorbidity in triggering crosstalk among pulmonary inflammatory cells, brain neurons, and microglia, contributing to psychological disorders and disease exacerbation. These insights into profibrotic crosstalk signaling pathways provide an integrative whole-systems understanding of pulmonary fibrogenesis.

Despite evidence of CNS–lung axis crosstalk, the precise mechanism linking hypoxia-driven pulmonary inflammation to hippocampal involvement remains to be clarified in IPF. Recent evidence shows that intermittent hypoxia exacerbates pulmonary inflammation in BLM-induced fibrosis, increasing the release of pro-inflammatory cytokines, particularly TNF-α and IL-6, from the lungs, and that hippocampal microglial activation is positively correlated with these pulmonary cytokine levels [45]. This is accompanied by reduced BDNF and c-Fos expression in the hippocampus, contributing to neuronal atrophy and depressive- and anxiety-like behaviors [108]. The specific pathways by which these peripheral cytokines communicate with the central nervous system have not yet been clearly established in IPF. However, IL-6 in particular is reported to cross the blood–brain barrier and activate hippocampal microglia [45]. Furthermore, evidence from an OSA model indicates that sleep fragmentation activates microglia and drives the production of TNF-α and IL-1β, disrupting blood–brain barrier tight junctions and increasing permeability, which may permit the further entry of circulating mediators and thereby amplify neuroinflammation [109].

The molecular systems architecture presented herein displays only a topological interactome linking signaling among sixteen cell types. It does not explicitly resolve their physical spatial arrangement within lung tissue, as depicted conceptually in Figure 1. Key profibrotic mediators, including TGF-β-mediated pathways such as TGF-β/Smad, TGF-β/NOX4, TGF-β/Akt, TGF-β/CTGF, TGF-β/YAP-TAZ, and TGF-β/Smad3-p38 signaling, together with IL-11 and LPA signaling, act primarily through short-range paracrine and autocrine mechanisms. Consequently, the high cell density within fibroblastic foci is likely to amplify pathway activation, suggesting that these pathways are locally intensified within fibrotic niches rather than uniformly distributed across the lung parenchyma, and that fibroblastic foci represent the microenvironment in which this crosstalk operates most efficiently. Taken together, these localized molecular interactions converge to shape the fibrotic lung microenvironment as a coordinated disease network. This transition from pathway-level dysregulation to tissue-level remodeling provides a more concise systems-level interpretation of IPF pathogenesis.

### 4.1. Identification of Therapeutic Targets

The molecular systems architecture of IPF may provide new insights into the discovery of therapeutic targets. This IPF architecture identifies several molecular targets across different cell types in the fibrotic lung microenvironment with therapeutic potential to inhibit key biological processes, including inflammation, myofibroblast differentiation, and tissue remodeling, such as epithelial apoptosis, matrix remodeling, vascular remodeling, and impaired epithelial regeneration, that drive IPF pathophysiology. These potential targets are listed in Table 1. Candidate therapeutic agents reported in the literature for a subset of these targets are compiled in Supplementary Material S3—Table S4. A total of thirty-four molecular targets were identified within the molecular pathways that are involved in the biological processes of myofibroblast differentiation, tissue remodeling, and inflammation within the fibrotic lung microenvironment.

Among these thirty-four, seventeen targets are solely present in the molecular pathways affecting myofibroblast differentiation; eight targets are solely present in the molecular pathways affecting tissue remodeling; and one is solely present in the molecular pathways affecting inflammation. In addition, the remaining eight targets are present in molecular pathways affecting multiple biological pathways. Specifically, five targets affect myofibroblast differentiation and tissue remodeling; two targets affect myofibroblast differentiation and inflammation; and one target affects myofibroblast differentiation, tissue remodeling, and inflammation.

Although this molecular systems architecture identifies twenty-seven key molecular pathways in IPF pathogenesis, the relative contribution of each pathway may differ across patient subgroups and clinical settings, such as sporadic versus familial IPF, comorbid OSA, or predominant triggers such as environmental exposures versus genetic mutations. The targets that map to multiple biological processes, including myofibroblast differentiation, tissue remodeling, and inflammation, as summarized in Table 1, are considered higher-priority candidates than those confined to a single biological process, with TGF-β-centered pathways representing the most broadly connected drivers of IPF pathogenesis.

To provide a semi-quantitative basis for this prioritization, we calculated a literature-frequency weight for each of the thirty-four targets in Table 1, defined as the number of independent peer-reviewed references cited in support of that target (Table 1, Reference column), normalized to the highest reference count observed among the thirty-four targets (three). Targets supported by the greatest number of independent references and mapping to more than one biological process—TGF-β (three references, two processes), and Galectin-3, YAP1, and Autotaxin (two references each, two processes)—scored highest under this scheme and are considered the most robustly supported priority candidates. The majority of targets, supported by a single reference, a single biological process, or both, scored lowest. This literature-frequency-weighted ranking is intended as a semi-quantitative, the literature-derived proxy for relative target importance within the static interactome, complementing rather than replacing the dynamic network centrality or pathway enrichment analyses proposed for future CytoSolve simulations.

### 4.2. Limitations

Although this molecular systems architecture provides a comprehensive visual map of IPF pathogenesis, integrating interactions across multiple cell types and 27 key pathways of the lung microenvironment, several important limitations remain. The architecture is based on a systematic curation of the peer-reviewed literature published between 2008 and 2025 using the CytoSolve^®^/PRISMA-guided process, which may underrepresent very recent advances such as single-cell RNA sequencing (scRNA-seq) insights. Furthermore, the causal language used throughout this architecture to describe pathway connections reflects mechanistic evidence reported in the cited primary literature rather than statistically inferred causality; the interactome does not assign uniform confidence across all depicted interactions, and pathway connections should be interpreted as evidence-supported relationships rather than uniformly validated causal claims across the IPF patient population. Moreover, the relative contribution of each pathway may differ across patient subgroups and clinical contexts. While the architecture captures the role of internal triggers such as OSA, the underlying CNS–lung crosstalk mechanisms remain less characterized compared to externally driven pathways, and further preclinical studies are warranted to validate their therapeutic relevance in IPF pathogenesis. As presently constructed, the architecture represents a static interactome; dynamic CytoSolve-based simulation remains a direction for future work.

A particularly notable limitation concerns the translational relevance of animal-derived data. Studies employing murine models, including the widely used bleomycin-induced fibrosis model, cannot be fully translated to humans due to fundamental differences in species-specific physiology, metabolism, and genetics, and they frequently fail to predict therapeutic efficacy in humans. These limitations collectively highlight the need for future enhancements, including AI-assisted literature curation, multi-omics integration for parameterization, dynamic CytoSolve-based computational modeling, and targeted clinical validation studies to strengthen the translational applicability of this architecture.

## 5. Conclusions and Future Directions

The molecular systems architecture of the fibrotic lung microenvironment in IPF may enable the discovery of multi-combination therapies by activating or inhibiting more than one molecular target that affects multiple biological processes implicated in IPF pathogenesis. Moreover, the architecture could provide insights into how targeting one or more mechanisms in the fibrotic lung microenvironment affects the biological process in either a positive or negative therapeutic outcome. The interactome system derived from this molecular architecture study provides a platform for topological analysis of disease networks from protein–protein interactions among multiple types of cells or subsystems, which lays a foundation for the computational modeling of IPF disease. Previous architecture studies, such as the molecular systems architecture of pancreatic cancer, were successfully used to develop computational models that predicted the dosages of a two-drug combination that was allowed by the U.S. Food and Drug Administration (FDA) [122]. Additionally, in silico models based on a molecular systems architecture of osteoarthritis were used to derive an optimal combination of two bioflavonoids targeting molecular pathways of pain and inflammation [38].

Molecular systems architectures, such as the one presented herein, provide a framework for the development of such mechanistic in silico modeling efforts. CytoSolve’s computational capabilities can be utilized to develop an integrative model of the fibrotic lung microenvironment by incorporating data from in vitro and in vivo studies to predict disease behavior and therapeutic responses, particularly given the spatial heterogeneity of lung fibrosis. To convert the static interactome into a dynamic simulation model, future work should incorporate quantitative kinetic parameters such as cell-type-specific secretion rates, receptor–ligand binding constants, and protein half-lives or degradation rates for key signaling mediators, including TGF-β and IL-11, curated from published biochemical and kinetic literature databases. Furthermore, time-resolved parameter sets would enable ordinary differential equation (ODE) simulations to capture pathway activity across the stages of early induction, active progression, and late remodeling.

Spatially resolved approaches will be required to determine whether dense fibroblastic foci form signaling niches that intensify profibrotic crosstalk. Recent spatial transcriptomic studies in IPF have identified disease-associated fibrotic niches and fibroblastic foci [123]. Future refinement of this systems architecture could address this by incorporating spatial interaction parameters or coupling the architecture with agent-based modeling to predict whether the 16 identified cell types are co-localized and functionally connected within fibrotic microenvironments. The parameters for such simulations could be derived from spatially resolved IPF datasets, such as spatial transcriptomics, and integrated into the CytoSolve model as spatial and cell-density parameters.

To further enhance the human translational relevance of these target predictions, human cell-level data emerging from patient-derived induced pluripotent stem cell (iPSC) three-dimensional lung organoids and lung-on-a-chip models can be integrated as parameters and validation benchmarks for the in silico model. The resulting in silico IPF lung model may be used as a platform for testing, validation, and prioritization of the potential targets identified by the molecular systems architecture in Table 1. Of the molecular targets compiled in Table 1, the present CytoSolve-based systems architecture identified 34 that may be mechanistically relevant to the disease process and could subsequently be tested by this model for their relative effectiveness in reducing inflammation, myofibroblast differentiation, and tissue remodeling, including epithelial apoptosis, matrix and vascular remodeling, while promoting epithelial regeneration. Lung-on-chip models may provide a systems-based approach to accelerate the verification of in silico predictions. Beyond target prioritization, the CytoSolve model can be employed to perform in silico combinatorial simulations in which two or more candidate compounds, directed against the prioritized targets, are evaluated together to predict synergistic efficacy and reduced off-target effects relative to single-agent treatment. Such an approach could improve the efficacy and safety of multi-combination therapeutics and provide a quantitative basis for identifying rational multi-target regimens for the effective management of IPF.

The same modeling framework could be extended to the lung–CNS crosstalk axis by developing a multi-compartment CytoSolve model parameterized from published cytokine and neuroinflammation data. This model could be used to evaluate the predicted contributions of lung-derived TNF-α and IL-6 to hippocampal microglial activation through computational perturbation analysis. The simulated neutralization or downregulation of these mediators would provide an in silico approach for testing the proposed lung-to-CNS signaling axis and its potential role in the psychological comorbidities of IPF. Taken together, the architecture developed herein can serve as a powerful tool to identify molecular targets for new therapeutic interventions and to support the development of prognostic biomarkers for clinical management, moving beyond current drug development, which typically seeks a single compound acting on a single target to inhibit or delay IPF progression.

## Figures and Tables

**Table 1:** Molecular therapeutic targets.

S. No	Molecular Target	Cell Type	Biological Process			Reference
			MD	IN	TR	
1.	PAD2	Alveolar macrophage		✓		[54]
2.	eCIRP	Alveolar macrophage, fibroblast		✓		[55]
3.	TGF-β	Alveolar macrophage, pericyte	✓		✓	[30,36,62]
4.	CCL-18	Macrophage M2		✓		[62,110]
5.	Arginase 1	Macrophage M2		✓		[111]
6.	IL-33	AECII, ILC2, alveolar macrophage, neutrophil	✓	✓		[112]
7.	IL-37	AECII, alveolar macrophage, fibroblast		✓		[68]
8.	CXCR4	Fibrocyte		✓		[76,113,114]
9.	PD-1	CD4^+^ T regulatory and Th17 cells.	✓	✓		[68]
10.	αvβ6 integrin	AECII		✓		[70]
11.	HIF-1α	Pulmonary artery smooth muscle cell		✓		[96]
12.	IL-4	Th2 cell		✓		[29,36]
13.	IL-13	Th2 cell, macrophageM2, ILC2		✓		[29,36]
14.	CD44V6	Fibroblast		✓		[115,116]
15.	NOX4	Fibroblast		✓		[115,116]
16.	Galectin-3	Pulmonary vascular endothelial cell	✓		✓	[30,94]
17.	CTGF	AECII, fibroblast		✓		[117,118]
18.	NLRP3 inflammasome	AECII, alveolar macrophage		✓		[80,82,84]
19.	Shh	AECII		✓		[119]
20.	SPHK1	Fibroblast		✓		[120]
21.	YAP1	Fibroblast	✓		✓	[120,121]
22.	Autotaxin	AECII, Fibroblast	✓		✓	[30,90]
23.	LPA1	AECII		✓		[36,90]
24.	LPA2	Fibroblast		✓		[90]
25.	Notch 1	Pericyte		✓		[97]
26.	HK2	Myofibroblast		✓		[101]
27.	PFKFB3	Myofibroblast		✓		[101]
28.	mTOR-ATF4 axis	Fibroblast		✓		[88]
29.	GLS	Fibroblast, myofibroblast		✓		[101]
30.	IL-11	Pulmonary artery smooth muscle cell, vascular endothelial cell, fibroblast	✓		✓	[105]
31.	MMP19	Endothelial cell, alveolar macrophage.	✓	✓	✓	[87]
32.	PAI-1	Fibroblast		✓		[96]
33.	miR-15a	Fibroblast		✓		[122]
34.	miR-146b	Pericyte		✓		[98]

PAD2—peptidyl arginine deiminase 2; eCIRP—extracellular cold-inducible RNA-binding protein; TGF-β—transforming growth factor beta; CCL18—C-C motif chemokine ligand 18; IL—interleukin; CXCR4—C-X-C Motif Chemokine Receptor 4; PD-1—programmed cell death protein 1; αvβ6 integrin—alpha-v-beta-6 integrin; HIF-1α—hypoxia-inducible factor-1 subunit alpha; CD44V6—CD44 variant exon 6; CTGF—connective tissue growth, factor; Shh—sonic hedgehog; SPHK1—sphingosine kinase 1; YAP-1—YAP—Yes-associated protein; LPA1—lysophosphatidic acid receptor 1; LPA2—lysophosphatidic acid receptor 2; PFKFB3 —phosphofructo-2-kinase/fructose-2, 6-bisphosphatase 3; HK2—hexose kinase 2, GLS—glutaminase; MMP19—matrix metalloproteinase 19; PAI—plasminogen activator inhibitor.

## Data Availability

The original contributions presented in this study are included in the article/Supplementary Material. Further inquiries can be directed to the corresponding authors.

## Supplementary Materials

The following supporting information can be downloaded at: https://www.mdpi.com/article/10.3390/cells15151364/s1, References cited within the Supplementary Materials are provided in the main-text reference list [124,125,126,127,128,129,130,131,132,133,134,135,136,137,138,139,140,141,142,143,144,145,146,147,148,149,150,151,152,153,154,155,156,157,158,159,160,161,162,163,164,165,166,167,168,169,170].

## Abbreviations

MUC5B	Mucin 5B
EBV	Epstein–Barr virus
CMV	Cytomegalovirus
KSHV	Kaposi’s sarcoma herpes virus
SFTPC	Surfactant protein C
Mfn	Mitofusion
HMGB1	High-Mobility Group Box 1
TOLLIP	Toll-interacting protein
CCL	C-C motif Chemokine Ligand
CCR	C-C motif Chemokine Receptor
CIITA	class II major histocompatibility complex
iNOS	Inducible nitric oxide synthase
IL	Interleukin
STAT	Signal Transducer and Activator of Transcription
IFN-γ	Interferon-gamma
JAK	Janus Kinase
PI3K	Phosphoinositide 3-kinase
Akt	Protein Kinase B (PKB)
NF-κB	Nuclear Factor kappa-light-chain-enhancer of activated B cells
IRF3	Interferon Regulatory Factor 3
CD	Cluster of differentiation
SHP-2	Src homology 2 domain-containing protein tyrosine phosphatase-2
LC3 II	Microtubule-associated protein 1A/1B-light chain 3-II
ATG-7	Autophagy Related 7
Smad	Small Mothers Against Decapentaplegic
Rho	RAS homolog
MMP	Matrix Metalloproteinase
FGF	Fibroblast Growth Factor
PDGF	Platelet-Derived Growth Factor
VEGF	Vascular Endothelial Growth Factor
SASP	Senescence-Associated Secretory Phenotype
MAPK	Mitogen-activated protein kinase
SP1	Specificity protein 1
AP-1	Activator protein 1
WNT	Wingless-related integration site
GSK-3β	Glycogen synthase kinase-3β
PKC	Protein kinase C
ERK	Extracellular signal-regulated kinase
