# Supplementary material for: Molecular Systems Architecture of Fibrotic Lung Microenvironment in Idiopathic Pulmonary Fibrosis
Source: Cells. 2026 Jul 29;15(15):1364. doi: 10.3390/cells15151364 (PMC13464394; doi:10.3390/cells15151364)
Supplement: Supplementary file 1 [file cells-15-01364-s001.zip › cells-4403347-supplementary.pdf]

Supplementary Information

Molecular Systems Architecture of Fibrotic Lung Microenvironment in Idiopathic Pulmonary Fibrosis

V.A. Shiva Ayyadurai[1,2*], Yamuna Manoharan., Prabhakar Deonikar[1,2]
Systems Biology Group, CytoSolve Research Division, CytoSolve, Inc., Cambridge, MA
[2]Open Science Institute, International Center for Integrative Systems, Cambridge, MA

Table of Contents

Supplementary Material S1: Additional information on molecular pathways contributing to pulmonary fibrosis pathogenesis.

Supplementary Material S2: Figures S1-S14 provide a schematic representation of molecular pathways contributing to pulmonary fibrosis pathogenesis

Supplementary Material S3: Table S1, S2, S3, and S4.

Supplementary Material S4: CytoSolve® Operating Guide Protocol

Supplementary Material S5 - Glossary – Main Manuscript and Supplemental Figures

Supplementary Material S1

Arginase-1 Signaling: Arginase-1 (Arg-1) overexpression, observed in BLM-induced mice and IPF patients, contributes to collagen accumulation in pulmonary fibrosis [1–3]. High Arg-1 expression occurs in epithelial cells, fibroblastic foci, and alveolar macrophages of patients with herpesvirus-induced pulmonary fibrosis [3]. Th2 cytokines in the infected lung microenvironment boost Arg-1 production in macrophages and fibroblasts, metabolizing L-arginine to L-ornithine, L-proline, and polyamines. These metabolites drive fibroblast proliferation, collagen synthesis, and myofibroblast differentiation, promoting fibrosis [3].

CCL18 Signaling: Higher levels of CCL18 have been reported in serum and BALF of patients with IPF [4]. M2 macrophage-derived CCL18 binds to CCR8 on fibroblasts, activating ERK1/2 signaling, which phosphorylates SP-1 and enables its synergistic interaction with Smad3, thereby driving collagen I synthesis [4–6]. Accumulated collagen then binds to the alveolar macrophage receptor CD204, triggering Akt signaling that reinforces M2 polarization by increasing CCL18 production [4,6]. This CCL18-mediated feedback loop perpetuates M2 activation, collagen deposition, and IPF progression, leading to a decline in lung function [4,6].

IL-33/IL-13 Signaling axis: The BLM-induced pulmonary fibrosis animal model has established the role of the IL-33-ILC2-IL-13 axis in generating a Th2 microenvironment, which induces macrophage polarization to the M2 phenotype [7]. Li et al. (2014) showed that BLM induces damaged epithelial cells to secrete full-length IL-33, CXCL1, CXCL2, and CCL2. CXCL1/CXCL2 recruit neutrophils, while CCL2 drives monocyte/macrophage and lymphocyte infiltration into inflamed lung tissue [7]. Neutrophil cathepsin G processes full-length IL-33 into mature IL-33 (mIL-33), which binds to the ST2L receptor on macrophages

and ILC2s, activating NF-κB and inducing IL-13 secretion [7,8]. IL-33/ST2L signaling in bone marrow progenitors induces TGF-β and ILC2 differentiation. These BM-derived ILC2s traffic to fibrotic lungs, activating fibroblasts via TGF-β paracrine signaling and driving collagen deposition [9]. IL-13/IL-4Rα and mIL-33/ST2L pathways synergistically promote M2 macrophage polarization, upregulating Arg-1, CD206, and TGF-β1, which further activate fibroblasts and exacerbate fibrosis [7,9].

TGF-β/PD-L1- β-catenin Signaling: Moreover, studies have implicated the role of TGF-β/PD-L1 signaling in the induction of fibroblast to myofibroblast transition (FMT), driving the progression of pulmonary fibrosis [10,11]. Mechanistically, Th17-derived TGF-β stimulates downstream signaling by phosphorylating Smad3 and p38, which increases the expression of PD-L1 in fibroblasts [11]. Guo et al. (2022) showed that nuclear PD-L1 interacts with Smad3 as a co-transactivator, upregulating FMT genes such as collagen, fibronectin, and α-SMA. PD-L1 also enhances TGF-β-induced GSK-3β phosphorylation (Ser9), stabilizing β-catenin [11]. This β-catenin then binds nuclear TCF to drive FMT gene transcription (X. Guo et al., 2022). Thus, PD-L1 amplifies TGF-β profibrotic signaling via Smad3, p38-MAPK, and GSK-3β pathways, promoting pulmonary fibrosis progression [11]. Additionally, Th17 cell-derived TGF-β stimulates the epithelial-mesenchymal transition (EMT) by upregulating E-cadherin expression and downregulating vimentin and α-SMA [12].

TGF-β/Nox-4 Signaling: Evidence shows elevated Nox4 expression in pulmonary arterial smooth muscle cells, endothelial cells, and alveolar epithelial cells of the lung tissue from BLM-induced IPF animal models and IPF patients [13]. As an NADPH oxidase, Nox4 catalyzes the reduction of molecular oxygen to produce ROS [14]. IPF often leads to secondary pulmonary hypertension (PH) via pulmonary arteriole remodeling and increased

vascular resistance, driven by alveolar destruction, reduced vascular bed, and hypoxia [13]. TGF-β signaling upregulates Nox4, which is critical for TGF-β-dependent fibrotic responses, including ECM formation, proliferation, and PAI-1 synthesis, in IPF lung fibroblasts [13]. Liu et al. (2010) demonstrated that TGF-β1 signaling increases nuclear Nox4 expression in NIH3T3 fibroblasts, boosting ROS/hydrogen peroxide (H_2O_2) production. This ROS inactivates nuclear MAPK phosphatase (MPK-1), activating p38 and JNK MAPKs, which stimulate AP-1 and Sp1 transcription factors, respectively. Smad3 synergizes with AP-1 and Sp1 to upregulate PAI-1 [13,15]. Jarman et al. (2014) confirmed elevated PAI-1 in lung fibroblasts from BLM-induced pulmonary fibrosis mice, linking it to reduced fibrinolysis and ECM accumulation [13,15]. Additionally, the TGF-β1/Nox4/ROS/p38 pathway drives ECM protein expression (collagen 1α1, 3α1, fibronectin, α-SMA) and fibroblast-to-myofibroblast differentiation [13,16].

Ghatak et al. (2017) showed that elevated CD44 containing variable exon 6 (CD44V6) expression activates the TGF-β1/p-Smad3 pathway, inducing Nox4 in BLM-treated mouse lung fibroblasts. Nox4-derived ROS then activates AP-1 to transcribe CD44V6, forming a positive feedback loop that amplifies oxidative stress and fibrosis [17]. Ghatak et al. (2016) identified the TGF-β1-ERK-EGR1/AP-1-CD44v6 axis, where TGF-β1 activates hyaluronan receptor CD44V6 signaling in fibroblasts to drive differentiation via ERK/EGR1/AP-1 activation [18]. In IPF lung fibroblasts, BLM-induced TGF-β1 overexpression triggers autocrine ERK phosphorylation, EGR1 nuclear translocation, AP-1 complex activation, and enhanced AP-1 binding to the CD44V6 promoter, thereby upregulating its expression [18]. TGF-β1 further activates NOX-4/ROS signaling to increase hyaluronan synthase (HAS2) production in activated lung fibroblasts [17,18]. Enhanced hyaluronan binding to CD44V6 then promotes CD44V6-TβRI interaction, initiating TGF-β1/ERK/EGR1-dependent fibroblast

differentiation. This CD44v6-TGF-β1/ERK/EGR1 positive feedback loop sustains persistent ERK/EGR1/AP-1 signaling to maintain a fibrogenic phenotype in fibroblasts [18].

TGF-β1/CTGF Signaling: _CTGF, also known as CCN2, a cysteine-rich heparin-binding protein of the CCN family, correlates with elevated ECM production, fibroblast activation, adhesion, invasion, tissue remodeling, and fibrosis in IPF patients [19,20]. Secreted by alveolar epithelial type II cells (AECII) and activated fibroblasts via autocrine and paracrine mechanisms, CTGF serves as a key downstream effector of TGF-β1/PI3K/Akt signaling, linking it to collagen production in epithelial cells [19,21]. Epithelial-derived CTGF further promotes myofibroblast differentiation from lung fibroblasts [21], while activating Rac1/MLK3/JNK signaling to recruit c-Jun and c-Fos to form AP-1, driving collagen I synthesis in human lung fibroblasts [22,23]. Additionally, CTGF binds cell surface integrins to activate integrin-linked kinase (ILK), inducing epithelial-mesenchymal transition (EMT) in AECII cells and upregulating mesenchymal markers (fibronectin, vimentin, α-SMA) [24]. miRNAs such as miR-26a and miR-30c-5p target CTGF to inhibit fibroblast and epithelial transdifferentiation into myofibroblasts, respectively [22].

TGF-β/Sphingosine-1-phosphate (S1P) Signaling: Evidence indicates that sphingolipids, specifically sphingosine 1-phosphate (S1P) and lysophosphatidic acid (LPA), play important roles in the development of IPF [2]. Reports have shown the significant association between reduced survival rates and higher expression of sphingosine kinase 1 (SPHK1) in the lung tissues of individuals with IPF. Elevated amounts of S1P have also been identified in blood and bronchoalveolar lavage fluid (BALF) [9,25]. Sphingomyelin, a sphingolipid in cell membranes composed of phosphocholine or phosphoethanolamine bound to ceramide, is metabolized by sphingomyelinase (SMase) to produce ceramide (Cer) [26]. Ceramidase then

converts Cer to sphingosine (Sph), which sphingosine kinase 1 (SphK1) phosphorylates to sphingosine-1-phosphate (S1P) [26].

S1P is a pleiotropic lysophospholipid mediator that functions through five G protein-coupled receptors (S1PRs), which regulate several biological processes, including lymphocyte trafficking and vascular barrier integrity [9]. Data from Huang et al. (2020) show that in lung fibroblasts, BLM-induced TGF-β1 upregulates Sphk1 expression and, consequently, increases S1P levels (L. S. Huang et al., 2020). This S1P, then via its interaction with S1P receptor, activates the nuclear translocation of Yes-associated protein 1 (YAP-1) and TAZ, where it transactivates the expression of fibronectin (FN) and α-SMA, promoting the myofibroblast differentiation (L. S. Huang et al., 2020).

Thrombin/PAR-1 Signaling: Findings by Song et al. (2013) have demonstrated the profibrotic effect of thrombin, in addition to its traditional role in blood coagulation [28]. Thrombin promotes EMT by activating PAR-1 in AECII, contributing to the generation of myofibroblasts [28]. BLM upregulates PAR-1 in A549 cells, and thrombin-activated PAR-1/PKC signaling induces ERK1/2 phosphorylation, promoting EMT via elevated profibrotic markers (collagen I, TGF-β1, α-SMA) [28].

TGF-β/Akt Signaling: Experimental studies have demonstrated that BLM and cigarette smoke induced TGF-β-dependent non-Smad signaling via the activation of PI3K/AKT/GSK-3β/Snail1 pathway, promoting the EMT and development of pulmonary fibrosis [29]. Activated AKT phosphorylates GSK-3β at serine 9, inactivating it and thereby stabilizing β-catenin while promoting SNAIL1 accumulation in alveolar epithelial cells [29,30]. Upon nuclear translocation, Snail1 and β-catenin synergistically regulate EMT gene transcription by

repressing E-cadherin and upregulating mesenchymal markers (vimentin, α-SMA, fibronectin), thereby driving EMT in pulmonary fibrosis pathogenesis [29,31].

CXCR4/CXCL12 Signaling: Epithelial-derived CXCL12 activates CXCR4 signaling in lung fibroblasts, stimulating Rac/ERK and JNK pathways. These phosphorylate c-Jun, inducing AP-1 complex formation and transactivation of CTGF, which upregulates α-SMA to promote fibroblast-to-myofibroblast transition [32].

NLRP3 signaling: Inflammasome activation is triggered by PAMPs or DAMPs via TLR4, which activate NF-κB to upregulate pro-caspase-1, pro-IL-1β, pro-IL-18, and NLRP3 and subsequently increase the transcription of components, including pro-caspase-1, pro-IL-1β, pro-IL-18, and NLRP3, through the activation of the NF-kB pathway [33,34]. The NLRP3 inflammasome recruits the ASC adaptor to activate caspase-1, which cleaves pro-IL-1β and pro-IL-18 into mature forms [34,35]. Recent evidence links alveolar epithelial injury to NLRP3 activation, driving profibrotic EMT. BLM and silica models show elevated p-p65/p65 ratio, NLRP3, caspase-1, ASC, IL-1β, and IL-18 in alveolar/bronchial epithelial cells [34,36]. Silica PM2.5 induces EMT in bronchial epithelial cells (16HBE) through persistent NLRP3 inflammasome activation via the IL-1β/TAK1/MAPK/NF-κB/Snail pathway [36]. Inflammasome-derived IL-1β binds epithelial IL-1R, recruiting MyD88 and IL-1 receptor-associated kinases (IRAKs) to phosphorylate TGF-β-activated kinase 1 (TAK1) and MAPK-p38. TAK1/MAPK-p38 then activates NF-κB, upregulating Snail to drive EMT and pulmonary fibrosis [36].

Inflammasome-derived IL-1β inhibits epithelial DKK1 secretion, activating canonical Wnt/β-catenin signaling in lung-resident mesenchymal stem cells (LR-MSCs), which are pluripotent

stem cells residing in the lung microenvironment [37]. DKK1, an endogenous Wnt antagonist, normally binds LRP5/6 receptors to downregulate β-catenin [38]. Wnt ligands bind Frizzled (FZD) and low-density lipoprotein receptor-related proteins 5 and 6 (LRP5/6 coreceptors), inactivating the β-catenin destruction complex, which consists of axin, adenomatous polyposis coli protein (APC), casein kinase 1, and glycogen synthase kinase (GSK)-3β, that targets β-catenin for phosphorylation (Ser33/37, Thr41), ubiquitination, and proteasomal degradation [38,39]. Inactivation of GSK-3β results in dephosphorylation of catenin at Ser-33/37 and Thr41, stabilizing the β-catenin in the cytoplasm [38]. Cytoplasmic β-catenin translocates to the nucleus, where it binds to TCF (T cell factor) and LEF (lymphoid enhancer binding factor) to activate the transcription of Wnt target genes [39]. Thus, IL-1β-driven DKK1 inhibition activates Wnt signaling, upregulating α-SMA and collagen to promote LR-MSC differentiation into myofibroblasts and exacerbate pulmonary fibrosis [37].

LPA signaling: LPA activates TGF-β in epithelial cells through the αvβ6 integrin-mediated LPA2-RhoA/Rho kinase pathway. LPA-LPA2 signaling also triggers p38 MAPK, Akt, and ERK1/2 cascades in fibroblasts, boosting TGF-β expression. This secreted TGF-β then activates Smad2/3 to upregulate profibrotic markers like fibronectin, Col1A2, and α-SMA.Findings show that LPA activates TGF-β in epithelial cells via the αvβ6 integrin-mediated LPA2-RhoA/Rho kinase cascade [26,40]. IPF patients exhibit elevated LPA2 and αvβ6 integrin levels per clinical data, indicating LPA2 as a potential therapeutic target. [26].

Shh/Wnt10a signaling: Sonic hedgehog (Shh), a hedgehog family protein, regulates embryonic tissue patterning and organogenesis. Shh, secreted by lung epithelial cells, drives mesenchymal cell proliferation. In IPF lungs, reactive AECII show elevated Shh expression compared to low levels in normal adult lungs (Sakai & Tager, 2013). LR-

MSCs critically contribute to pulmonary fibrosis pathogenesis, as shown in bleomycin models. Activated Wnt10a signaling drives myofibroblastic differentiation of LR-MSCs. Shh secreted by injured lung epithelial cells upregulates Wnt10a expression in these LR-MSCs.[42]. Activated epithelial Shh ligand interacts with the patched (Ptc) receptor on the cell surface of LR-MSCs, and this Shh-Ptc receptor interaction induces the release of sequestered co-receptor smoothened (Smo), which in turn activates the transcription factor glioma-associated oncogene homolog (Gli) [42,43]. Before activation, Gli is sequestered in a kinase complex, comprising protein kinase A (PKA), glycogen synthase kinase 3 (GSK-3β), and casein kinase-1 (CK1). Activated Gli is released from the kinase complex and translocates to the nucleus, promoting the transcription of the downstream target gene Wnt10a [42]. Increased level of Wnt10a expression has been shown to trigger fibrogenesis via binding to Frizzled10 (Fzd) receptor on LR-MSCs. Subsequently, the low-density lipoprotein receptor-related protein (LRP) forms a complex with Wnt-bound Fzd receptor, activating the phosphorylation of its downstream target β-catenin at Ser-675 [42]. Activated p-β-catenin promotes the transcription of α-SMA, collagen I, and fibronectin and inhibits the expression of LR-MSC marker Sca-1, leading to transdifferentiation of LR-MSCs into myofibroblasts, thereby augmenting the pathogenesis of pulmonary fibrosis [42].

TGF-β/YAP-TAZ signaling: Dysregulation of the Hippo pathway, which is well recognized for mediating the mechano-transduction signaling, has been found to play a critical role in pulmonary fibrosis [44]. Evidences show that the hippo pathway can converge with mechanotransduction and TGF-β signaling pathways to activate fibroblasts, suggesting dual roles in IPF [44]. Research has shown that in response to TGF-β stimulation, TAZ forms complexes with Smad2/3, which then translocate to the nucleus. This complex upregulates the

transcription of target genes, including Col 1α1, Col 3α1, fibronectin, and CTGF, thereby promoting lung fibroblast proliferation, migration, and collagen production, key processes in pulmonary fibrosis pathogenesis [45]. Moreover, Chen et al. (2019) reported the miR-15a downregulation in IPF, which has been shown to negatively regulate the YAP-1 mRNA expression, as depicted in Figure 10 [46]. Elevated matrix stiffness has been implicated in the mechanotransduction signaling, which activates the transcription factors YAP and TAZ, promoting the expression of the SERPINE1 gene encoding PAI-1 protein in fibroblasts. PAI-1-dependent inhibition of plasmin on rigid matrices facilitates the production of procollagen I and α-SMA, which are important indicators of fibroblast activation [47]. This downregulation of plasmin activity has been shown to promote sustained nuclear localization of YAP-TAZ in a positive feedback loop manner, leading to the maintenance of a profibrotic state [47].

TGF-β/YAP-TAZ-MKL-1 signaling axis: Sava et al. (2017) showed that resident fibroblast-derived TGF-β1 promotes the differentiation of pericytes into myofibroblasts expressing collagen, fibronectin, α-SMA, and vimentin, leading to extracellular matrix (ECM) accumulation and increased tissue stiffness [48]. Matrix stiffening activates mechanotransduction pathways that drive nuclear translocation of MKL-1, thereby enhancing profibrotic gene expression and α-SMA+ myofibroblast accumulation. The interaction between focal adhesion kinase (FAK) with focal adhesion-integrin complex induced by stiff matrix further promotes MKL-1, YAP, and TAZ nuclear translocation, reinforcing pericyte-to-myofibroblast transition, as shown in Figure15 [48]. Treatment with nintedanib reduces α-SMA levels and enhances MMP2 and MMP9 activity, thereby restoring the elasticity of fibrotic lung tissues [48].

TGF-β/mTORC1/ATF4 signaling axis: Glycine is one of the primary amino acids that participates in collagen synthesis. Findings by Selvaraj et al. (2019) indicate that TGF-β1 binds to its receptor complex and activates Smad3-dependent increases in activating transcription factor 4 (ATF4) mRNA transcription as well as mechanistic target of rapamycin complex 1 (mTOR) activation [49]. Activated mTORC1 stimulates its downstream target eukaryotic translation initiation factor 4E-binding protein 1 (4E-BP1), which in turn promotes ATF4 translation, resulting in enhanced ATF4 protein production [49]. This ATF4 induces the expression of the major enzymes involved in the de novo serine-glycine biosynthetic pathway, such as phosphoglycerate dehydrogenase (PHGDH), phosphoserine aminotransferase 1 (PSAT1), phosphoserine phosphatase (PSPH), and serine hydroxy methyltransferase 2 (SHMT2) [1,2,49]. Moreover, ATF4 also shown to increase the expression of Glucose transporter 1 (GLUT1), enhancing glycolysis, where the glycolytic intermediate 3-phosphoglycerate (3-PG), acts as a substrate for de novo serine-glycine biosynthesis, and consequently, collagen production in activated myofibroblast [1,2,50]. Pharmacological findings have shown that the inhibition of key glycolytic enzymes, such as by inhibitors, leads to the suppression of TGF–β1–induced myofibroblast differentiation and collagen production [50].

IL-11 signaling: IL-11/IL-11Rα signaling boosts expression of fibrotic mediators like p-ERK1/2, p-JAK2/p-STAT3, p-AKT, and p-SMAD2/3, promoting myofibroblast transition in endothelial and pulmonary smooth muscle cells. TGF-β induces JAK-2 phosphorylation in IPF-derived HPASMCs and HPAECs [51]. JAK/STAT3 activation is required for TGF-β-driven Smad3 phosphorylation and its transcriptional enhancement of EndMT [52]. Overall, IL-11/IL-11Rα phosphorylates GP130 and JAK2/STAT3, activating SMAD2/3 to drive myofibroblast differentiation in these cells[51,53].

Supplementary Material S2

S1. IL-33/L-13 signaling axis

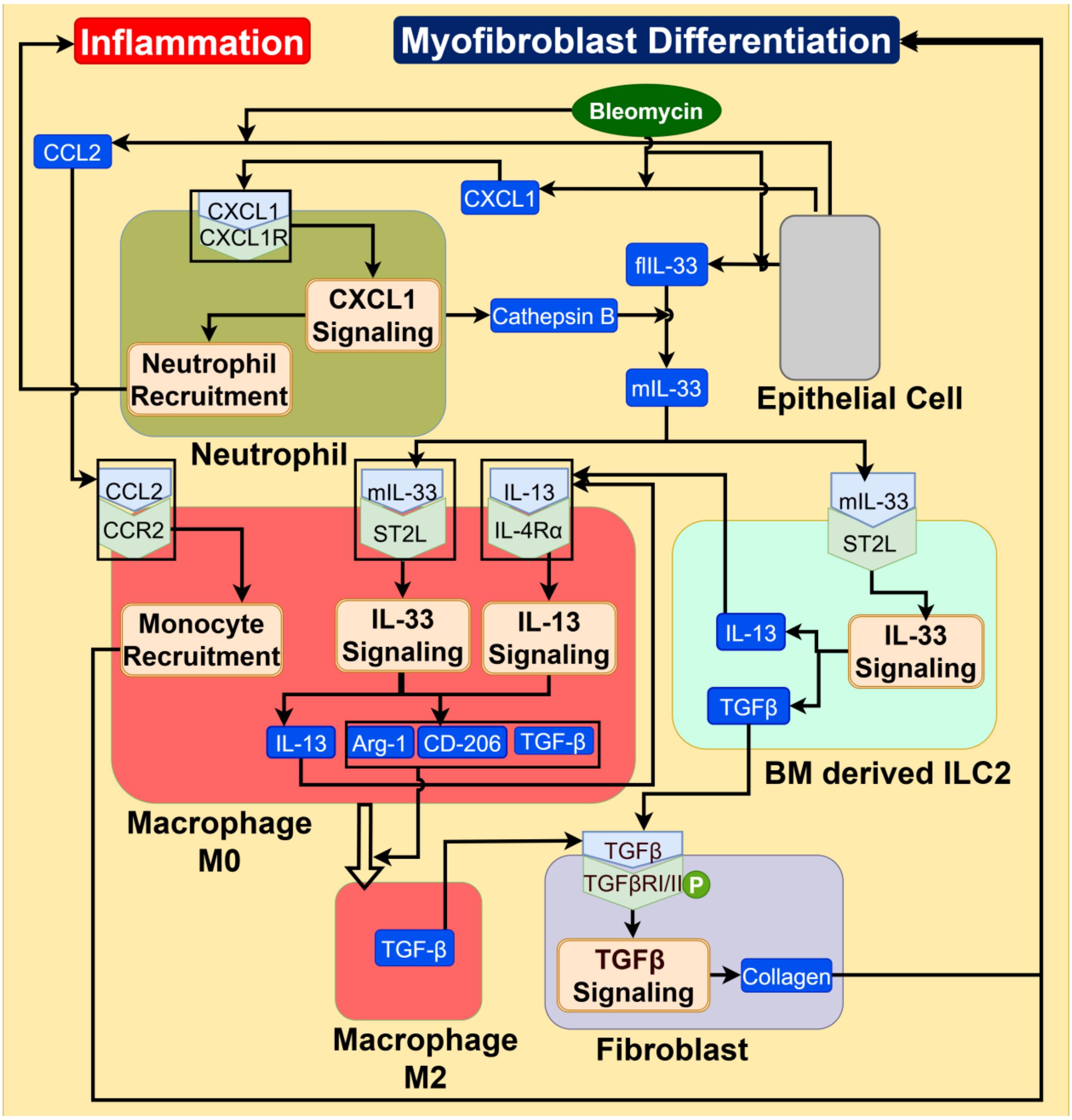

Figure S1: IL-33/IL-13 signaling axis. mIL-33 signaling in ILC2 and both mIL-33 and IL-13 signaling in macrophages synergistically contribute to pulmonary fibrosis pathogenesis by promoting the M2 polarization and fibroblast activation, contributing to myofibroblast differentiation [7]. IL – Interleukin; ILC2 - Type 2 innate lymphoid cells; flIL-33 - full length IL-33; mIL-33 – mature IL-33; IL-4Rα - interleukin (IL)-4 receptor alpha; CCL2 - C-C motif chemokine ligand 2; CXCL1 - chemokine (C-X-C motif) ligand 1; TGF-β – Transforming growth factor beta.

S2. TGF-β/PD-L1 signaling

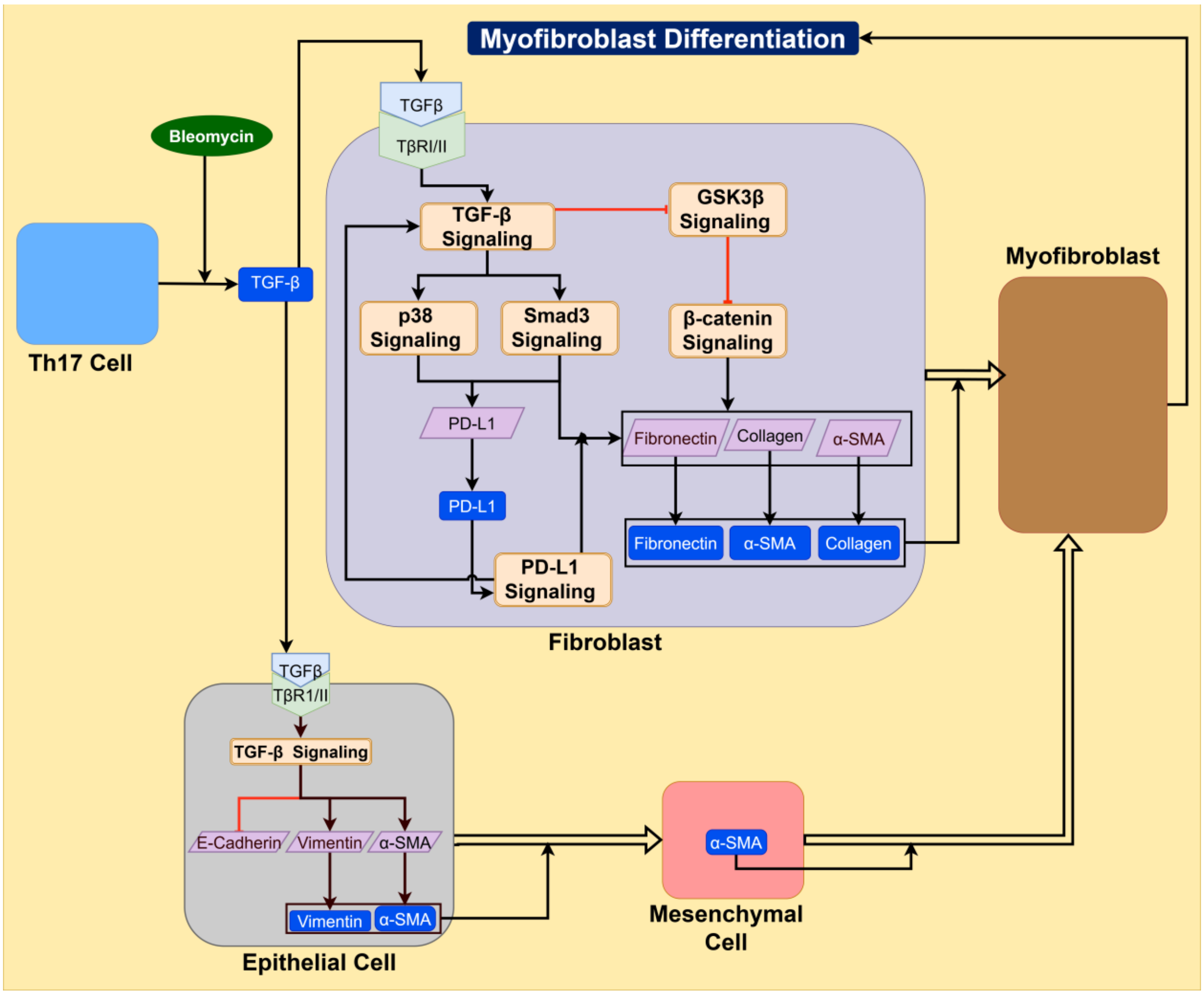

Figure S2: TGF-β/PD-L1 signaling. Th17 cell-derived TGF-β1 induces PD-L1 signaling in fibroblasts, stimulating fibroblast differentiation through the process of FMT, contributing to myofibroblast differentiation. The TGF-β1 signaling in AECII also induces EMT, leading to myofibroblast differentiation [11,12]**.** PD-L1 - Programmed death-ligand 1; α-SMA - Alpha-smooth muscle actin; GSK-3β - Glycogen synthase kinase-3 beta.

S3. TGF-β/Nox-4 signaling

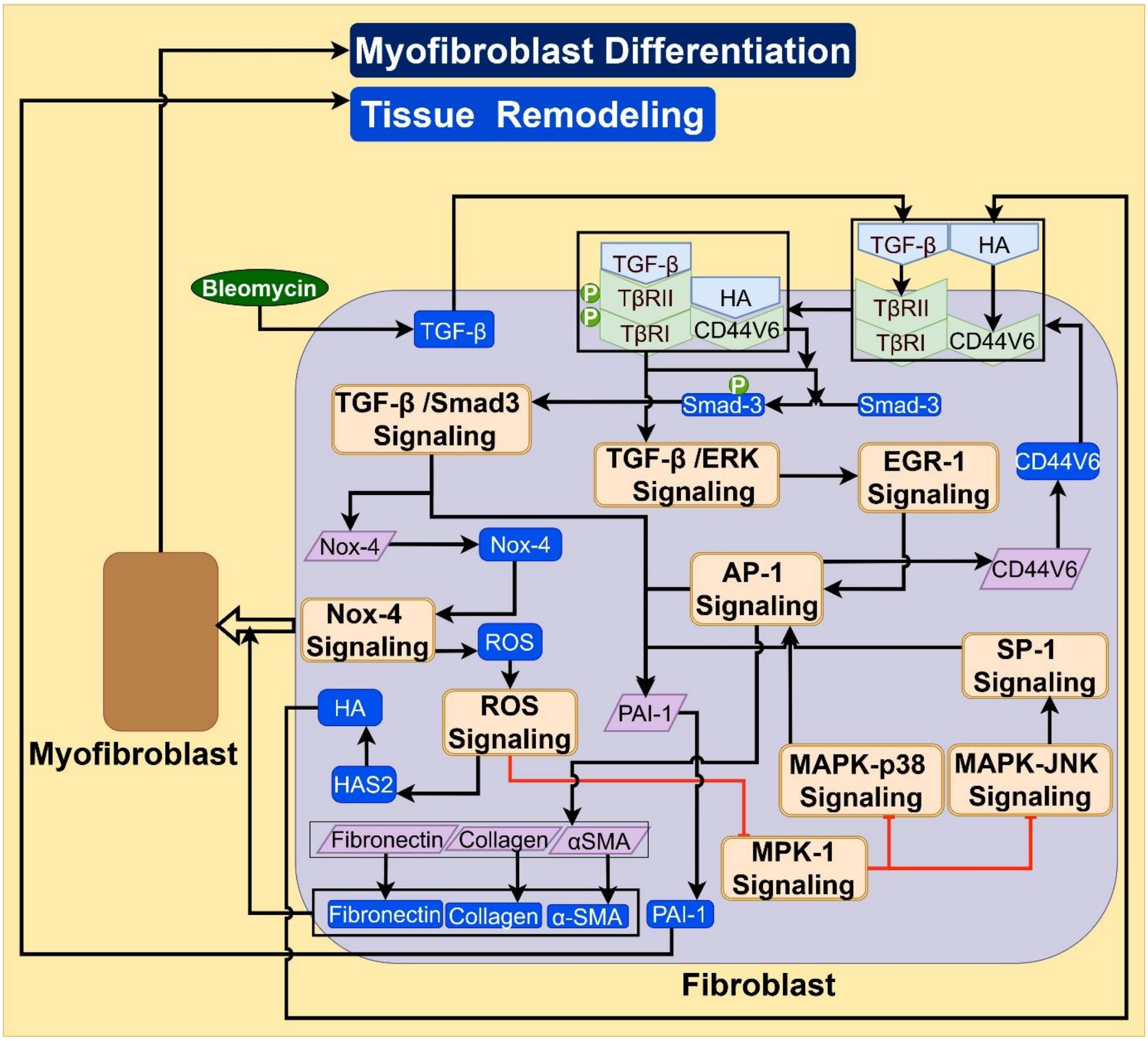

Figure S3: TGF-β/Nox-4 signaling. TGF-β/Nox-4 signaling in fibroblasts contributes to pulmonary fibrosis pathogenesis by promoting the FMT process, contributing to myofibroblast differentiation and matrix remodeling [15,17,54]. NOX-4 – NADPH oxidase 4; HAS2 - Hyaluronan synthase; HA – Hyaluronan; MPK-1 - nuclear MAPK phosphatase; ROS – Reactive oxygen species; EGR1 - Early growth response factor 1; SP1 – Specificity protein 1; AP-1 - Activator protein-1; α-SMA - Alpha-smooth muscle actin; PAI - plasminogen activator inhibitor.

S4. TGF-β1/CTGF signaling

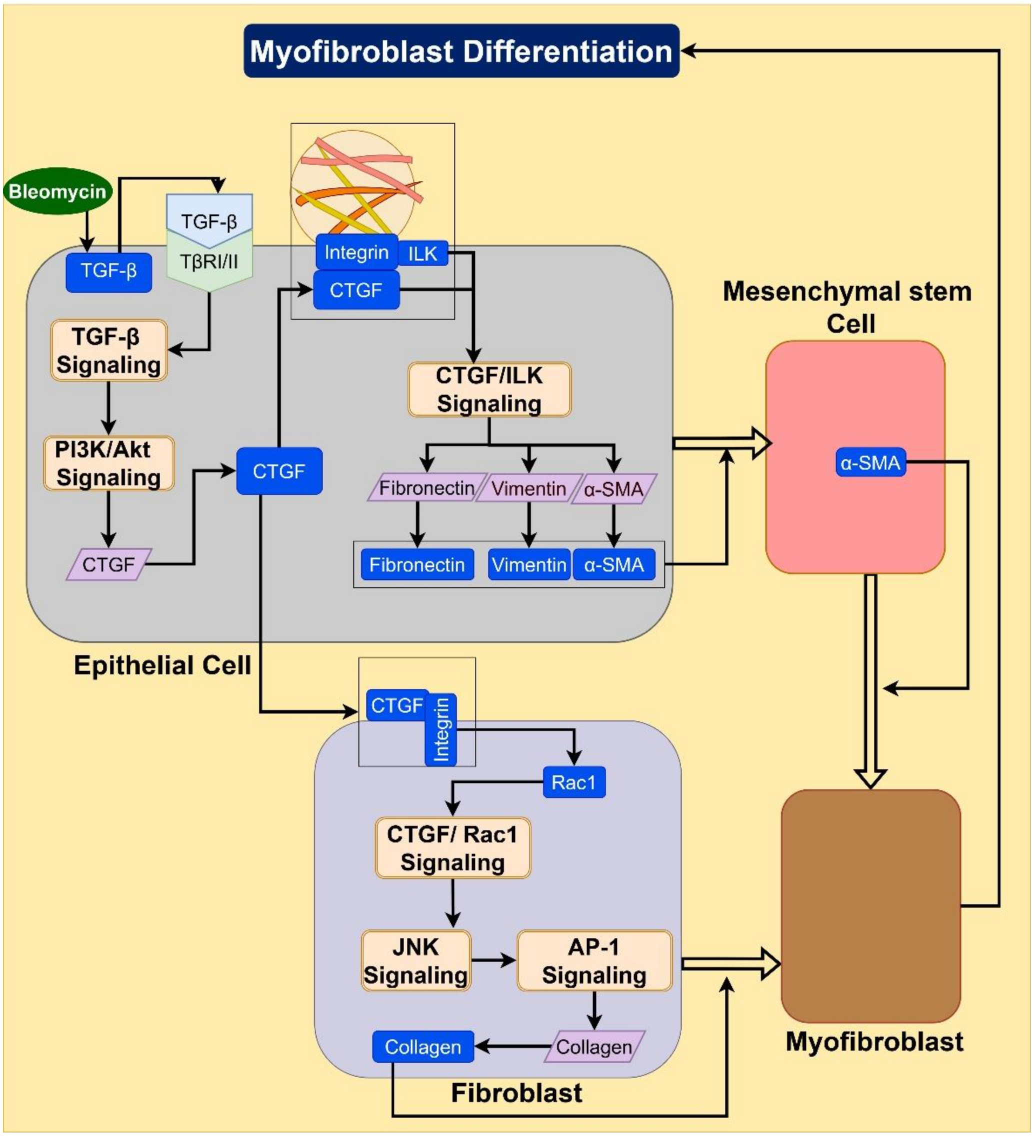

Figure S4: **TGF-β1/CTGF signaling.** TGF-β1/CTGF signaling in AECII and fibroblast promotes the EMT and FMT process, respectively, contributing to myofibroblast differentiation and pulmonary fibrosis pathogenesis [21,23,24]**.** ILK - Integrin-linked kinase; CTGF - Connective tissue growth factor.

S5. TGF-β /Sphingosine-1-phosphate (S1P) signaling

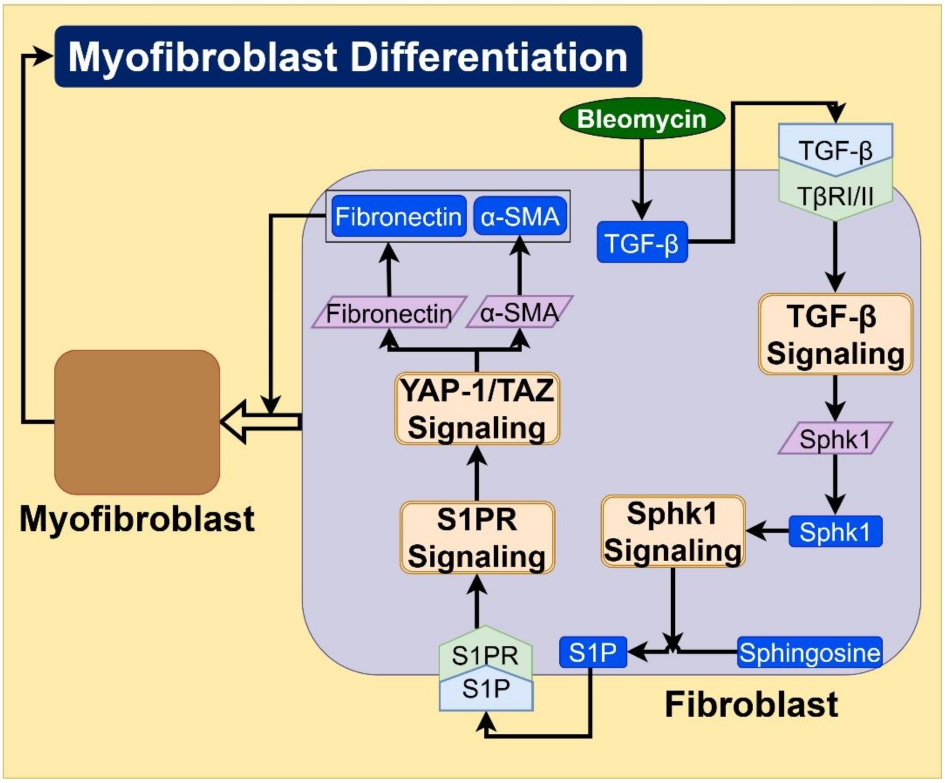

Figure S5: **TGF-β /Sphingosine-1-phosphate (S1P) signaling.** TGF-β /Sphingosine-1-phosphate (S1P) signaling promotes activation of fibroblasts, leading to myofibroblast differentiation, contributing to pulmonary fibrosis pathogenesis [25]**.** SPHK1 - Sphingosine kinase 1; S1P - Sphingosine-1 phosphate; YAP – Yes-associated protein.

S6. Thrombin/ PAR-1 signaling

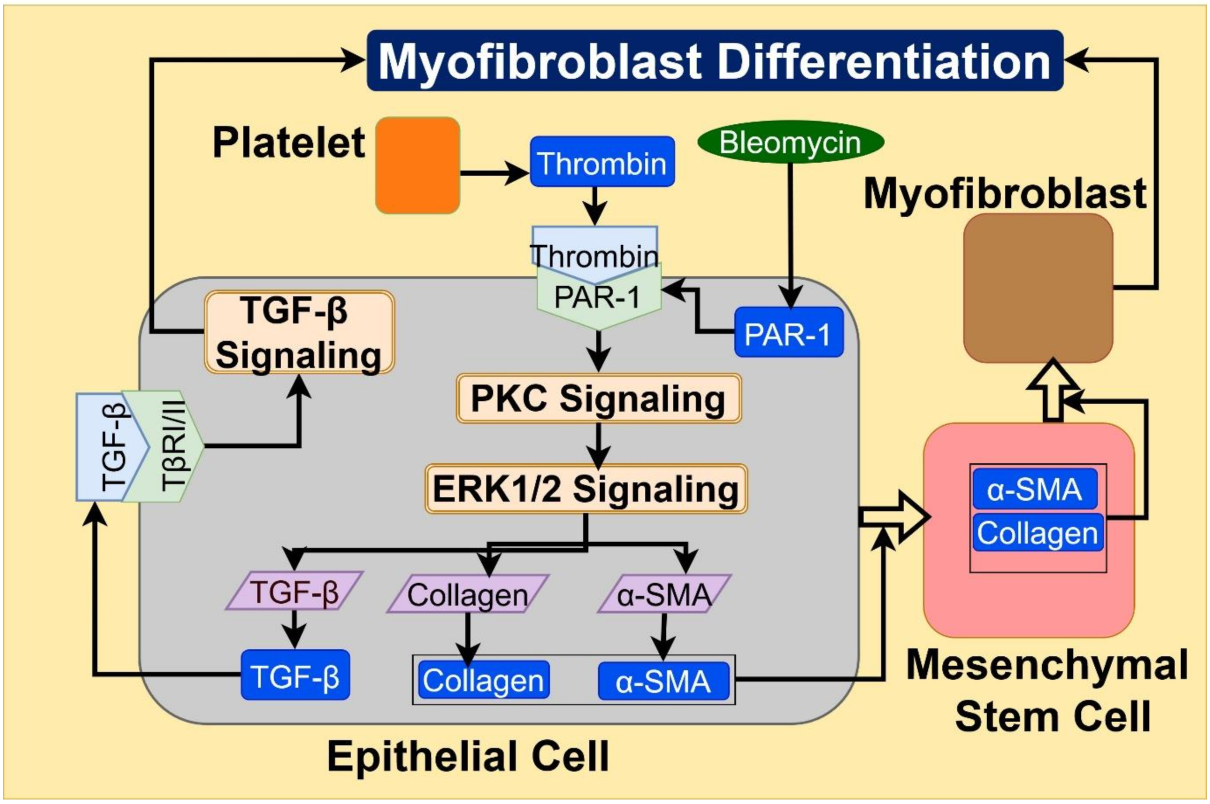

Figure S6: Thrombin/PAR-1 signaling. Thrombin/PAR-1 signaling in AECII contributes to pulmonary fibrosis pathogenesis by promoting the processes of EMT, leading to myofibroblast differentiation [28]**.** PAR-1 - Protease-activated receptor 1; PKC - Protein kinase C; ERK - Extracellular signal-regulated kinase; α-SMA - Alpha-smooth muscle actin.

S7. CXCL12/CXCR4 signaling

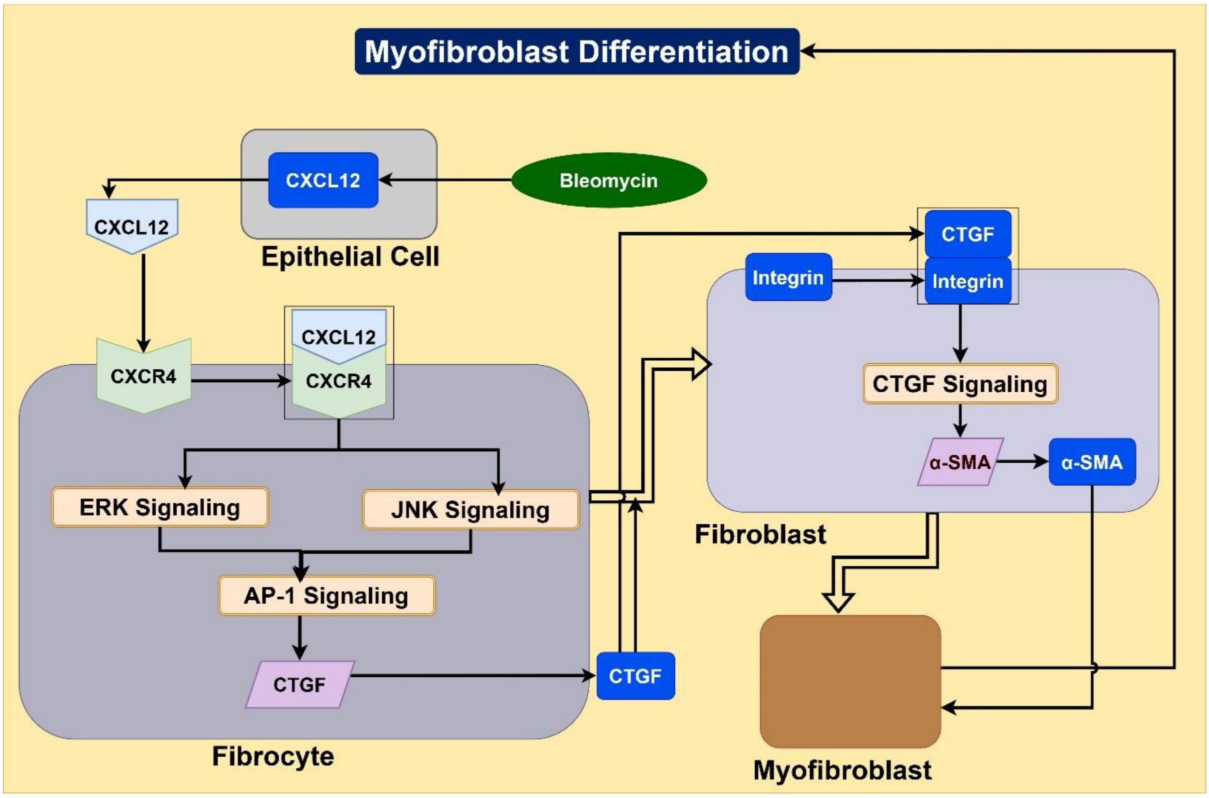

Figure S7: CXCL12/CXCR4 signaling. CXCL-12 signaling in fibrocytes contributes to pulmonary fibrosis pathogenesis by stimulating the differentiation of fibrocytes to fibroblasts, leading to myofibroblast differentiation [32]**.** JNK - c-Jun N-terminal kinase; ERK - Extracellular signal-regulated kinase; CTGF - Connective tissue growth factor; AP-1 - Activator protein-1; α-SMA - Alpha-smooth muscle actin; CXCL12 - C-X-C motif chemokine ligand 12; CXCR4 - C-X-C Motif Chemokine Receptor 4.

S8. Sonic Hedgehog signaling

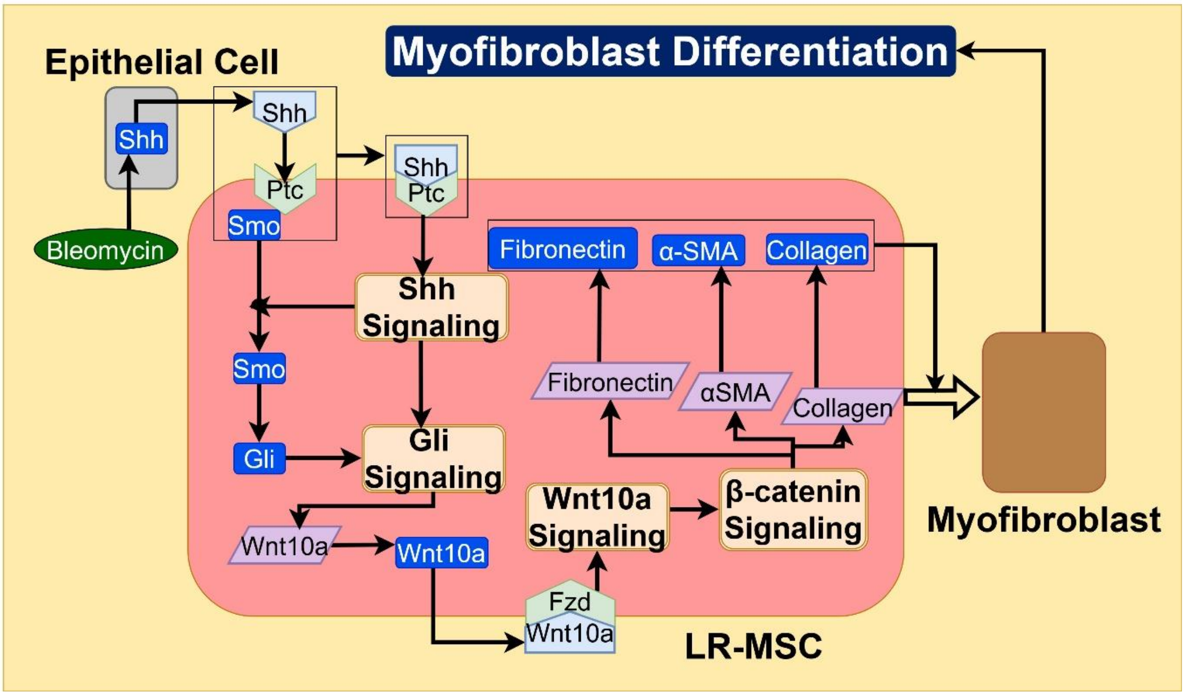

Figure S8: **Shh signaling.** Shh signaling in LR-MSC promotes the activation of Wnt10a/β-catenin signaling, leading to myofibroblast differentiation, contributing to pulmonary fibrosis pathogenesis [42,43]**.** Shh - Sonic hedgehog; Ptc - Patched receptor; Smo - Sequestered co-receptor smoothened; Gli - Glioma-associated oncogene homolog; Fzd - Frizzled10 receptor; Wnt - wingless/integrated.

S9. YAP-TAZ pathway

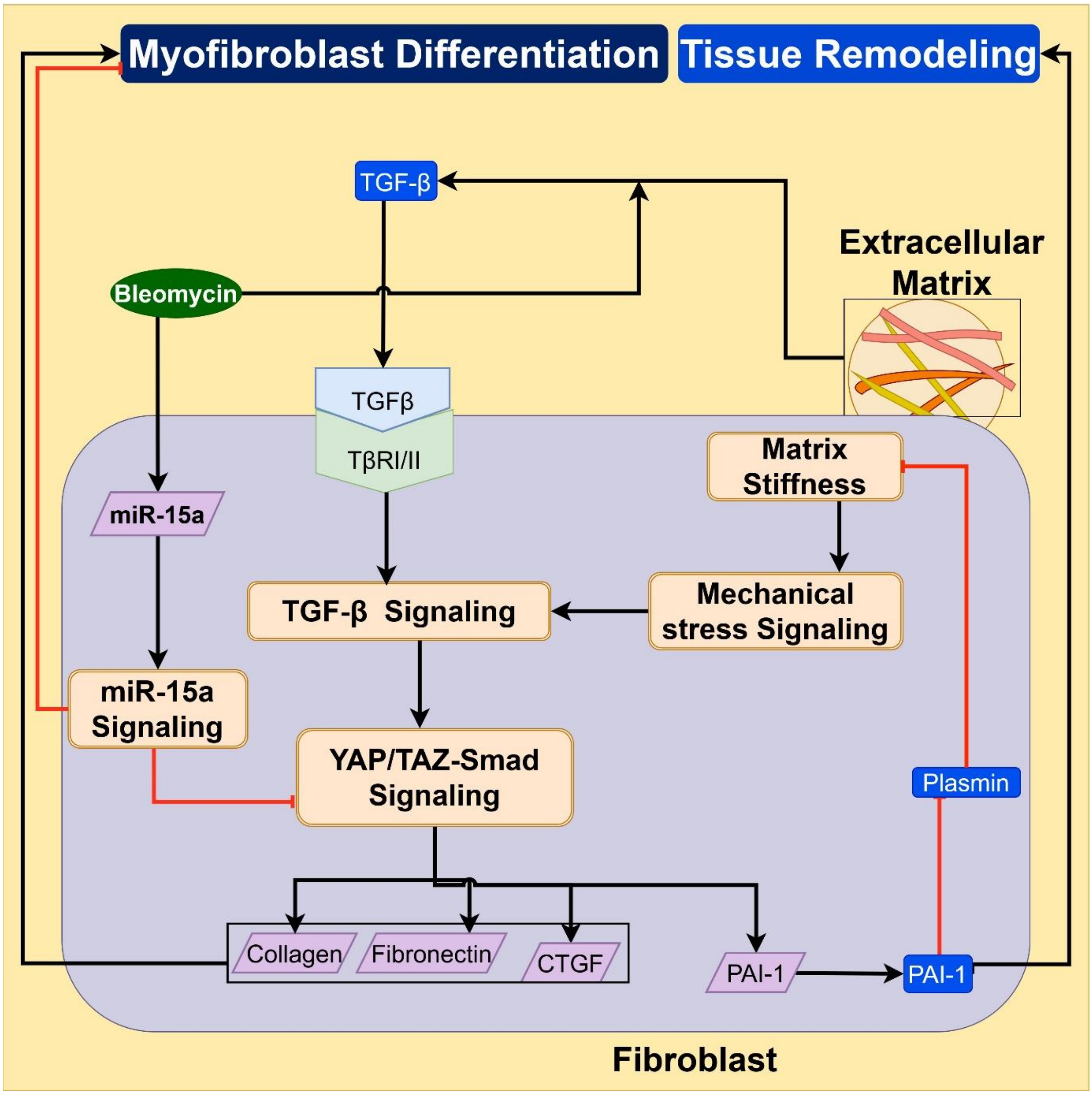

Figure S9: **TGF-β/YAP-TAZ pathway.** TGF-β/YAP-TAZ Signaling in fibroblasts contributes to pulmonary fibrosis pathogenesis by activating the fibroblast, leading to myofibroblast differentiation and consequent matrix remodeling [47,55,56]. PAI - plasminogen activator inhibitor; YAP – Yes-associated protein.

S10. TGF-β/mTORC1/ATF4 signaling axis

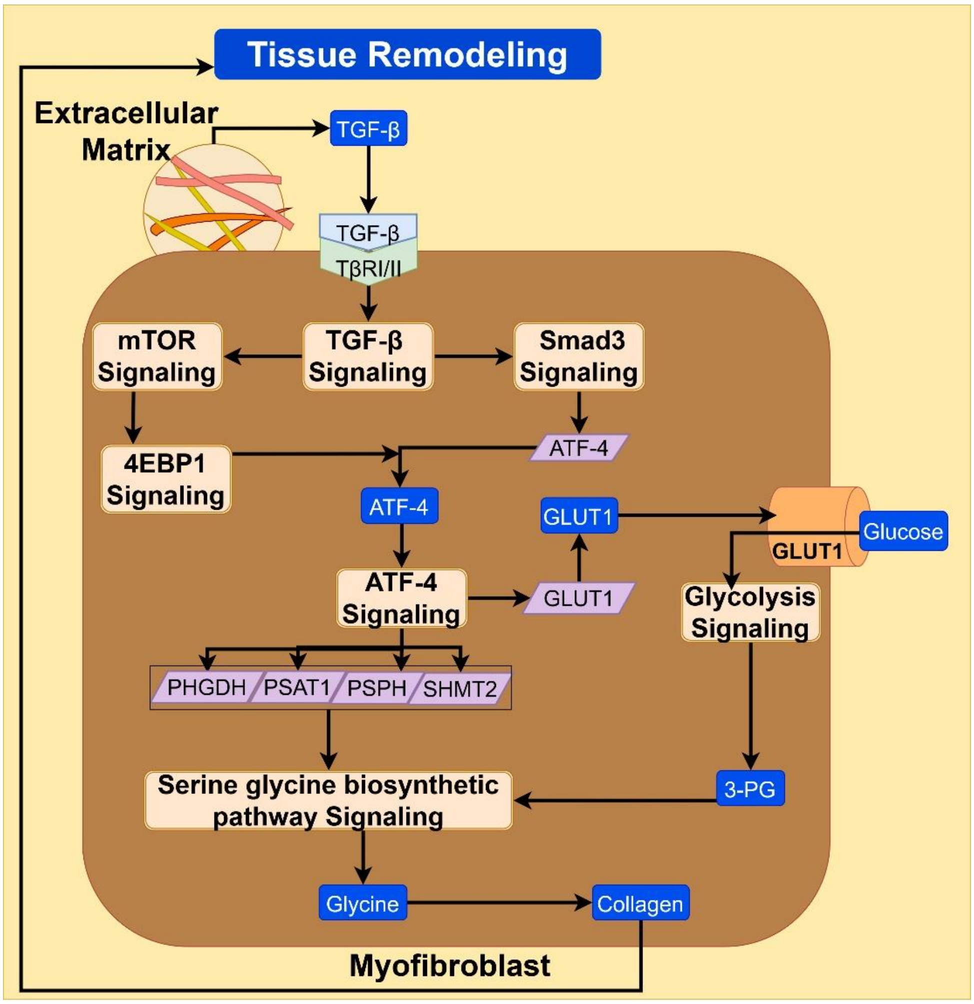

Figure S10: **TGF-β/mTORC1/ATF4 signaling axis.** TGF-β/ mTORC1/ATF4 signaling in myofibroblasts promotes the metabolic reprogramming of glycolysis as well as the serine glycine biosynthetic pathway, leading to the increased production of collagen, contributing to matrix remodeling [49]**.** mTOR - Mechanistic target of rapamycin complex 1; 4E-BP1 - 4E-binding protein 1; PHGDH - Phosphoglycerate dehydrogenase; PSAT1 - Phosphoserine, aminotransferase 1; PSPH - phosphoserine phosphatase; SHMT2 - serine hydroxy methyltransferase 2; 3-PG - 3-phosphoglycerate; GLUT1 – Glucose transporter 1.

S11. TGF-β1/Akt signaling

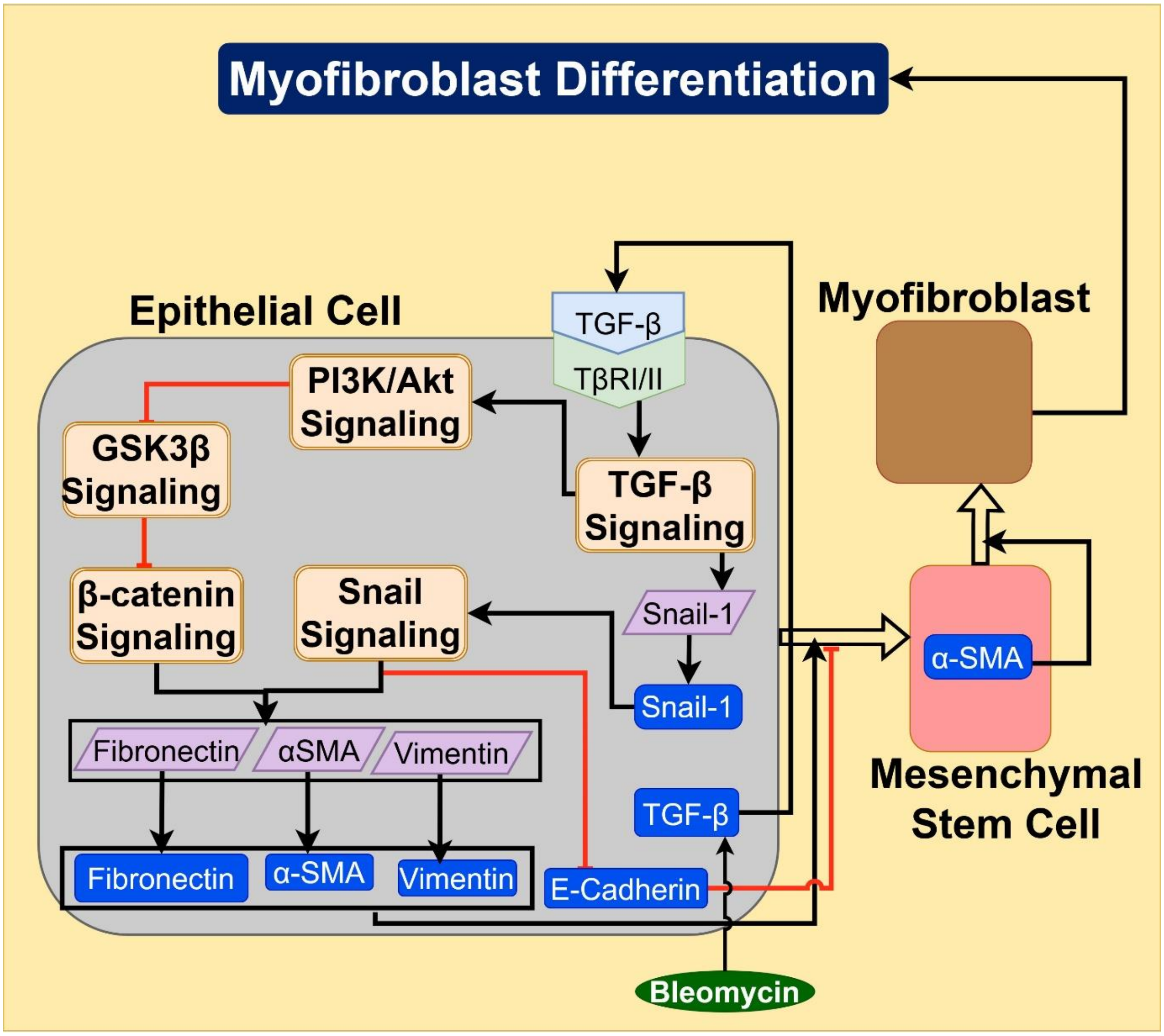

Figure S11: **TGF-β1/Akt signaling:** TGF-β1/Akt signaling in AECII promotes the differentiation of epithelial cells to mesenchymal stem cells through the processes of EMT, contributing to myofibroblast differentiation. PI3K - Phosphoinositide 3-kinase; α-SMA - Alpha-smooth muscle actin; GSK-3β - Glycogen synthase kinase-3 beta.

S12: IL-37 signaling

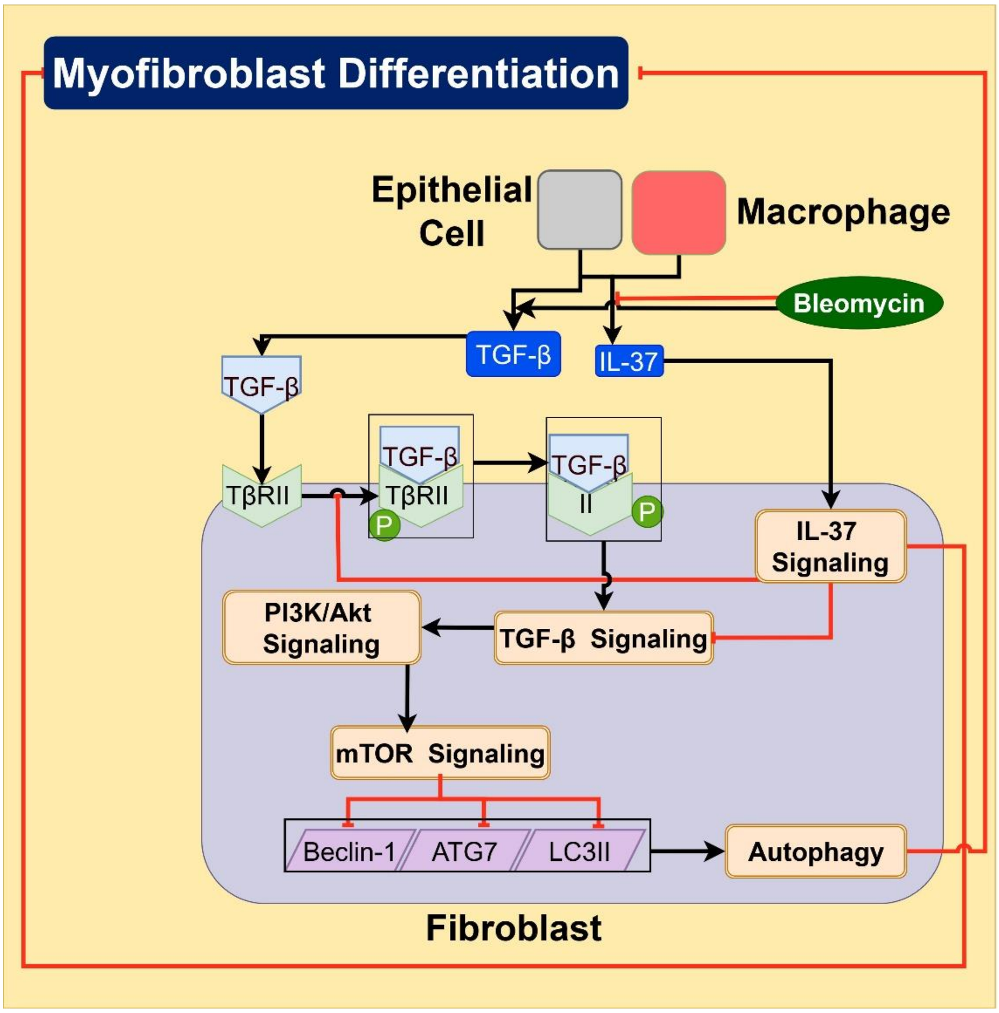

Figure S12: IL-37 signaling: IL-37 signaling in fibroblasts leads to an inhibition of TGF-β signaling and promotion of autophagy, contributing to the suppression of myofibroblast differentiation [57]. TGF-β – Transforming growth factor beta; IL-37 – Interleukin – 37; PI3K - Phosphoinositide 3-kinase; LC3 II - Lipidated form of microtubule-associated protein 1A/1B-light chain 3. Double-sided orange rectangle represents a molecular pathway within a cell; black arrow represents signal propagation; purple lozenge represents mRNA; a small dark blue rectangle represents a protein or enzyme; Dark green oval represents a small molecule; red flat arrow represents inhibition of signal propagation; light blue pentagram represents the ligand, and green step arrow represents a receptor.

S13: ROS/IL-1β signaling

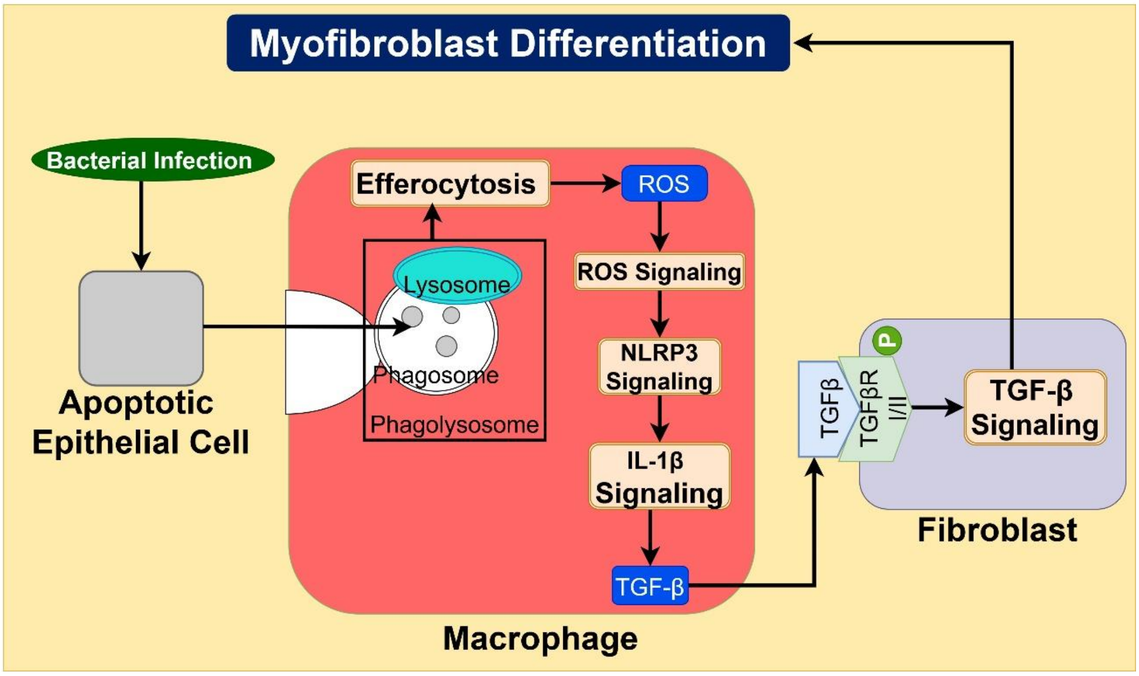

Figure S13: ROS/IL-1β signaling: ROS/ IL-1β signaling in macrophages promotes the activation of fibroblasts, leading to myofibroblast differentiation and exacerbation of pulmonary fibrosis [35]. NLRP3 - NOD-like receptor protein 3; ROS – Reactive oxygen species; IL-1β – Interleukin 1β. Double-sided orange rectangle represents a molecular pathway within a cell; black arrow represents signal propagation; purple lozenge represents mRNA; a small dark blue rectangle represents a protein or enzyme; Dark green oval represents a small molecule; light blue pentagram represents the ligand, and green step arrow represents a receptor.

S14: MMP-19 signaling

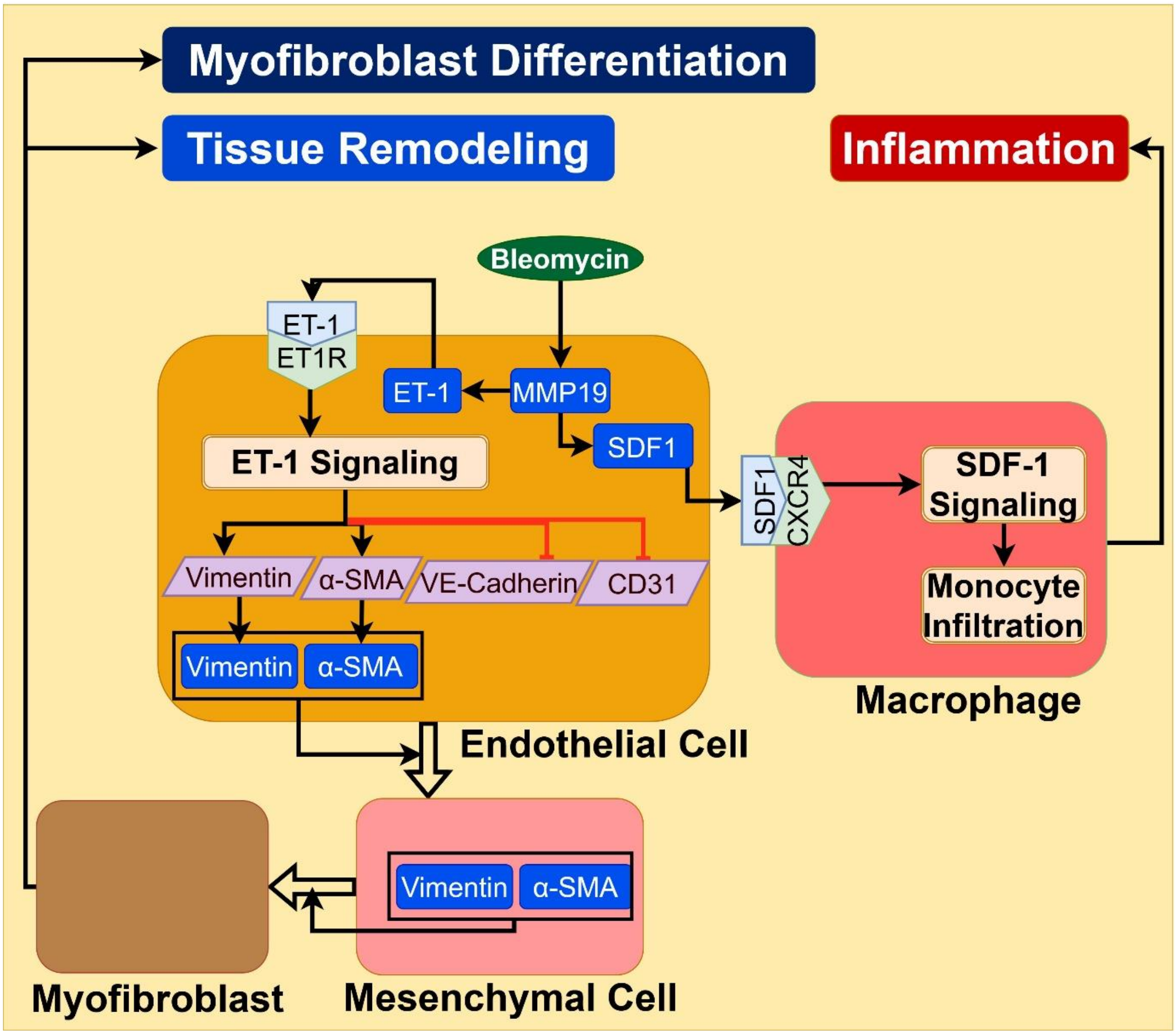

Figure S14: MMP-19 signaling: MMP-19/ET-1 signaling in endothelial cells promotes the EndMT process, leading to myofibroblast differentiation and consequent increased vascular permeability contributing to vascular tissue remodeling [58]. The MMP-19/SDF-1 signaling in macrophages promotes the recruitment of monocytes into the lung tissue, contributing to inflammation. ET-1 - Endothelin-1; MMP-19 – Matrix metalloproteinase 19. Double-sided orange rectangle represents a molecular pathway within a cell; black arrow represents signal propagation; purple lozenge represents mRNA; a small dark blue rectangle represents a protein or enzyme; Dark green oval

Supplementary Material S3

Table S1: MeSH keywords used to identify relevant literature

**MeSH Keywords**
Epithelial dysfunction AND idiopathic pulmonary fibrosis pathogenesis
Pathological mechanisms AND idiopathic pulmonary fibrosis
Role of Immune Cells AND idiopathic pulmonary fibrosis pathogenesis
Epithelial, Immune AND Inflammatory cross talk AND pulmonary fibrosis
TGF-beta 1 signaling pathways AND idiopathic pulmonary fibrosis pathogenesis
Cellular Senescence AND pulmonary fibrosis pathogenesis
Oxidative stress pathways AND pathogenesis AND idiopathic pulmonary fibrosis
Endothelial mesenchymal transition AND pathogenesis AND idiopathic pulmonary fibrosis
Alveolar macrophages AND profibrotic pathways AND pathogenesis and idiopathic pulmonary fibrosis
Pericytes AND pathogenesis AND idiopathic pulmonary fibrosis
Epithelial-mesenchymal transition pathways AND pathogenesis AND idiopathic pulmonary fibrosis
Fibroblast myofibroblast transition pathways AND pathogenesis AND idiopathic pulmonary fibrosis
Autophagy AND apoptosis pathways AND pathogenesis AND idiopathic pulmonary fibrosis
Apoptosis AND autophagy pathways AND idiopathic pulmonary fibrosis
Epithelial Inflammation pathways AND pathogenesis AND idiopathic pulmonary fibrosis
Psychological stress AND idiopathic pulmonary fibrosis
Depression AND idiopathic pulmonary fibrosis pathogenesis

Table S2. Legend of symbols used in interactome figures.

**Name**	**Symbol**	**Description**
Double-sided Orange Rectangle	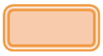	Molecular pathway
Black Arrow		Receptor/Ligand Binding, Signal propagation
Red Flat Arrow	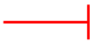	Inhibition of signal propagation
Green Cylinder	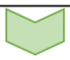	Cell surface receptor
Light Blue Pentagram	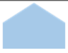	Ligand
Dark Blue Rectangle	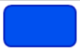	Protein/Enzyme
Pink Rectangle	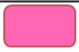	Mutated Protein/Enzyme
Dark Green Oval	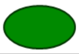	Small Molecule
Purple Lozenge	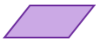	mRNA
Green Rectangle	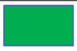	Gene

Table S3: Gene polymorphisms associated with IPF pathogenesis

Gene mutation/<br>Polymorphism	Protein (RNA) product	Familial/Sporadic IPF	Pathogenic mechanism	Reference
SFTPA1	Surfactant protein A	Familial IPF	Induce ER stress, stimulating the necroptosis of AECII.	[59].
SFTPA2 (G231V)	Surfactant protein A	Familial IPF	Triggers unfolded protein response. Associated with elevated levels of TGF-β1and two TGF-β binding proteins (LTBP-1, LTBP-4).	[59,60].
BRICHOS domain of SFTPC (L188Q)	Surfactant protein C	Familial & Sporadic IPF	Induce ER stress and UPR activation, triggering AECII apoptosis.<br>Promote inflammation by inducing IL-8 secretion.<br>Increases the susceptibility to the development of IPF, triggered by the Herpes Virus infection.	[60,61].
SP-C173T	Surfactant protein C	Familial & Sporadic IPF	Promotes influx of proinflammatory CD45+leucocytes, CD11+ monocytes, neutrophils, eosinophils, and Ly6C+ CCR2+ monocytes. Implicated in acute inflammatory exacerbations of pulmonary fibrosis.<br>Linked with impaired autophagy in AECII.	[62–64].
MUC5B promoter variant (rs35705950)	Mucin 5B	Familial & Sporadic IPF	Associated with increased expression of Muc5B protein, resulting in impaired mucociliary clearance.	[65].

RTEL1<br>(c.602delG, c.1451C > T, c.1940C > T, c.2005C > T, c.3371A > C)	Telomere elongation helicase 1	Familial IPF	Causes shortening of telomeres, leading to an impairment of DNA repair mechanisms.	(Mustafin, 2022).
TERT (c.1892G > A, c.2594G > A, c.2648T > G)	Telomere reverse transcriptase	Familial IPF	Causes shortening of telomeres, triggering cellular senescence of AECII.	[59,66,67]
TERT (rs7725218)		Sporadic IPF	Shortening of telomeres in circulating lymphocytes leads to impairment of the DNA repair mechanism.	[66,68].
PARN<br>(IVS4-2a > g, c.529C > T, c.563_564insT, c.751delA, IVS16+1g > a, c.1262A > G)	Deadenylating nuclease	Familial IPF	Shortening of telomeres in circulating lymphocytes leads to impairment of the DNA repair mechanism.	[66,69]
TERC (rs12696304)	Telomeres RNA component	Sporadic IPF	Shortening of telomeres in circulating lymphocytes leads to an impairment of the DNA repair mechanism.	[66,68,70]
TERC (r.37a > g)		Familial IPF		
TOLLIP<br>(rs111521887, rs5743894, rs5743890)	Toll-interacting protein	Sporadic IPF	Dysregulated innate immune response	[66,71]
DSP<br>(rs2076295)	Desmoplakin, an intracellular component of desmosomes, mediates cell-cell adhesion and tissue integrity.	Sporadic IPF	Decreases the DSP expression, resulting in reduced barrier integrity.<br>Increases the expression of EMT and ECM genes.	[66,72].

SFTPA1-Surfactant Protein A1; SFTPA2-Surfactant Protein A2; BRICHOS domain of SFTPC- BRICHOS domain in Surfactant Protein C; MUC5B-Mucin 5B; RTEL1-Regulator of Telomere Elongation Helicase 1; TERT-Telomerase Reverse Transcriptase; PARN-Poly(A)-Specific Ribonuclease; TERC-Telomerase RNA Component; TOLLIP-Toll Interacting Protein; DSP-DSP-Desmoplakin.

Supplementary Table S4: Therapeutic drugs for identified molecular targets.

Therapeutic agent	Molecular Target	Biological Process	Reference
Recombinant human pentraxin-2 (rhPTX-2); Pirfenidone; Nintedanib	TGF-β	Myofibroblast differentiation; tissue remodeling	[73,74].
Gancao Ganjiang decoction (GGD)	PD-1	Inflammation and myofibroblast differentiation.	[12].
2-methoxyestradiol	HIF-1α	Myofibroblast differentiation	[13].
Romilkimab, Pirfenidone	IL-4	Myofibroblast differentiation	[8,73].
QAX-576, Tralokinumab, Lebrikizumab, Romilkimab, Pirfenidone	IL-13	Myofibroblast differentiation	[8,73].
TD139	Galectin-3	Myofibroblast differentiation, tissue remodeling.	[74,75].
Pamrevlumab. Atorvastatin	CTGF	Myofibroblast differentiation	[19,22].
Pirfenidone, Scutellarin	NLRP3 inflammasome	Myofibroblast differentiation	[34–36].
GLPG1690; BBT-877	Autotaxin	Myofibroblast differentiation, tissue remodeling.	[26,74].
BMS986278;	LPA1 receptor	tissue remodeling.	[26,73].
Lonidamine	HK2	Tissue remodeling	[50].
3-PO, PFK158	PFKFB3	Tissue remodeling.	[50].
CB-839	GLS	Tissue remodeling.	[50].

TGF-β – Transforming growth factor beta; PD-1 – Programmed cell death protein 1; HIF-1α– Hypoxia-inducible factor 1-alpha; IL-4 – Interleukin 4; IL-13 – Interleukin 13; Galectin-3 – Galectin 3; CTGF – Connective tissue growth factor; NLRP3 – NLR family pyrin domain containing 3 inflammasome; LPA1 receptor – Lysophosphatidic acid receptor 1; HK2 – Hexokinase 2; PFKFB3 – 6-phosphofructo-2-kinase/fructose-2,6-biphosphatase 3; GLS – Glutaminase.

Supplementary Table S5 - PRISMA 2020 checklist.

#	Item	Status	Notes
**TITLE & ABSTRACT**			
1	Title identifies the report as a systematic review	**Met**	Title now reads "Molecular Systems Architecture of Fibrotic Lung Microenvironment in Idiopathic Pulmonary Fibrosis: A Systematic Review" – the ": A Systematic Review" clause has been inserted (shown highlighted) in this uploaded version.
2	Abstract (structured per PRISMA-for-Abstracts)	**Met**	All required elements are now present: Background/Methods/Results/Conclusion structure, databases and date range, number of studies included ("A total of 150 studies... were included in the systematic analysis"), registration status ("This systematic review was not registered"), and synthesis approach ("Findings were synthesized qualitatively into a multilayered molecular interactome rather than through statistical meta-analysis"). PRISMA 2020 Item 2 is a content requirement and is satisfied.
**INTRODUCTION**			
3	Rationale	**Met**	Introduction paragraph 1 (50 years of research, no therapeutic progress) establishes rationale well.
4	Objectives	**Partial**	This review is a mechanistic/pathway-curation systematic review with an architecture-building hypothesis (aim: assemble a multilayer molecular interactome of the fibrotic lung microenvironment). It is not a clinical-outcomes (PICO) review; therefore methods emphasize literature curation of mechanistic studies, pathway extraction, and qualitative synthesis into an interactome rather than meta-analysis of patient outcomes.
**METHODS**			
5	Eligibility criteria	**Met**	"Literature inclusion and exclusion criteria" subsection is thorough (full-text requirement, keyword requirement, exclusions for unpublished work, unrelated diseases, non-English, etc.)
6	Information sources	**Met**	PubMed/MEDLINE for search, Google Scholar for full-text retrieval only, date range April 2008–June 2025 specified. Last search date implied (June 2025) but not stated as an explicit "search conducted through [date]" – worth tightening.
7	Search strategy	**Partial**	Search strategy is described using MeSH terms and Boolean 'AND' logic, but the full reproducible database query syntax is not fully shown; therefore, reporting is partial.

#	Item	Status	Notes
8	Selection process	**Met**	Two independent reviewers (YM and PD), third-author (VAS) adjudication, screening stages described. No automation tools mentioned – fine, since none were apparently used.
9	Data collection process	**Met**	The tracked-changes text on evidence-weighting for conflicting mechanistic claims (reviewers evaluating sample size/model system/replication) directly addresses this item.
10a	Data items – outcomes	**Partial**	This review did not extract outcomes, as they were not applicable in the standard PRISMA/meta-analysis sense. For each study, we extracted mechanistic/pathway information and assessed four pre-specified criteria: hypothesis, design, methods, and sample size.
10b	Data items – other variables	**Not addressed**	An explicit list of other extracted variables was not provided. The manuscript does not clearly state all study-level characteristics captured during extraction, such as species, model system, cell type, and assay type.
11	Risk of bias assessment	**Partial**	Formal risk-of-bias scoring was not applied because this was a mechanistic/pathway-curation review; instead, studies were appraised using four pre-specified criteria: hypothesis, design, methods, and sample size.
12	Effect measures	**N/A**	Not applicable – no quantitative synthesis/meta-analysis performed. Manuscript should say so explicitly rather than leaving this silently absent.
13a–f	Synthesis methods	**Partial**	The review used a mechanistic synthesis approach by assembling pathways into a molecular architecture, connecting shared molecular species across cell-type pathways to build Figure 3. Since this is a qualitative/mechanistic synthesis rather than a quantitative meta-analysis, the PRISMA synthesis items are not fully applicable.
14	Reporting bias assessment	**Not addressed**	No formal discussion of publication bias or selective reporting risk was provided.
15	Certainty assessment (e.g., GRADE)	**N/A**	Not applicable to this review type – should be stated as such.
**RESULTS**			
16a	Study selection numbers + flow diagram	**Met**	Figure 4 (PRISMA flow diagram) and the revised Results text (1,555 → 358 duplicates removed → 1,197 screened → 483 excluded → 714 full-text → 564 excluded → 150 included) fully satisfies this and is now internally consistent.

#	Item	Status	Notes
16b	Excluded studies that might appear eligible	**Not addressed**	No separate discussion of borderline excluded studies was provided.
17	Study characteristics	**Not addressed**	Study characteristics are not presented in a separate table. Although 150 studies were included, the review focuses on building a mechanistic interactome, so study-level details such as author, year, model system, cell type, and key finding were not summarized in a standard PRISMA characteristics table.
18	Risk of bias per study	**Not addressed**	Risk of bias was not reported separately for each study. The manuscript only states the appraisal criteria used to screen studies, but does not provide study-by-study risk-of-bias results.
19	Results of individual studies	**Partial**	Individual study findings were not reported separately; instead, the relevant evidence was integrated directly into the interactome and figures, as this review is designed to build a mechanistic pathway architecture rather than provide a study-by-study results table.
20a–d	Results of syntheses	**N/A**	No statistical synthesis was performed; the “synthesis” is the interactome itself (Figures 2–3, Table 1). Should be explicitly framed as the qualitative/mechanistic equivalent.
21	Reporting bias per synthesis	**Not addressed**	A formal assessment of reporting bias per synthesis was not done, because the review is qualitative/mechanistic and not a meta-analysis.
22	Certainty of evidence	**Not addressed**	Certainty of evidence was not assessed using a formal GRADE-style tool because this review is mechanistic and pathway-curation-based rather than an outcome-focused meta-analysis. Instead, the evidence was synthesized qualitatively to build the molecular architecture.
**DISCUSSION**			
23a	General interpretation	**Met**	Discussion and Conclusion sections interpret findings well in context of prior CytoSolve architecture studies (pancreatic cancer, osteoarthritis).
23b	Limitations of included studies	**Met**	The murine/bleomycin-model translational limitation paragraph addresses this directly.
23c	Limitations of review process	**Partial**	The manuscript discusses limitations of the molecular architecture itself, such as its static nature and limited spatial resolution, but it does not clearly state the limitations of the review process.

#	Item	Status	Notes
23d	Implications for future research	**Met**	Strong – iPSC/lung-on-a-chip, spatial transcriptomics, dynamic CytoSolve simulation paragraphs all address this well.
**OTHER INFORMATION**			
24a–c	Registration and protocol	**Met**	“This review was not registered” is explicitly stated – PRISMA 2020 accepts this as compliant disclosure. No separate protocol mentioned, consistent with an unregistered review.
25	Support/funding	**Met**	“This study did not receive any external funding” is stated in Declarations.
26	Competing interests	**Met**	All three authors’ CytoSolve, Inc. employment is disclosed.
27	Availability of data/materials	**Met**	Stated that supporting data are within the article and supplementary materials.

Abbreviations: Met = fully reported; · Partial = incomplete/implicit; · Not addressed = no statement given; · N/A = not applicable to this review type.

Supplementary Material S4

CytoSolve® Operating Guide Protocol Summary

S1. CytoSolve® System

CytoSolve is a well-established computational systems biology framework of technology and processes that provides the capability to derive molecular mechanisms of action; to create quantitative and predictive models of those mechanisms; and, to employ the resultant models to simulate complex biomolecular phenomena [76–80]. In neurovascular studies, the CytoSolve framework elicited and derived a multi-layered engineering molecular systems architecture integrating the anatomy of the neurovascular unit, molecular mechanisms, and disease to demonstrate the commonality of multiple neurovascular diseases as communication dysfunctions in common molecular signaling sub-systems and compounds [79].

In oncology, CytoSolve's capability has been employed for the *in silico* modeling of pancreatic cancer to identify and optimize a multi-combination therapeutic that was subsequently allowed for clinical trials by the United States Food and Drug Administration [81], has been used to identify the molecular systems architecture of interactome in acute myeloid leukemia (AML) microenvironment [82], and has been independently recognized by leading cancer researchers as a platform for developing multi-combination therapies [78]. In cardiovascular research, CytoSolve has been used to accurately model the release of nitric oxide (NO) production in endothelial cells subjected to shear stress [80].

In the area of plant biology, CytoSolve enabled the quantitative molecular systems understanding of C1 metabolism - a critical system of molecular pathways inherent to all plants, fungi and bacteria - to understand the systemic effects oxidative stress and genetic modification on C1 metabolism in soy [83–86]. Recently, CytoSolve was used to discover and model the mechanisms of immunomodulatory effect of bioactive compound in green tea on organ transplant tolerance [87], and elucidate effect of bioactive compounds from fruit, berry, vegetable (FBV) juice power on low grade chronic inflammation [88].

S2. CytoSolve® System Capabilities

The method used in this study provides a scalable computational framework for modeling large-scale biological systems by dynamic integration of an ensemble of multiple molecular pathway models [76]. This method enables the development of large-scale models of complex biological systems that span multiple temporal and spatial scales as well as across diverse domains. Rather than attempting to monolithically model systems of biochemical reactions, a distributed engineering systems approach – a relatively novel concept in systems biology– is employed that breaks a large scale biological system into an ensemble of smaller molecular pathway models that are computationally coupled. This approach makes the modeling of large-scale biological systems both tractable and scalable.

S3. Key Elements of CytoSolve® System Protocol

There are six (6) steps that comprise the protocol to use the CytoSolve® system. Fig. A1 illustrates those steps of the protocol.

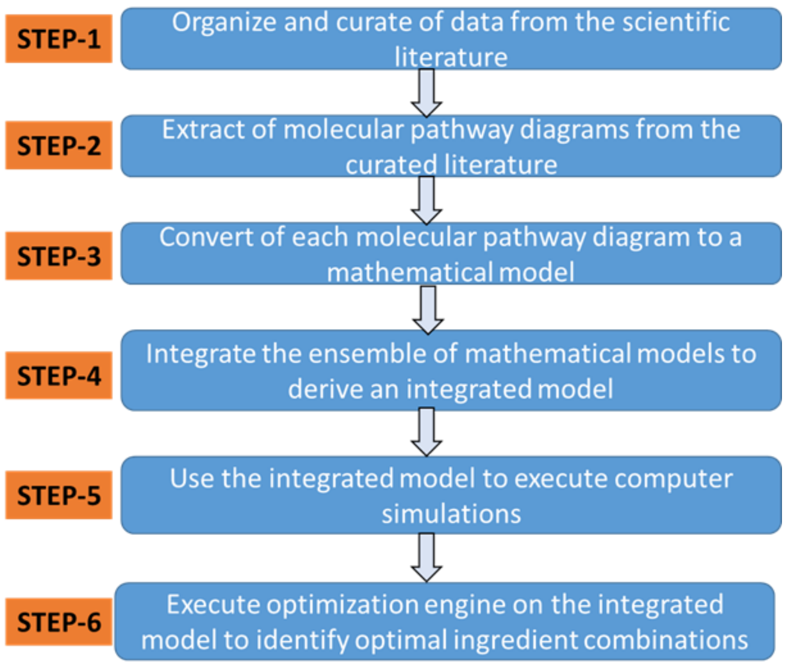

Figure S15. CytoSolve® Protocol Overview. The six steps involved in the CytoSolve® protocol. Steps 1 and 2 relate to performing systematic literature review to identify molecular pathways and the biochemical parameters required for computational modeling. Steps 3 to 5 relate to construction of individual models, integration of individual models, and executing simulations using the integrated modes. Step 6 provides the option to employ CytoSolve® optimization engine to discover the optimal combination of inputs (ingredients/compounds) for a specific range of values of outputs (biomarkers associated with particular biomolecular functions).

The six (6) steps are listed below:

1) Organize and curate data from the scientific literature (§S4)

2) Extract molecular pathway diagrams from the curated literature (§S5)

3) Convert each molecular pathway diagram to a mathematical model (§S6)

4) Integrate individual mathematical models to derive an integrated model (§S7)

5) Use the integrated model to execute computer simulations to analyze the effect of ingredients on interest, individually as well as in combination (§S8)

6) Execute optimization engine on the integrated model to identify optimal ingredient combination (§S9)

S4. CytoSolve® Protocol for Organization and Curation of Data from the Scientific Literature

This protocol step is the first step of the overall CytoSolve® Protocol indicated in Fig.A1. In this step, the scientific literature is searched to identify journal papers that contain research on the area of interest. For a particular area of interest, and *ingredients of interest*, molecular pathways for the area of interest, and the effect of ingredients of interest on those molecular pathways are identified and organized.

Four (4) specific steps are executed per the CytoSolve® Protocol to organize and curate the journal papers, as itemized below:

i. Create a list of Medical Subject Headings (MeSH) *keywords* to optimize recall and precision of peer-reviewed articles. The keywords are typically a list of words in combination with Boolean operators i.e. “AND”, “OR”, etc.
ii. Search and retrieve the relevant peer-reviewed articles published during a specific *time period* from PubMed, Medline, and Google Scholar. These set of articles are stored as an “Initial Set” repository. The time period is a date range i.e. “January 1989 to April 2023,” etc.

iii. Screen the titles and abstracts of articles in the Initial Set repository to identify most relevant articles based on our inclusion criteria. These set of articles are stored as the “Final Set” repository

iv. Perform full-length review of peer-reviewed articles from the Final Set repository

Abstracts and unpublished literature were not sought as they have not been peer reviewed adequately to authenticate their results.

Articles obtained from the systematic literature review are categorized into three categories: 1) Articles related to molecular pathways implicated in the area of interest; 2) articles related to interactions of ingredients of interest with molecular targets in area of interest pathways; and, 3) articles related to pharmacokinetic properties of the ingredients of interest.

S5. CytoSolve® Protocol for Extraction of Data from the Scientific Literature

Journal articles in Group 1 are reviewed to gather data relevant to molecular pathways involved in the area of interest. The steps to extract and represent molecular pathways diagrammatically are itemized below:

1. Identify and extract:

    a. chemical species involved in the molecular pathways

    b. types of cells

c. cellular components (e.g. cytosol, mitochondria, nucleus, etc.) where the chemical species are present in each cell type

2. Identify and diagrammatically represent biochemical interactions

3. Interconnect biochemical reactions to create molecular pathway diagram in each cell type

Journal articles in Group 2 are reviewed to gather data relevant to pharmacokinetics of ingredients of interest.

Journal articles in Group 3 are reviewed to extract following information:

1. Reaction rate constants of biochemical reactions involved in molecular pathways
2. Molecular targets of ingredients of interest in the molecular pathways of interest

The kinetic parameters used in this study are derived using principles of Michaelis-Menten kinetics that are based on the steady-state approximation of the biochemical reactions [89,90].

S6. CytoSolve® Protocol for Setup of Individual In Silico Mathematical Models

The steps to convert molecular pathway diagrams to mathematical models are itemized below:

1. Convert biochemical reactions involved in each of the molecular pathway into ordinary differential equations (mathematical expressions that describe the rate of change)

2. Represent each molecular pathway as a system of ordinary differential equations

3. Encode the system of differential equations in a computer software source code format known as Systems Biology Markup Language (SBML)[91] to construct a mathematical model for a particular molecular pathway

4. Store each model as a separate SBML file

S7. CytoSolve® Protocol for Integration of Individual In Silico Mathematical Models

In order to create an integrative quantitative model of the area of interest, it is necessary to mathematically couple the solutions across the ensemble of individual molecular pathway models. Such mathematical coupling is performed using the CytoSolve [77,87,92] computational engine, which is described in detail in Ayyadurai and Dewey, 2011[76] and Ayyadurai et al., 2022. The computational architecture of CytoSolve enables the integration of plurality of molecular pathway models [76,92].

The steps to integrate the ensemble of individual mathematical models are listed below:

1. Upload individual SBML files, constructed in §A6 of Appendix A, to CytoSolve engine

2. Update the initial conditions for the molecular species in all the mathematical models in the graphical user interface

3. Update simulation period was specified in the graphical user interface

4. Review and confirm molecular species and reaction duplicates across all the individual models in the graphical user interface

5. Commence integration of individual models

For a computational system biology analysis wherein the simulation of biochemical reactions is being executed and the governing equations are well known, as is in this case, the error bounds are set prior to executing simulations [93,94]. Herein, the error bounds are set to 10^{-6} prior to the execution of simulations. This means that the solutions to the governing equations used in the simulation of specific biochemical reactions must be within these error bounds. Therefore, *in silico* – computational – results from such simulations will not have error bars, which customarily appear in results reported from *in vitro* and *in vivo* experimental studies [95].

S8. CytoSolve® Protocol for Simulation of Integrated In Silico Mathematical Model

The following steps are performed to execute the computer simulations:

1. Input biochemical reactions for interaction between ingredients of interest and molecular pathways involved in area of interest

2. Input the kinetic rate constants for each of the biochemical reaction

3. Input the initial concentrations for each of the molecular species in the biochemical reactions

4. Input the time period for the simulation of integrative models, and dose levels of ingredients of interest

5. Execute the integrative model under control conditions

6. Execute the integrative model in presence of ingredients of interest individually

7. Execute the integrative model in presence of the combination of ingredients

The steps for analyzing simulation output data are itemized below:

1. Export the raw data to Microsoft Excel

2. Extract the steady state levels of biomarkers

3. Plot steady state levels of biomarkers as a function of simulation time in presence and absence of ingredients of interest, individually as well as in combination.

S9. CytoSolve® Protocol to Execute Optimization Engine on the Integrated Model to Identify Optimal Ingredient Combination

The following steps are performed to execute the optimization engine:

1. Simulate the integrative model in presence of ingredients of interest

2. Select "Optimize Results" on the CytoSolve® graphical user interface to initiate the optimization engine

3. In the optimization engine, choose "Objective" for the ingredients of interest and the biomarkers affected by the ingredients of interest. The objective is set to "optimize" for the ingredients of interest; "maximize" for the biomarkers that are related to positive outcome; and, "minimize" for the biomarkers that are related to negative outcomes.

4. Execute the optimization engine and record the results

The steps for analyzing simulation output data are itemized below:

1. Export the raw data to Microsoft Excel

2. Extract the values of ingredients of interest that have either minimized or maximized the biomarker

S10. CytoSolve® System Constraints

Although the framework developed in this study provides a detailed mechanistic understanding of ALS that match well with published clinical data, some of the model components' parameters were derived from experiments using different cell types, as well as different experimental conditions such as variations in culture conditions that adds to the uncertainty of the model predictions [96]. Such issues of parameter estimation, however, are not unique to this study. They are common to a number of cellular mathematical models [96] and do warrant further experimental investigation and validation.

Supplementary Material S5 - Glossary – Main Manuscript and Supplemental Figures

Figure 1. The Fibrotic Lung Microenvironment in Idiopathic Pulmonary Fibrosis (IPF).
Abbreviations: AECI, Alveolar epithelial cell type I; AECII, Alveolar epithelial cell type II; EMT, Epithelial-mesenchymal transition; PMT, Pericyte-myofibroblast transition; EndMT, Endothelial-mesenchymal transition; FMT, Fibroblast-myofibroblast transition; ECM, Extracellular matrix.

Figure 2. Molecular systems architecture of lung microenvironment signaling in pulmonary fibrosis.
Abbreviations: TLR, Toll-like receptor; IFN-γ, Interferon gamma; PD-1, Programmed cell death protein 1; MMP-19, Matrix metalloproteinase 19; LPA, Lysophosphatidic acid; TGF-β, Transforming growth factor beta; PAR-1, Protease-activated receptor 1; IL, Interleukin; CCL18, C-C motif chemokine ligand 18; NLRP3, NOD-like receptor protein 3; HIF-1α, Hypoxia inducible factor-1 subunit alpha; FAK, Focal adhesion kinase; Shh, Sonic hedgehog; MSC, Mesenchymal stem cell; ILC2, Type 2 innate lymphoid cell; RTEL1, Telomere

elongation helicase 1; TERT, Telomerase reverse transcriptase; MUC5B, Mucin 5B; SFTPC, Surfactant protein C; SFTA, Surfactant protein A; PARN, Poly(A)-specific ribonuclease; TOLLIP, Toll-interacting protein; DSP, Desmoplakin.

Figure 3. Interactome of the molecular systems architecture of the lung microenvironment involved in pulmonary fibrosis pathogenesis.

Abbreviations: TLR, Toll-like receptor; TGF-β, Transforming growth factor beta; LTBP, Latent TGF-β-binding protein; LAP, Latency-associated peptide; ILK, Integrin-linked kinase; MAPK, Mitogen-activated protein kinase; LPA, Lysophosphatidic acid; ERK, Extracellular signal-regulated kinase; PKC, Protein kinase C; PAI, Plasminogen activator inhibitor; CXCL, C-X-C motif chemokine ligand; PI3K, Phosphoinositide 3-kinase; Shh, Sonic hedgehog; MMP, Matrix metalloproteinase; NLRP3, NOD-like receptor protein 3; PAR, Protease-activated receptor; IL, Interleukin; DKK-1, Dickkopf-1; HMGB1, High mobility group box 1; HIF-1α, Hypoxia inducible factor-1 subunit alpha; VEGF, Vascular endothelial growth factor; FGF, Fibroblast growth factor; PDGF, Platelet-derived growth factor; ROS, Reactive oxygen species; PHD2, Prolyl hydroxylase domain protein 2; NOX, NADPH oxidase; JNK, c-Jun N-terminal kinase; IL-33, Interleukin-33; α-SMA, Alpha-smooth muscle actin; SDF-1, Stromal cell-derived factor 1; Cd, Cadmium; CB, Carbon black; SP1, Specificity protein 1; PAD2, Peptidyl arginine deiminase 2; Cit-Vim, Citrullinated vimentin; MCP-1, Monocyte chemoattractant protein-1; CTGF, Connective tissue growth factor; CCR2, C-C chemokine receptor type 2; Ly6+, Lymphocyte antigen 6; CD206, Cluster of differentiation 206; iNOS, Inducible nitric oxide synthase; CIITA, Class II major histocompatibility complex transactivator; YKL-40, Chitinase 3-like 1; PD-L1, Programmed death-ligand 1; SHP-2, Src homology-2 domain-containing phosphatase 2; RORγt, Retinoid orphan receptor gamma t; STAT3, Signal transducer and activator of transcription 3; S1PR2, Sphingosine-1-phosphate receptor 2; Arg-1, Arginase-1; AP-1, Activator protein-1; CXCR, C-X-C motif chemokine receptor; ROCK, Rho-associated coiled-coil containing protein kinase; GSK-3β, Glycogen synthase kinase-3 beta; HA, Hyaluronan; SPHK1, Sphingosine kinase 1; S1P, Sphingosine-1-phosphate; YAP, Yes-associated protein; LR-MSC, Lung-resident mesenchymal stem cell; Ptc, Patched receptor; Smo, Smoothened; Gli, Glioma-associated oncogene homolog; Fzd, Frizzled receptor; Wnt, Wingless/integrated; LPA1/LPA2, Lysophosphatidic acid receptor 1/2; IGFBP-3, Insulin-like growth factor-binding protein 3; PDGFRβ, Platelet-derived growth factor receptor beta; MKL1, Megakaryoblastic leukemia 1; mTORC1, Mechanistic target of rapamycin complex 1; 4E-BP1, 4E-binding protein 1; 3-PG, 3-phosphoglycerate; GLUT1, Glucose transporter 1; GLS, Glutaminase; GDH, Glutamate dehydrogenase; ET-1, Endothelin-1.

Figure 5. TLR4 signaling.

Abbreviations: HMGB1, High mobility group box 1; TLR, Toll-like receptor; eCIRP, Extracellular cold-inducible RNA-binding protein; LPS, Lipopolysaccharide; TNF-α, Tumor necrosis factor alpha; Cd, Cadmium; CB, Carbon black; SP1, Specificity protein 1; PAD2, Peptidyl arginine deiminase 2; Cit-Vim, Citrullinated vimentin; MCP-1, Monocyte chemoattractant protein-1; CTGF, Connective tissue growth factor; PI3K, Phosphoinositide 3-kinase; IL, Interleukin; CD26, Cluster of differentiation 26.

Figure 6. IFN-γ and IL-4 signaling in monocytes.

Abbreviations: MCP-1, Monocyte chemoattractant protein-1; CCR2, C-C chemokine receptor type 2; Ly6+, Lymphocyte antigen 6; TLR, Toll-like receptor; CCL18, C-C motif chemokine ligand 18; SP1, Specificity protein 1; STAT1, Signal transducer and activator of transcription 1; CD206, Cluster of differentiation 206; ERK, Extracellular signal-regulated kinase; JAK, Janus kinase; IRF-3, Interferon regulatory factor 3; iNOS, Inducible nitric oxide synthase; IFN-γ, Interferon gamma; CIITA, Class II major histocompatibility complex transactivator; TGF-β, Transforming growth factor beta; YKL-40, Chitinase 3-like 1.

Figure 7. TGF-β/PD-1–IL-6 signaling.

Abbreviations: PD-1, Programmed cell death protein 1; IL, Interleukin; SHP-2, Src homology-2 domain-containing phosphatase 2; RORγt, Retinoid orphan receptor gamma t; STAT3, Signal transducer and activator of transcription 3; PD-L1, Programmed death-ligand 1.

Figure 8. TGF-β1/Smad signaling.

Abbreviations: TGF-β, Transforming growth factor beta; LTBP, Latent TGF-β-binding protein; LAP, Latency-associated peptide; α-SMA, Alpha-smooth muscle actin; LPA, Lysophosphatidic acid; LPA2, Lysophosphatidic acid receptor 2; PAR-1, Protease-activated receptor 1; ROCK, Rho-associated coiled-coil containing protein kinase; CTGF, Connective tissue growth factor; MMP, Matrix metalloproteinase; PAI, Plasminogen activator inhibitor.

Figure 9. NLRP3 signaling.

Abbreviations: NLRP3, NOD-like receptor protein 3; TLR, Toll-like receptor; α-SMA, Alpha-smooth muscle actin; MMP, Matrix metalloproteinase; DKK-1, Dickkopf-1; MAPK, Mitogen-activated protein kinase.

Figure 10. LPA signaling.

Abbreviations: LPA, Lysophosphatidic acid; LPA1, Lysophosphatidic acid receptor 1; LPA2, Lysophosphatidic acid receptor 2; ROCK, Rho-associated coiled-coil containing protein kinase; α-SMA, Alpha-smooth muscle actin; PI3K, Phosphoinositide 3-kinase; ERK, Extracellular signal-regulated kinase.

Figure 11. Galectin/Akt signaling.

Abbreviations: PI3K, Phosphoinositide 3-kinase; α-SMA, Alpha-smooth muscle actin; GSK-3β, Glycogen synthase kinase-3 beta.

Figure 12. HIF-1α/NOX4 signaling.

Abbreviations: HIF-1α, Hypoxia inducible factor-1 subunit alpha; NOX, NADPH oxidase; IGFBP-3, Insulin-like growth factor-binding protein 3; PHD2, Prolyl hydroxylase 2.

Figure 13. Notch1/PDGFβ–TGF-β/YAP-TAZ-MKL-1 signaling axis.

Abbreviations: PDGFRβ, Platelet-derived growth factor receptor beta; MKL1, Megakaryoblastic leukemia 1; FAK, Focal adhesion kinase.

Figure 14. TGF-β/Smad3-p38 MAPK signaling axis.

Abbreviations: GLS, Glutaminase; GDH, Glutamate dehydrogenase; P5CS, Pyrroline-5-carboxylate synthase; PSAT1, Phosphoserine aminotransferase.

Figure 15. IL-11 signaling.

Abbreviations: COL1A1, Collagen type I alpha 1; ERK, Extracellular signal-regulated kinase; STAT3, Signal transducer and activator of transcription 3; JAK, Janus kinase; VEGF, Vascular endothelial growth factor; CTGF, Connective tissue growth factor; ET-1, Endothelin-1; FGF, Fibroblast growth factor; PDGF, Platelet-derived growth factor.

References

1. Rajesh R, Atallah R, Bärnthaler T. Dysregulation of metabolic pathways in pulmonary fibrosis. Pharmacol Ther. 2023;246. https://doi.org/10.1016/j.pharmthera.2023.108436

2. Roque W, Romero F. Cellular metabolomics of pulmonary fibrosis, from amino acids to lipids. Am J Physiol - Cell Physiol. 2021;320:C689–95. https://doi.org/10.1152/ajpcell.00586.2020

3. Mora AL, Torres-González E, Rojas M, Corredor C, Ritzenthaler J, Xu J, et al. Activation of alveolar macrophages via the alternative pathway in herpesvirus-induced lung fibrosis. Am J Respir Cell Mol Biol. 2006;35:466–73. https://doi.org/10.1165/rcmb.2006-0121OC

4. Liu S, Liu C, Wang Q, Liu S, Min J. CC Chemokines in Idiopathic Pulmonary Fibrosis: Pathogenic Role and Therapeutic Potential. Biomolecules. 2023;13:1–20. https://doi.org/10.3390/biom13020333

5. Luzina IG, Tsymbalyuk N, Choi J, Hasday JD, Atamas SP. CCL18-stimulated upregulation of collagen production in lung fibroblasts requires Sp1 signaling and basal Smad3 activity. J Cell Physiol. 2006;206:221–8. https://doi.org/10.1002/jcp.20452

6. Zhang L, Wang Y, Wu G, Xiong W, Gu W, Wang CY. Macrophages: Friend or foe in idiopathic pulmonary fibrosis? Respir Res. Respiratory Research; 2018;19:1–10. https://doi.org/10.1186/s12931-018-0864-2

7. Li D, Guabiraba R, Besnard AG, Komai-Koma M, Jabir MS, Zhang L, et al. IL-33

promotes ST2-dependent lung fibrosis by the induction of alternatively activated macrophages and innate lymphoid cells in mice. J Allergy Clin Immunol. 2014;134:1422-1432.e11. https://doi.org/10.1016/j.jaci.2014.05.011

8. She YX, Yu QY, Tang XX. Role of interleukins in the pathogenesis of pulmonary fibrosis. Cell Death Discov. 2021;7. https://doi.org/10.1038/s41420-021-00437-9

9. Zhao J, Okamoto Y, Asano Y, Ishimaru K, Aki S, Yoshioka K, et al. Sphingosine-1-phosphate receptor-2 facilitates pulmonary fibrosis through potentiating IL-13 pathway in macrophages. PLoS One. 2018;13:1–22. https://doi.org/10.1371/journal.pone.0197604

10. Celada LJ, Kropski JA, Herazo-Maya JD, Luo W, Creecy A, Abad AT, et al. PD-1 up-regulation on CD4+ T cells promotes pulmonary fibrosis through STAT3-mediated IL-17A and TGF-β1 production. Sci Transl Med. 2018;10. https://doi.org/10.1126/scitranslmed.aar8356

11. Guo X, Sunil C, Adeyanju O, Parker A, Huang S, Ikebe M, et al. PD-L1 mediates lung fibroblast to myofibroblast transition through Smad3 and β-catenin signaling pathways. Sci Rep [Internet]. Nature Publishing Group UK; 2022;12:1–14. https://doi.org/10.1038/s41598-022-07044-3

12. Wang D, Gong L, Li Z, Chen H, Xu M, Rong R, et al. Antifibrotic effect of Gancao Ganjiang decoction is mediated by PD-1 / TGF-β1 / IL-17A pathway in bleomycin-induced idiopathic pulmonary fibrosis. J Ethnopharmacol [Internet]. Elsevier B.V.; 2021;281:114522. https://doi.org/10.1016/j.jep.2021.114522

13. Jarman ER, Khambata VS, Cope C, Jones P, Roger J, Ye LY, et al. An Inhibitor of NADPH oxidase-4 attenuates established pulmonary fibrosis in a rodent disease model. Am J Respir Cell Mol Biol. 2014;50:158–69. https://doi.org/10.1165/rcmb.2013-0174OC

14. Ismail S, Sturrock A, Wu P, Cahill B, Norman K, Huecksteadt T, et al. NOX4 mediates hypoxia-induced proliferation of human pulmonary artery smooth muscle cells: The role of

autocrine production of transforming growth factor-β1 and insulin-like growth factor binding protein-3. Am J Physiol - Lung Cell Mol Physiol. 2009;296. https://doi.org/10.1152/ajplung.90488.2008

15. Liu RM, Choi J, Wu JH, Pravia KAG, Lewis KM, Brand JD, et al. Oxidative modification of nuclear mitogen-activated protein kinase phosphatase 1 is involved in transforming growth factor β1-induced expression of plasminogen activator inhibitor 1 in fibroblasts. J Biol Chem. 2010;285:16239–47. https://doi.org/10.1074/jbc.M110.111732

16. Amara N, Goven D, Prost F, Muloway R, Crestani B, Boczkowski J. NOX4/NADPH oxidase expression is increased in pulmonary fibroblasts from patients with idiopathic pulmonary fibrosis and mediates TGFβ1-induced fibroblast differentiation into myofibroblasts. Thorax. 2010;65:733–8. https://doi.org/10.1136/thx.2009.113456

17. Ghatak S, Hascall VC, Markwald RR, Feghali-Bostwick C, Artlett CM, Gooz M, et al. Transforming growth factor 1 (TGF1)-induced CD44V6-NOX4 signaling in pathogenesis of idiopathic pulmonary fibrosis. J Biol Chem. 2017;292:10490–519. https://doi.org/10.1074/jbc.M116.752469

18. Ghatak S, Markwald RR, Hascall VC, Dowling W, Lottes RG, Baatz JE, et al. Transforming growth factor β1 (TGF β1) regulates CD44V6 expression and activity through extracellular signal-regulated kinase (ERK)-induced EGR1 in pulmonary fibrogenic fibroblasts. J Biol Chem. 2017;292:10465–89. https://doi.org/10.1074/jbc.M116.752451

19. Effendi WI, Nagano T. Connective Tissue Growth Factor in Idiopathic Pulmonary Fibrosis: Breaking the Bridge. Int J Mol Sci. 2022;23. https://doi.org/10.3390/ijms23116064

20. Wang J, Hu K, Cai X, Yang B, He Q, Wang J, et al. Targeting PI3K/AKT signaling for treatment of idiopathic pulmonary fibrosis. Acta Pharm. Sin. B. Chinese Academy of Medical Sciences; 2022. p. 18–32. https://doi.org/10.1016/j.apsb.2021.07.023

21. Shi L, Dong N, Fang X, Wang X. Regulatory mechanisms of TGF-β1-induced

fibrogenesis of human alveolar epithelial cells. J Cell Mol Med. 2016;20:2183–93. https://doi.org/10.1111/jcmm.12918

22. Qiu Y, Que Y, Ding Z, Zhang S, Wei R, Xia J, et al. Drugs targeting CTGF in the treatment of pulmonary fibrosis. J Cell Mol Med. 2024;28:1–8. https://doi.org/10.1111/jcmm.18448

23. Lin CH, Yu MC, Tung WH, Chen TT, Yu CC, Weng CM, et al. Connective tissue growth factor induces collagen I expression in human lung fibroblasts through the Rac1/MLK3/JNK/AP-1 pathway. Biochim Biophys Acta - Mol Cell Res [Internet]. Elsevier B.V.; 2013;1833:2823–33. https://doi.org/10.1016/j.bbamcr.2013.07.016

24. Shafieian M, Chen S, Wu S. Integrin-linked kinase mediates CTGF-induced epithelial to mesenchymal transition in alveolar type II epithelial cells. Pediatr Res. 2015;77:520–7. https://doi.org/10.1038/pr.2015.8

25. Huang LS, Sudhadevi T, Fu P, Punathil-Kannan PK, Ebenezer DL, Ramchandran R, et al. Sphingosine kinase 1/s1p signaling contributes to pulmonary fibrosis by activating hippo/yap pathway and mitochondrial reactive oxygen species in lung fibroblasts. Int J Mol Sci. 2020;21. https://doi.org/10.3390/ijms21062064

26. Nakamura Y, Shimizu Y. Cellular and Molecular Control of Lipid Metabolism in Idiopathic Pulmonary Fibrosis: Clinical Application of the Lysophosphatidic Acid Pathway. Cells. 2023;12. https://doi.org/10.3390/cells12040548

27. Huang LS, Sudhadevi T, Fu P, Punathil-Kannan PK, Ebenezer DL, Ramchandran R, et al. Sphingosine kinase 1/s1p signaling contributes to pulmonary fibrosis by activating hippo/yap pathway and mitochondrial reactive oxygen species in lung fibroblasts. Int J Mol Sci. MDPI AG; 2020;21. https://doi.org/10.3390/ijms21062064

28. Song JS, Kang CM, Park CK, Yoon HK. Thrombin induces epithelial-mesenchymal transition via PAR-1, PKC, and ERK1/2 pathways in A549 cells. Exp Lung Res.

2013;39:336–48. https://doi.org/10.3109/01902148.2013.820809

29. Huang J, Tong X, Zhang L, Zhang Y, Wang L, Wang D, et al. Hyperoside Attenuates Bleomycin-Induced Pulmonary Fibrosis Development in Mice. Front Pharmacol. Frontiers Media S.A.; 2020;11. https://doi.org/10.3389/fphar.2020.550955

30. Liu L, Sun Q, Davis F, Mao J, Zhao H, Ma D. Epithelial-mesenchymal transition in organ fibrosis development: Current understanding and treatment strategies. Burn. Trauma. Oxford University Press; 2022. https://doi.org/10.1093/burnst/tkac011

31. Polimeni M, Gulino GR, Gazzano E, Kopecka J, Marucco A, Fenoglio I, et al. Multi-walled carbon nanotubes directly induce epithelial-mesenchymal transition in human bronchial epithelial cells via the TGF-β-mediated Akt/GSK-3β/SNAIL-1 signalling pathway. Part Fibre Toxicol. BioMed Central Ltd.; 2016;13. https://doi.org/10.1186/s12989-016-0138-4

32. Lin CH, Shih CH, Tseng CC, Yu CC, Tsai YJ, Bien MY, et al. CXCL12 induces connective tissue growth factor expression in human lung fibroblasts through the Rac1/ERK, JNK, and AP-1 pathways. PLoS One. 2014;9:1–16. https://doi.org/10.1371/journal.pone.0104746

33. Luo L, Zhang W, You S, Cui X, Tu H, Yi Q, et al. The role of epithelial cells in fibrosis: Mechanisms and treatment. Pharmacol Res. 2024;202. https://doi.org/10.1016/j.phrs.2024.107144

34. Peng L, Wen L, Shi QF, Gao F, Huang B, Meng J, et al. Scutellarin ameliorates pulmonary fibrosis through inhibiting NF-κB/NLRP3-mediated epithelial–mesenchymal transition and inflammation. Cell Death Dis [Internet]. Springer US; 2020;11. https://doi.org/10.1038/s41419-020-03178-2

35. Jäger B, Seeliger B, Terwolbeck O, Warnecke G, Welte T, Müller M, et al. The NLRP3-Inflammasome-Caspase-1 Pathway Is Upregulated in Idiopathic Pulmonary Fibrosis and

Acute Exacerbations and Is Inducible by Apoptotic A549 Cells. Front Immunol. 2021;12:1–11. https://doi.org/10.3389/fimmu.2021.642855

36. Li X, Yan X, Wang Y, Wang J, Zhou F, Wang H, et al. NLRP3 inflammasome inhibition attenuates silica-induced epithelial to mesenchymal transition (EMT) in human bronchial epithelial cells. Exp Cell Res [Internet]. Elsevier Inc.; 2018;362:489–97. https://doi.org/10.1016/j.yexcr.2017.12.013

37. Ji J, Hou J, Xia Y, Xiang Z, Han X. NLRP3 inflammasome activation in alveolar epithelial cells promotes myofibroblast differentiation of lung-resident mesenchymal stem cells during pulmonary fibrogenesis. Biochim Biophys Acta - Mol Basis Dis [Internet]. Elsevier B.V.; 2021;1867:166077. https://doi.org/10.1016/j.bbadis.2021.166077

38. Baarsma HA, Königshoff M. "WNT-er is coming": WNT signalling in chronic lung diseases. Thorax. 2017;72:746–59. https://doi.org/10.1136/thoraxjnl-2016-209753

39. Guo Y, Xiao L, Sun L, Liu F. Wnt/β-catenin signaling: A promising new target for fibrosis diseases. Physiol. Res. 2012. p. 337–46. https://doi.org/10.33549/physiolres.932289

40. Huang LS, Fu P, Patel P, Harijith A, Sun T, Zhao Y, et al. Lysophosphatidic acid receptor-2 deficiency confers protection against bleomycin-induced lung injury and fibrosis in mice. Am J Respir Cell Mol Biol. 2013;49:912–22. https://doi.org/10.1165/rcmb.2013-0070OC

41. Sakai N, Tager AM. Fibrosis of two: Epithelial cell-fibroblast interactions in pulmonary fibrosis. Biochim Biophys Acta - Mol Basis Dis [Internet]. Elsevier B.V.; 2013;1832:911–21. https://doi.org/10.1016/j.bbadis.2013.03.001

42. Cao H, Chen X, Hou J, Wang C, Xiang Z, Shen Y, et al. The Shh/Gli signaling cascade regulates myofibroblastic activation of lung-resident mesenchymal stem cells via the modulation of Wnt10a expression during pulmonary fibrogenesis. Lab Investig [Internet]. United States & Canadian Academy of Pathology; 2020;100:363–77.

https://doi.org/10.1038/s41374-019-0316-8

43. Bolaños AL, Milla CM, Lira JC, Ramírez R, Checa M, Barrera L, et al. Role of Sonic Hedgehog in idiopathic pulmonary fibrosis. Am J Physiol - Lung Cell Mol Physiol. 2012;303:978–90. https://doi.org/10.1152/ajplung.00184.2012

44. Kizawa R, Araya J, Fujita Y. Divergent roles of the Hippo pathway in the pathogenesis of idiopathic pulmonary fibrosis: tissue homeostasis and fibrosis. Inflamm. Regen. BioMed Central Ltd; 2023. https://doi.org/10.1186/s41232-023-00295-1

45. Noguchi S, Saito A, Mikami Y, Urushiyama H, Horie M, Matsuzaki H, et al. TAZ contributes to pulmonary fibrosis by activating profibrotic functions of lung fibroblasts. Sci Rep. Nature Publishing Group; 2017;7. https://doi.org/10.1038/srep42595

46. Chen Y, Zhao X, Sun J, Su W, Zhang L, Li Y, et al. YAP1/Twist promotes fibroblast activation and lung fibrosis that conferred by miR-15a loss in IPF. Cell Death Differ. Nature Publishing Group; 2019;26:1832–44. https://doi.org/10.1038/s41418-018-0250-0

47. Liu F, Lagares D, Choi KM, Stopfer L, Marinković A, Vrbanac V, et al. Mechanosignaling through YAP and TAZ drives fibroblast activation and fibrosis. Am J Physiol - Lung Cell Mol Physiol. American Physiological Society; 2015;308:L344–57. https://doi.org/10.1152/ajplung.00300.2014

48. Sava P, Ramanathan A, Dobronyi A, Peng X, Sun H, Ledesma-Mendoza A, et al. Human pericytes adopt myofibroblast properties in the microenvironment of the IPF lung. JCI Insight. 2017;2:1–13. https://doi.org/10.1172/jci.insight.96352

49. Selvarajah B, Azuelos I, Platé M, Guillotin D, Forty EJ, Contento G, et al. MTORC1 amplifies the ATF4-dependent de novo serine-glycine pathway to supply glycine during TGF-1-induced collagen biosynthesis. Sci Signal. 2019;12. https://doi.org/10.1126/scisignal.aav3048

50. Selvarajah B, Azuelos I, Anastasiou D, Chambers RC. Fibrometabolism—An emerging

therapeutic frontier in pulmonary fibrosis. Sci Signal. 2021;14. https://doi.org/10.1126/scisignal.aay1027

51. Milara J, Roger I, Montero P, Artigues E, Escrivá J, Cortijo J. IL-11 system participates in pulmonary artery remodeling and hypertension in pulmonary fibrosis. Respir Res [Internet]. BioMed Central; 2022;23:1–18. https://doi.org/10.1186/s12931-022-02241-0

52. Liu RY, Zeng Y, Lei Z, Wang L, Yang H, Liu Z, et al. JAK/STAT3 signaling is required for TGF-β-induced epithelial- mesenchymal transition in lung cancer cells. Int J Oncol. 2014;44:1643–51. https://doi.org/10.3892/ijo.2014.2310

53. Milara J, Hernandez G, Ballester B, Morell A, Roger I, Montero P, et al. The JAK2 pathway is activated in idiopathic pulmonary fibrosis. Respir Res. Respiratory Research; 2018;19:1–12. https://doi.org/10.1186/s12931-018-0728-9

54. Jarman ER, Khambata VS, Cope C, Jones P, Roger J, Ye LY, et al. An inhibitor of NADPH oxidase-4 attenuates established pulmonary fibrosis in a rodent disease model. 2013;1–30. https://doi.org/10.1165/rcmb.2013-0174OC

55. Noguchi S, Saito A, Mikami Y, Urushiyama H, Horie M, Matsuzaki H, et al. TAZ contributes to pulmonary fibrosis by activating profibrotic functions of lung fibroblasts. Sci Rep. Nature Publishing Group; 2017;7:1–11. https://doi.org/10.1038/srep42595

56. Chen Y, Zhao X, Sun J, Su W, Zhang L, Li Y, et al. YAP1/Twist promotes fibroblast activation and lung fibrosis that conferred by miR-15a loss in IPF. Cell Death Differ. 2019;26:1832–44. https://doi.org/10.1038/s41418-018-0250-0

57. Kim MS, Baek AR, Lee JH, Jang AS, Kim DJ, Chin SS, et al. IL-37 Attenuates Lung Fibrosis by Inducing Autophagy and Regulating TGF-β1 Production in Mice. J Immunol. 2019;203:2265–75. https://doi.org/10.4049/jimmunol.1801515

58. Zhao W, Wang L, Yang J, Chen X, Guo X, Xu K, et al. Endothelial cell-derived MMP19 promotes pulmonary fibrosis by inducing E(nd)MT and monocyte infiltration. Cell Commun

Signal [Internet]. BioMed Central; 2023;21:1–17. https://doi.org/10.1186/s12964-023-01040-4
59. Yasutomo K. Genetics and animal models of familial pulmonary fibrosis. Int Immunol. 2021;33:653–7. https://doi.org/10.1093/intimm/dxab026
60. Mulugeta S, Nureki SI, Beers MF. Lost after translation: Insights from pulmonary surfactant for understanding the role of alveolar epithelial dysfunction and cellular quality control in fibrotic lung disease. Am J Physiol - Lung Cell Mol Physiol. American Physiological Society; 2015;309:L507–25. https://doi.org/10.1152/ajplung.00139.2015
61. Lawson WE, Grant SW, Ambrosini V, Womble KE, Dawson EP, Lane KB, et al. Genetic mutations in surfactant protein C are a rare cause of sporadic cases of IPF. Thorax. 2004;59:977–80. https://doi.org/10.1136/thx.2004.026336
62. Venosa A, Katzen J, Tomer Y, Kopp M, Jamil S, Russo SJ, et al. Epithelial Expression of an Interstitial Lung Disease–Associated Mutation in Surfactant Protein-C Modulates Recruitment and Activation of Key Myeloid Cell Populations in Mice. J Immunol. The American Association of Immunologists; 2019;202:2760–71. https://doi.org/10.4049/jimmunol.1900039
63. Venosa A, Cowman S, Katzen J, Tomer Y, Armstrong BS, Mulugeta S, et al. Role of CCR2+ Myeloid Cells in Inflammation Responses Driven by Expression of a Surfactant Protein-C Mutant in the Alveolar Epithelium. Front Immunol. Frontiers Media S.A.; 2021;12. https://doi.org/10.3389/fimmu.2021.665818
64. Winters NI, Burman A, Kropski JA, Blackwell TS. Epithelial Injury and Dysfunction in the Pathogenesis of Idiopathic PulmonaryFibrosis. Am J Med Sci. 2019;357:374–8. https://doi.org/10.1016/j.amjms.2019.01.010
65. Hancock LA, Hennessy CE, Solomon GM, Dobrinskikh E, Estrella A, Hara N, et al. Muc5b overexpression causes mucociliary dysfunction and enhances lung fibrosis in mice.

Nat Commun [Internet]. Springer US; 2018;9:1–10. https://doi.org/10.1038/s41467-018-07768-9

66. Mustafin RN. Molecular genetics of idiopathic pulmonary fibrosis. Vavilovskii Zhurnal Genet Selektsii. 2022;26:308–18. https://doi.org/10.18699/VJGB-22-37

67. Fernandez BA, Fox G, Bhatia R, Sala E, Noble B, Denic N, et al. A Newfoundland cohort of familial and sporadic idiopathic pulmonary fibrosis patients: clinical and genetic features. Respir Res. 2012;13:1–10. https://doi.org/10.1186/1465-9921-13-64

68. Allen RJ, Guillen-Guio B, Oldham JM, Ma SF, Dressen A, Paynton ML, et al. Genome-wide association study of susceptibility to idiopathic pulmonary fibrosis. Am J Respir Crit Care Med. 2020;201:564–74. https://doi.org/10.1164/rccm.201905-1017OC

69. Stuart BD, Choi J, Zaidi S, Xing C, Holohan B, Chen R, et al. Exome sequencing links mutations in PARN and RTEL1 with familial pulmonary fibrosis and telomere shortening. Nat Genet [Internet]. 2015;47:512–7. https://doi.org/10.1038/ng.3278

70. Tsakiri KD, Cronkhite JT, Kuan PJ, Xing C, Raghu G, Weissler JC, et al. Adult-onset pulmonary fibrosis caused by mutations in telomerase. Proc Natl Acad Sci U S A. 2007;104:7552–7. https://doi.org/10.1073/pnas.0701009104

71. Noth I, Zhang Y, Ma SF, Flores, C., Barber M, Huang Y, Garcia JG. Genetic variants associated with idiopathic pulmonary fibrosis susceptibility and mortality: a genome-wide association study. Int Conf Biomed Robot Biomechatron. 2013;23:244–51. https://doi.org/10.1016/S2213-2600(13)70045-6

72. Chakraborty A, Mastalerz M, Ansari M, Schiller HB, Staab-Weijnitz CA. Emerging Roles of Airway Epithelial Cells in Idiopathic Pulmonary Fibrosis. Cells. 2022;11. https://doi.org/10.3390/cells11061050

73. D’Agnano V, Mariniello DF, Ruotolo M, Quarcio G, Moriello A, Conte S, et al. Targeting Progression in Pulmonary Fibrosis: An Overview of Underlying Mechanisms,

Molecular Biomarkers, and Therapeutic Intervention. Life. 2024;14. https://doi.org/10.3390/life14020229

74. Glass DS, Grossfeld D, Renna HA, Agarwala P, Spiegler P, DeLeon J, et al. Idiopathic pulmonary fibrosis: Current and future treatment. Clin Respir J. 2022;16:84–96. https://doi.org/10.1111/crj.13466

75. Jia W, Wang Z, Gao C, Wu J, Wu Q. Trajectory modeling of endothelial-to-mesenchymal transition reveals galectin-3 as a mediator in pulmonary fibrosis. Cell Death Dis [Internet]. Springer US; 2021;12. https://doi.org/10.1038/s41419-021-03603-0

76. Ayyadurai VAS, Dewey CF. CytoSolve: A scalable computational method for dynamic integration of multiple molecular pathway models. Cell Mol Bioeng. 2011;4:28–45. https://doi.org/10.1007/s12195-010-0143-x

77. Nordsletten DA, Yankama B, Umeton R, Ayyadurai VAS, Dewey CF. Multiscale mathematical modeling to support drug development. IEEE Trans Biomed Eng. 2011;58:3508–12. https://doi.org/10.1109/TBME.2011.2173245

78. Al-Lazikani B, Banerji U, Workman P. Combinatorial drug therapy for cancer in the post-genomic era. Nat Biotechnol [Internet]. Nature Publishing Group; 2012;30:679–92. https://doi.org/10.1038/nbt.2284

79. Sweeney MD, Ayyadurai S, Zlokovic B V. Pericytes of the neurovascular unit: Key functions and signaling pathways. Nat. Neurosci. Nature Publishing Group; 2016. p. 771–83. https://doi.org/10.1038/nn.4288

80. Koo A, Nordsletten D, Umeton R, Yankama B, Ayyadurai S, García-Cardeña G, et al. In silico modeling of shear-stress-induced nitric oxide production in endothelial cells through systems biology. Biophys J. Biophysical Society; 2013;104:2295–306. https://doi.org/10.1016/j.bpj.2013.03.052

81. Food and Drug Administration Center for Drug Evaluation and Research. Request for

Determination of Exempt Status of Investigational New Drug Application (IND) for Cyto-001 as Treatment for Patients with Pancreatic Cancer (PIND: 118833). 5901-B Ammendale Rd., Beltsville, MD, 2013.; 2013.

82. Ayyadurai VAS, Deonikar P, McLure KG, Sakamoto KM. Molecular Systems Architecture of Interactome in the Acute Myeloid Leukemia Microenvironment. Cancers (Basel). MDPI; 2022. https://doi.org/10.3390/cancers14030756

83. Ayyadurai VAS, Deonikar P. Do GMOs Accumulate Formaldehyde and Disrupt Molecular Systems Equilibria? Systems Biology May Provide Answers. Agric Sci. 2015;06:630–62. https://doi.org/10.4236/as.2015.67062

84. Kothandaram S, Deonikar P, Mohan M, Venugopal V, Ayyadurai VAS. <i>In Silico</i> Modeling of C1 Metabolism. Am J Plant Sci. 2015;06:1444–65. https://doi.org/10.4236/ajps.2015.69144

85. Mohan M, Kothandaram S, Venugopal V, Deonikar P, Ayyadurai VAS. Integrative Modeling of Oxidative Stress and C1 Metabolism Reveals Upregulation of Formaldehyde and Downregulation of Glutathione. Am J Plant Sci. 2015;06:1527–42. https://doi.org/10.4236/ajps.2015.69152

86. Ayyadurai VAS, Hansen M, Fagan J, Deonikar P. In-Silico Analysis & In-Vivo Results Concur on Glutathione Depletion in Glyphosate Resistant GMO Soy, Advancing a Systems Biology Framework for Safety Assessment of GMOs. Am J Plant Sci. 2016;07:1571–89. https://doi.org/10.4236/ajps.2016.712149

87. Ayyadurai VAS, Deonikar P. Bioactive compounds in green tea may improve transplant tolerance: A computational systems biology analysis. Clin Nutr ESPEN. Elsevier Ltd; 2021;46:439–52. https://doi.org/10.1016/j.clnesp.2021.09.012

88. Ayyadurai VAS, Deonikar P, Bannuru RR. Attenuation of low-grade chronic inflammation by phytonutrients: A computational systems biology analysis. Clin Nutr

ESPEN [Internet]. 2022;49:425–35. https://doi.org/10.1016/j.clnesp.2022.03.010
89. Cornish-Bowden A. One hundred years of Michaelis–Menten kinetics. Perspect Sci [Internet]. Elsevier; 2015;4:3–9. https://doi.org/10.1016/j.pisc.2014.12.002
90. Michaelis L, Menten ML, Goody RS, Johnson KA. Die Kinetik der Invertinwirkung/ The kinetics of invertase action. Biochemistry. 1913;
91. Hucka M, Finney A, Sauro HM, Bolouri H, Doyle JC, Kitano H, et al. The systems biology markup language (SBML): A medium for representation and exchange of biochemical network models. Bioinformatics. 2003;19:524–31. https://doi.org/10.1093/bioinformatics/btg015
92. Ayyadurai VAS. Services-Based Systems Architecture for Modeling the Whole Cell: A Distributed Collaborative Engineering Systems Approach. 2010;115–68. https://doi.org/10.1007/8754_2010_1
93. Shmulevich I, Aitchison JD. Deterministic and Stochastic Models of Genetic Regulatory Networks. Methods Enzymol. 2009;467:335–56. https://doi.org/10.1016/S0076-6879(09)67013-0
94. Oden JT (John T, Reddy JN (Junuthula N. An Introduction to the Mathematical Theory of Finite Elements. 1976th ed. New York: Dover Publications; 2011.
95. Cumming G, Fidler F, Vaux DL. Error bars in experimental biology. J Cell Biol. 2007;177:7–11. https://doi.org/10.1083/jcb.200611141
96. Thanh VH, Zunino R, Priami C. Efficient finite-difference method for computing sensitivities of biochemical reactions. Proc R Soc A Math Phys Eng Sci. The Royal Society Publishing; 2018;474. https://doi.org/10.1098/RSPA.2018.0303
1. Rajesh R, Atallah R, Bärnthaler T. Dysregulation of metabolic pathways in pulmonary fibrosis. Pharmacol Ther. 2023;246. https://doi.org/10.1016/j.pharmthera.2023.108436
2. Roque W, Romero F. Cellular metabolomics of pulmonary fibrosis, from amino acids to

lipids. Am J Physiol - Cell Physiol. 2021;320:C689–95. https://doi.org/10.1152/ajpcell.00586.2020

3. Mora AL, Torres-González E, Rojas M, Corredor C, Ritzenthaler J, Xu J, et al. Activation of alveolar macrophages via the alternative pathway in herpesvirus-induced lung fibrosis. Am J Respir Cell Mol Biol. 2006;35:466–73. https://doi.org/10.1165/rcmb.2006-0121OC

4. Liu S, Liu C, Wang Q, Liu S, Min J. CC Chemokines in Idiopathic Pulmonary Fibrosis: Pathogenic Role and Therapeutic Potential. Biomolecules. 2023;13:1–20. https://doi.org/10.3390/biom13020333

5. Luzina IG, Tsymbalyuk N, Choi J, Hasday JD, Atamas SP. CCL18-stimulated upregulation of collagen production in lung fibroblasts requires Sp1 signaling and basal Smad3 activity. J Cell Physiol. 2006;206:221–8. https://doi.org/10.1002/jcp.20452

6. Zhang L, Wang Y, Wu G, Xiong W, Gu W, Wang CY. Macrophages: Friend or foe in idiopathic pulmonary fibrosis? Respir Res. Respiratory Research; 2018;19:1–10. https://doi.org/10.1186/s12931-018-0864-2

7. Li D, Guabiraba R, Besnard AG, Komai-Koma M, Jabir MS, Zhang L, et al. IL-33 promotes ST2-dependent lung fibrosis by the induction of alternatively activated macrophages and innate lymphoid cells in mice. J Allergy Clin Immunol. 2014;134:1422-1432.e11. https://doi.org/10.1016/j.jaci.2014.05.011

8. She YX, Yu QY, Tang XX. Role of interleukins in the pathogenesis of pulmonary fibrosis. Cell Death Discov. 2021;7. https://doi.org/10.1038/s41420-021-00437-9

9. Zhao J, Okamoto Y, Asano Y, Ishimaru K, Aki S, Yoshioka K, et al. Sphingosine-1-phosphate receptor-2 facilitates pulmonary fibrosis through potentiating IL-13 pathway in macrophages. PLoS One. 2018;13:1–22. https://doi.org/10.1371/journal.pone.0197604

10. Celada LJ, Kropski JA, Herazo-Maya JD, Luo W, Creecy A, Abad AT, et al. PD-1 up-regulation on CD4+ T cells promotes pulmonary fibrosis through STAT3-mediated IL-17A and TGF-β1 production. Sci Transl Med. 2018;10. https://doi.org/10.1126/scitranslmed.aar8356

11. Guo X, Sunil C, Adeyanju O, Parker A, Huang S, Ikebe M, et al. PD-L1 mediates lung fibroblast to myofibroblast transition through Smad3 and β-catenin signaling pathways. Sci Rep [Internet]. Nature Publishing Group UK; 2022;12:1–14. https://doi.org/10.1038/s41598-022-07044-3

12. Wang D, Gong L, Li Z, Chen H, Xu M, Rong R, et al. Antifibrotic effect of Gancao Ganjiang decoction is mediated by PD-1 / TGF-β1 / IL-17A pathway in bleomycin-induced idiopathic pulmonary fibrosis. J Ethnopharmacol [Internet]. Elsevier B.V.; 2021;281:114522. https://doi.org/10.1016/j.jep.2021.114522

13. Jarman ER, Khambata VS, Cope C, Jones P, Roger J, Ye LY, et al. An Inhibitor of NADPH oxidase-4 attenuates established pulmonary fibrosis in a rodent disease model. Am J Respir Cell Mol Biol. 2014;50:158–69. https://doi.org/10.1165/rcmb.2013-0174OC

14. Ismail S, Sturrock A, Wu P, Cahill B, Norman K, Huecksteadt T, et al. NOX4 mediates hypoxia-induced proliferation of human pulmonary artery smooth muscle cells: The role of autocrine production of transforming growth factor-β1 and insulin-like growth factor binding protein-3. Am J Physiol - Lung Cell Mol Physiol. 2009;296. https://doi.org/10.1152/ajplung.90488.2008

15. Liu RM, Choi J, Wu JH, Pravia KAG, Lewis KM, Brand JD, et al. Oxidative modification of nuclear mitogen-activated protein kinase phosphatase 1 is involved in transforming growth factor β1-induced expression of plasminogen activator inhibitor 1 in fibroblasts. J Biol Chem. 2010;285:16239–47. https://doi.org/10.1074/jbc.M110.111732

16. Amara N, Goven D, Prost F, Muloway R, Crestani B, Boczkowski J. NOX4/NADPH oxidase expression is increased in pulmonary fibroblasts from patients with idiopathic pulmonary fibrosis and mediates TGFβ1-induced fibroblast differentiation into myofibroblasts. Thorax. 2010;65:733–8. https://doi.org/10.1136/thx.2009.113456

17. Ghatak S, Hascall VC, Markwald RR, Feghali-Bostwick C, Artlett CM, Gooz M, et al. Transforming growth factor 1 (TGF1)-induced CD44V6-NOX4 signaling in pathogenesis of idiopathic pulmonary fibrosis. J Biol Chem. 2017;292:10490–519. https://doi.org/10.1074/jbc.M116.752469

18. Ghatak S, Markwald RR, Hascall VC, Dowling W, Lottes RG, Baatz JE, et al. Transforming growth factor β1 (TGF β1) regulates CD44V6 expression and activity through extracellular signal-regulated kinase (ERK)-induced EGR1 in pulmonary fibrogenic fibroblasts. J Biol Chem. 2017;292:10465–89. https://doi.org/10.1074/jbc.M116.752451

19. Effendi WI, Nagano T. Connective Tissue Growth Factor in Idiopathic Pulmonary Fibrosis: Breaking the Bridge. Int J Mol Sci. 2022;23. https://doi.org/10.3390/ijms23116064

20. Wang J, Hu K, Cai X, Yang B, He Q, Wang J, et al. Targeting PI3K/AKT signaling for treatment of idiopathic pulmonary fibrosis. Acta Pharm. Sin. B. Chinese Academy of Medical Sciences; 2022. p. 18–32. https://doi.org/10.1016/j.apsb.2021.07.023

21. Shi L, Dong N, Fang X, Wang X. Regulatory mechanisms of TGF-β1-induced fibrogenesis of human alveolar epithelial cells. J Cell Mol Med. 2016;20:2183–93. https://doi.org/10.1111/jcmm.12918

22. Qiu Y, Que Y, Ding Z, Zhang S, Wei R, Xia J, et al. Drugs targeting CTGF in the treatment of pulmonary fibrosis. J Cell Mol Med. 2024;28:1–8. https://doi.org/10.1111/jcmm.18448

23. Lin CH, Yu MC, Tung WH, Chen TT, Yu CC, Weng CM, et al. Connective tissue growth factor induces collagen I expression in human lung fibroblasts through the Rac1/MLK3/JNK/AP-1 pathway. Biochim Biophys Acta - Mol Cell Res [Internet]. Elsevier B.V.; 2013;1833:2823–33. https://doi.org/10.1016/j.bbamcr.2013.07.016

24. Shafieian M, Chen S, Wu S. Integrin-linked kinase mediates CTGF-induced epithelial to mesenchymal transition in alveolar type II epithelial cells. Pediatr Res. 2015;77:520–7. https://doi.org/10.1038/pr.2015.8

25. Huang LS, Sudhadevi T, Fu P, Punathil-Kannan PK, Ebenezer DL, Ramchandran R, et al. Sphingosine kinase 1/s1p signaling contributes to pulmonary fibrosis by activating hippo/yap pathway and mitochondrial reactive oxygen species in lung fibroblasts. Int J Mol Sci. 2020;21. https://doi.org/10.3390/ijms21062064

26. Nakamura Y, Shimizu Y. Cellular and Molecular Control of Lipid Metabolism in Idiopathic Pulmonary Fibrosis: Clinical Application of the Lysophosphatidic Acid Pathway. Cells. 2023;12. https://doi.org/10.3390/cells12040548

27. Huang LS, Sudhadevi T, Fu P, Punathil-Kannan PK, Ebenezer DL, Ramchandran R, et al. Sphingosine kinase 1/s1p signaling contributes to pulmonary fibrosis by activating hippo/yap pathway and mitochondrial reactive oxygen species in lung fibroblasts. Int J Mol Sci. MDPI AG; 2020;21. https://doi.org/10.3390/ijms21062064

28. Song JS, Kang CM, Park CK, Yoon HK. Thrombin induces epithelial-mesenchymal transition via PAR-1, PKC, and ERK1/2 pathways in A549 cells. Exp Lung Res. 2013;39:336–48. https://doi.org/10.3109/01902148.2013.820809

29. Huang J, Tong X, Zhang L, Zhang Y, Wang L, Wang D, et al. Hyperoside Attenuates Bleomycin-Induced Pulmonary Fibrosis Development in Mice. Front Pharmacol. Frontiers Media S.A.; 2020;11. https://doi.org/10.3389/fphar.2020.550955

30. Liu L, Sun Q, Davis F, Mao J, Zhao H, Ma D. Epithelial-mesenchymal transition in organ fibrosis development: Current understanding and treatment strategies. Burn. Trauma. Oxford University Press; 2022. https://doi.org/10.1093/burnst/tkac011

31. Polimeni M, Gulino GR, Gazzano E, Kopecka J, Marucco A, Fenoglio I, et al. Multi-walled carbon nanotubes directly induce epithelial-mesenchymal transition in human bronchial epithelial cells via the TGF-β-mediated Akt/GSK-3β/SNAIL-1 signalling pathway. Part Fibre Toxicol. BioMed Central Ltd.; 2016;13. https://doi.org/10.1186/s12989-016-0138-4

32. Lin CH, Shih CH, Tseng CC, Yu CC, Tsai YJ, Bien MY, et al. CXCL12 induces connective tissue growth factor expression in human lung fibroblasts through the Rac1/ERK, JNK, and AP-1 pathways. PLoS One. 2014;9:1–16. https://doi.org/10.1371/journal.pone.0104746

33. Luo L, Zhang W, You S, Cui X, Tu H, Yi Q, et al. The role of epithelial cells in fibrosis: Mechanisms and treatment. Pharmacol Res. 2024;202. https://doi.org/10.1016/j.phrs.2024.107144

34. Peng L, Wen L, Shi QF, Gao F, Huang B, Meng J, et al. Scutellarin ameliorates pulmonary fibrosis through inhibiting NF-κB/NLRP3-mediated epithelial–mesenchymal transition and inflammation. Cell Death Dis [Internet]. Springer US; 2020;11. https://doi.org/10.1038/s41419-020-03178-2

35. Jäger B, Seeliger B, Terwolbeck O, Warnecke G, Welte T, Müller M, et al. The NLRP3-Inflammasome-Caspase-1 Pathway Is Upregulated in Idiopathic Pulmonary Fibrosis and Acute Exacerbations and Is Inducible by Apoptotic A549 Cells. Front Immunol. 2021;12:1–11. https://doi.org/10.3389/fimmu.2021.642855

36. Li X, Yan X, Wang Y, Wang J, Zhou F, Wang H, et al. NLRP3 inflammasome inhibition

attenuates silica-induced epithelial to mesenchymal transition (EMT) in human bronchial epithelial cells. Exp Cell Res [Internet]. Elsevier Inc.; 2018;362:489–97. https://doi.org/10.1016/j.yexcr.2017.12.013

37. Ji J, Hou J, Xia Y, Xiang Z, Han X. NLRP3 inflammasome activation in alveolar epithelial cells promotes myofibroblast differentiation of lung-resident mesenchymal stem cells during pulmonary fibrogenesis. Biochim Biophys Acta - Mol Basis Dis [Internet]. Elsevier B.V.; 2021;1867:166077. https://doi.org/10.1016/j.bbadis.2021.166077

38. Baarsma HA, Königshoff M. "WNT-er is coming": WNT signalling in chronic lung diseases. Thorax. 2017;72:746–59. https://doi.org/10.1136/thoraxjnl-2016-209753

39. Guo Y, Xiao L, Sun L, Liu F. Wnt/β-catenin signaling: A promising new target for fibrosis diseases. Physiol. Res. 2012. p. 337–46. https://doi.org/10.33549/physiolres.932289

40. Huang LS, Fu P, Patel P, Harijith A, Sun T, Zhao Y, et al. Lysophosphatidic acid receptor-2 deficiency confers protection against bleomycin-induced lung injury and fibrosis in mice. Am J Respir Cell Mol Biol. 2013;49:912–22. https://doi.org/10.1165/rcmb.2013-0070OC

41. Sakai N, Tager AM. Fibrosis of two: Epithelial cell-fibroblast interactions in pulmonary fibrosis. Biochim Biophys Acta - Mol Basis Dis [Internet]. Elsevier B.V.; 2013;1832:911–21. https://doi.org/10.1016/j.bbadis.2013.03.001

42. Cao H, Chen X, Hou J, Wang C, Xiang Z, Shen Y, et al. The Shh/Gli signaling cascade regulates myofibroblastic activation of lung-resident mesenchymal stem cells via the modulation of Wnt10a expression during pulmonary fibrogenesis. Lab Investig [Internet]. United States & Canadian Academy of Pathology; 2020;100:363–77. https://doi.org/10.1038/s41374-019-0316-8

43. Bolaños AL, Milla CM, Lira JC, Ramírez R, Checa M, Barrera L, et al. Role of Sonic Hedgehog in idiopathic pulmonary fibrosis. Am J Physiol - Lung Cell Mol Physiol. 2012;303:978–90. https://doi.org/10.1152/ajplung.00184.2012

44. Kizawa R, Araya J, Fujita Y. Divergent roles of the Hippo pathway in the pathogenesis of idiopathic pulmonary fibrosis: tissue homeostasis and fibrosis. Inflamm. Regen. BioMed Central Ltd; 2023. https://doi.org/10.1186/s41232-023-00295-1

45. Noguchi S, Saito A, Mikami Y, Urushiyama H, Horie M, Matsuzaki H, et al. TAZ contributes to pulmonary fibrosis by activating profibrotic functions of lung fibroblasts. Sci Rep. Nature Publishing Group; 2017;7. https://doi.org/10.1038/srep42595

46. Chen Y, Zhao X, Sun J, Su W, Zhang L, Li Y, et al. YAP1/Twist promotes fibroblast activation and lung fibrosis that conferred by miR-15a loss in IPF. Cell Death Differ. Nature Publishing Group; 2019;26:1832–44. https://doi.org/10.1038/s41418-018-0250-0

47. Liu F, Lagares D, Choi KM, Stopfer L, Marinković A, Vrbanac V, et al. Mechanosignaling through YAP and TAZ drives fibroblast activation and fibrosis. Am J Physiol - Lung Cell Mol Physiol. American Physiological Society; 2015;308:L344–57. https://doi.org/10.1152/ajplung.00300.2014

48. Sava P, Ramanathan A, Dobronyi A, Peng X, Sun H, Ledesma-Mendoza A, et al. Human pericytes adopt myofibroblast properties in the microenvironment of the IPF lung. JCI Insight. 2017;2:1–13. https://doi.org/10.1172/jci.insight.96352

49. Selvarajah B, Azuelos I, Platé M, Guillotin D, Forty EJ, Contento G, et al. MTORC1 amplifies the ATF4-dependent de novo serine-glycine pathway to supply glycine during TGF-1-induced collagen biosynthesis. Sci Signal. 2019;12. https://doi.org/10.1126/scisignal.aav3048

50. Selvarajah B, Azuelos I, Anastasiou D, Chambers RC. Fibrometabolism—An emerging therapeutic frontier in pulmonary fibrosis. Sci Signal. 2021;14. https://doi.org/10.1126/scisignal.aay1027

51. Milara J, Roger I, Montero P, Artigues E, Escrivá J, Cortijo J. IL-11 system participates in pulmonary artery remodeling and hypertension in pulmonary fibrosis. Respir Res [Internet]. BioMed Central; 2022;23:1–18. https://doi.org/10.1186/s12931-022-02241-0

52. Liu RY, Zeng Y, Lei Z, Wang L, Yang H, Liu Z, et al. JAK/STAT3 signaling is required for TGF-β-induced epithelial- mesenchymal transition in lung cancer cells. Int J Oncol. 2014;44:1643–51. https://doi.org/10.3892/ijo.2014.2310

53. Milara J, Hernandez G, Ballester B, Morell A, Roger I, Montero P, et al. The JAK2 pathway is activated in idiopathic pulmonary fibrosis. Respir Res. Respiratory Research; 2018;19:1–12. https://doi.org/10.1186/s12931-018-0728-9

54. Jarman ER, Khambata VS, Cope C, Jones P, Roger J, Ye LY, et al. An inhibitor of NADPH oxidase-4 attenuates established pulmonary fibrosis in a rodent disease model. 2013;1–30. https://doi.org/10.1165/rcmb.2013-0174OC

55. Noguchi S, Saito A, Mikami Y, Urushiyama H, Horie M, Matsuzaki H, et al. TAZ contributes to pulmonary fibrosis by activating profibrotic functions of lung fibroblasts. Sci Rep. Nature Publishing Group; 2017;7:1–11. https://doi.org/10.1038/srep42595

56. Chen Y, Zhao X, Sun J, Su W, Zhang L, Li Y, et al. YAP1/Twist promotes fibroblast activation and lung fibrosis that conferred by miR-15a loss in IPF. Cell Death Differ. 2019;26:1832–44. https://doi.org/10.1038/s41418-018-0250-0

57. Kim MS, Baek AR, Lee JH, Jang AS, Kim DJ, Chin SS, et al. IL-37 Attenuates Lung Fibrosis by Inducing Autophagy and Regulating TGF-β1 Production in Mice. J Immunol.

2019;203:2265–75. https://doi.org/10.4049/jimmunol.1801515

58. Zhao W, Wang L, Yang J, Chen X, Guo X, Xu K, et al. Endothelial cell-derived MMP19 promotes pulmonary fibrosis by inducing E(nd)MT and monocyte infiltration. Cell Commun Signal [Internet]. BioMed Central; 2023;21:1–17. https://doi.org/10.1186/s12964-023-01040-4

59. Yasutomo K. Genetics and animal models of familial pulmonary fibrosis. Int Immunol. 2021;33:653–7. https://doi.org/10.1093/intimm/dxab026

60. Mulugeta S, Nureki SI, Beers MF. Lost after translation: Insights from pulmonary surfactant for understanding the role of alveolar epithelial dysfunction and cellular quality control in fibrotic lung disease. Am J Physiol - Lung Cell Mol Physiol. American Physiological Society; 2015;309:L507–25. https://doi.org/10.1152/ajplung.00139.2015

61. Lawson WE, Grant SW, Ambrosini V, Womble KE, Dawson EP, Lane KB, et al. Genetic mutations in surfactant protein C are a rare cause of sporadic cases of IPF. Thorax. 2004;59:977–80. https://doi.org/10.1136/thx.2004.026336

62. Venosa A, Katzen J, Tomer Y, Kopp M, Jamil S, Russo SJ, et al. Epithelial Expression of an Interstitial Lung Disease–Associated Mutation in Surfactant Protein-C Modulates Recruitment and Activation of Key Myeloid Cell Populations in Mice. J Immunol. The American Association of Immunologists; 2019;202:2760–71. https://doi.org/10.4049/jimmunol.1900039

63. Venosa A, Cowman S, Katzen J, Tomer Y, Armstrong BS, Mulugeta S, et al. Role of CCR2+ Myeloid Cells in Inflammation Responses Driven by Expression of a Surfactant Protein-C Mutant in the Alveolar Epithelium. Front Immunol. Frontiers Media S.A.; 2021;12. https://doi.org/10.3389/fimmu.2021.665818

64. Winters NI, Burman A, Kropski JA, Blackwell TS. Epithelial Injury and Dysfunction in the Pathogenesis of Idiopathic PulmonaryFibrosis. Am J Med Sci. 2019;357:374–8. https://doi.org/10.1016/j.amjms.2019.01.010

65. Hancock LA, Hennessy CE, Solomon GM, Dobrinskikh E, Estrella A, Hara N, et al. Muc5b overexpression causes mucociliary dysfunction and enhances lung fibrosis in mice. Nat Commun [Internet]. Springer US; 2018;9:1–10. https://doi.org/10.1038/s41467-018-07768-9

66. Mustafin RN. Molecular genetics of idiopathic pulmonary fibrosis. Vavilovskii Zhurnal Genet Selektsii. 2022;26:308–18. https://doi.org/10.18699/VJGB-22-37

67. Fernandez BA, Fox G, Bhatia R, Sala E, Noble B, Denic N, et al. A Newfoundland cohort of familial and sporadic idiopathic pulmonary fibrosis patients: clinical and genetic features. Respir Res. 2012;13:1–10. https://doi.org/10.1186/1465-9921-13-64

68. Allen RJ, Guillen-Guio B, Oldham JM, Ma SF, Dressen A, Paynton ML, et al. Genome-wide association study of susceptibility to idiopathic pulmonary fibrosis. Am J Respir Crit Care Med. 2020;201:564–74. https://doi.org/10.1164/rccm.201905-1017OC

69. Stuart BD, Choi J, Zaidi S, Xing C, Holohan B, Chen R, et al. Exome sequencing links mutations in PARN and RTEL1 with familial pulmonary fibrosis and telomere shortening. Nat Genet [Internet]. 2015;47:512–7. https://doi.org/10.1038/ng.3278

70. Tsakiri KD, Cronkhite JT, Kuan PJ, Xing C, Raghu G, Weissler JC, et al. Adult-onset pulmonary fibrosis caused by mutations in telomerase. Proc Natl Acad Sci U S A. 2007;104:7552–7. https://doi.org/10.1073/pnas.0701009104

71. Noth I, Zhang Y, Ma SF, Flores, C., Barber M, Huang Y, Garcia JG. Genetic variants associated with idiopathic pulmonary fibrosis susceptibility and mortality: a genome-wide

association study. Int Conf Biomed Robot Biomechatron. 2013;23:244–51. https://doi.org/10.1016/S2213-2600(13)70045-6

72. Chakraborty A, Mastalerz M, Ansari M, Schiller HB, Staab-Weijnitz CA. Emerging Roles of Airway Epithelial Cells in Idiopathic Pulmonary Fibrosis. Cells. 2022;11. https://doi.org/10.3390/cells11061050

73. D'Agnano V, Mariniello DF, Ruotolo M, Quarcio G, Moriello A, Conte S, et al. Targeting Progression in Pulmonary Fibrosis: An Overview of Underlying Mechanisms, Molecular Biomarkers, and Therapeutic Intervention. Life. 2024;14. https://doi.org/10.3390/life14020229

74. Glass DS, Grossfeld D, Renna HA, Agarwala P, Spiegler P, DeLeon J, et al. Idiopathic pulmonary fibrosis: Current and future treatment. Clin Respir J. 2022;16:84–96. https://doi.org/10.1111/crj.13466

75. Jia W, Wang Z, Gao C, Wu J, Wu Q. Trajectory modeling of endothelial-to-mesenchymal transition reveals galectin-3 as a mediator in pulmonary fibrosis. Cell Death Dis [Internet]. Springer US; 2021;12. https://doi.org/10.1038/s41419-021-03603-0

76. Ayyadurai VAS, Dewey CF. CytoSolve: A scalable computational method for dynamic integration of multiple molecular pathway models. Cell Mol Bioeng. 2011;4:28–45. https://doi.org/10.1007/s12195-010-0143-x

77. Nordsletten DA, Yankama B, Umeton R, Ayyadurai VAS, Dewey CF. Multiscale mathematical modeling to support drug development. IEEE Trans Biomed Eng. 2011;58:3508–12. https://doi.org/10.1109/TBME.2011.2173245

78. Al-Lazikani B, Banerji U, Workman P. Combinatorial drug therapy for cancer in the post-genomic era. Nat Biotechnol [Internet]. Nature Publishing Group; 2012;30:679–92.

https://doi.org/10.1038/nbt.2284

79. Sweeney MD, Ayyadurai S, Zlokovic B V. Pericytes of the neurovascular unit: Key functions and signaling pathways. Nat. Neurosci. Nature Publishing Group; 2016. p. 771–83. https://doi.org/10.1038/nn.4288

80. Koo A, Nordsletten D, Umeton R, Yankama B, Ayyadurai S, García-Cardeña G, et al. In silico modeling of shear-stress-induced nitric oxide production in endothelial cells through systems biology. Biophys J. Biophysical Society; 2013;104:2295–306. https://doi.org/10.1016/j.bpj.2013.03.052

81. Food and Drug Administration Center for Drug Evaluation and Research. Request for Determination of Exempt Status of Investigational New Drug Application (IND) for Cyto-001 as Treatment for Patients with Pancreatic Cancer (PIND: 118833). 5901-B Ammendale Rd., Beltsville, MD, 2013.; 2013.

82. Ayyadurai VAS, Deonikar P, McLure KG, Sakamoto KM. Molecular Systems Architecture of Interactome in the Acute Myeloid Leukemia Microenvironment. Cancers (Basel). MDPI; 2022. https://doi.org/10.3390/cancers14030756

83. Ayyadurai VAS, Deonikar P. Do GMOs Accumulate Formaldehyde and Disrupt Molecular Systems Equilibria? Systems Biology May Provide Answers. Agric Sci. 2015;06:630–62. https://doi.org/10.4236/as.2015.67062

84. Kothandaram S, Deonikar P, Mohan M, Venugopal V, Ayyadurai VAS. <i>In Silico</i> Modeling of C1 Metabolism. Am J Plant Sci. 2015;06:1444–65. https://doi.org/10.4236/ajps.2015.69144

85. Mohan M, Kothandaram S, Venugopal V, Deonikar P, Ayyadurai VAS. Integrative Modeling of Oxidative Stress and C1 Metabolism Reveals Upregulation of Formaldehyde

and Downregulation of Glutathione. Am J Plant Sci. 2015;06:1527–42. https://doi.org/10.4236/ajps.2015.69152

86. Ayyadurai VAS, Hansen M, Fagan J, Deonikar P. In-Silico Analysis & In-Vivo Results Concur on Glutathione Depletion in Glyphosate Resistant GMO Soy, Advancing a Systems Biology Framework for Safety Assessment of GMOs. Am J Plant Sci. 2016;07:1571–89. https://doi.org/10.4236/ajps.2016.712149

87. Ayyadurai VAS, Deonikar P. Bioactive compounds in green tea may improve transplant tolerance: A computational systems biology analysis. Clin Nutr ESPEN. Elsevier Ltd; 2021;46:439–52. https://doi.org/10.1016/j.clnesp.2021.09.012

88. Ayyadurai VAS, Deonikar P, Bannuru RR. Attenuation of low-grade chronic inflammation by phytonutrients: A computational systems biology analysis. Clin Nutr ESPEN [Internet]. 2022;49:425–35. https://doi.org/10.1016/j.clnesp.2022.03.010

89. Cornish-Bowden A. One hundred years of Michaelis–Menten kinetics. Perspect Sci [Internet]. Elsevier; 2015;4:3–9. https://doi.org/10.1016/j.pisc.2014.12.002

90. Michaelis L, Menten ML, Goody RS, Johnson KA. Die Kinetik der Invertinwirkung/ The kinetics of invertase action. Biochemistry. 1913;

91. Hucka M, Finney A, Sauro HM, Bolouri H, Doyle JC, Kitano H, et al. The systems biology markup language (SBML): A medium for representation and exchange of biochemical network models. Bioinformatics. 2003;19:524–31. https://doi.org/10.1093/bioinformatics/btg015

92. Ayyadurai VAS. Services-Based Systems Architecture for Modeling the Whole Cell: A Distributed Collaborative Engineering Systems Approach. 2010;115–68. https://doi.org/10.1007/8754_2010_1

93. Shmulevich I, Aitchison JD. Deterministic and Stochastic Models of Genetic Regulatory Networks. Methods Enzymol. 2009;467:335–56. https://doi.org/10.1016/S0076-6879(09)67013-0

94. Oden JT (John T, Reddy JN (Junuthula N. An Introduction to the Mathematical Theory of Finite Elements. 1976th ed. New York: Dover Publications; 2011.

95. Cumming G, Fidler F, Vaux DL. Error bars in experimental biology. J Cell Biol. 2007;177:7–11. https://doi.org/10.1083/jcb.200611141

96. Thanh VH, Zunino R, Priami C. Efficient finite-difference method for computing sensitivities of biochemical reactions. Proc R Soc A Math Phys Eng Sci. The Royal Society Publishing; 2018;474. https://doi.org/10.1098/RSPA.2018.0303
